# Supplementary material for: The work of return to work. Challenges of returning to work when you have chronic pain: a meta-ethnography
Source: BMJ Open. 2019 Jun 20;9(6):e025743. doi: 10.1136/bmjopen-2018-025743 (PMC6596973; doi:10.1136/bmjopen-2018-025743)
Supplement: Supplementary data [file bmjopen-2018-025743supp001.pdf]

Supplementary File 1 - Search strategy

**Search terms**	(“Chronic pain” (MeSH) OR (Chronic adj5 pain) OR “Pain”)and<br>(“Return to work (MeSH) OR Employment (MeSH) or Employer OR Supported Employment” OR “Return-to-work” OR “Back to work” OR “Back-to-work” OR “Reemployment” OR “Re-employment” OR “Job” or “Work” OR “Reentry” or “Re-entry” or “Back” or “Return” OR “Employment, Supported” (MeSH) OR “rtw”) and “Qualitative”
**Databases searched**	AMED; ASSIA; CINAHL; EMBASE; IBSS; MEDLINE; PsycINFO; Social Services Abstracts; Sociological Abstracts; Web of Science; Westlaw<br><br>Forwards and backwards citation tracking using SCOPUS<br><br>Plus Social Care Online, PEDRO and OT Seeker
**Parts of journal searched**	Key words in abstract and title
**Years of search**	Inception to 25th April 2017
**Language**	English
**Types of study to be included**	Qualitative peer reviewed studies using face-to-face interviews and focus groups
**Inclusion criteria**	Studies exploring perception of obstacles to return to work in off work, sick-listed and employer populations of people with chronic pain.<br>Studies of people on disability benefits.
**Exclusion criteria**	Non-English language texts.

Supplementary File 2 – Critical Appraisal Skills (CASP) scores – yes =3, can't tell = 2, no=1

	**CASP questions (question 10 is not scorable)**									
**Article authors**	**1**	**2**	**3**	**4**	**5**	**6**	**7**	**8**	**9**	**Total score**
Ahlstrom *et al.* 2017	3	3	3	2	3	1	3	3	2	24
Andersen *et al.* 2014	3	3	3	3	3	1	3	3	3	27
Angel *et al.* 2012	3	3	3	2	3	1	3	2	3	23
Ashby *et al.* 2010	3	3	3	2	3	1	3	2	3	23
Brooks *et al.* 2013	3	3	3	3	3	1	3	3	3	25
Buus *et al.* 2015	3	3	3	3	3	3	3	3	2	26
Coole *et al.* 2010	3	3	2	3	3	2	3	2	2	23
Coutu *et al.* 2010	3	3	3	3	3	1	3	3	3	25
Coutu *et al.* 2011	3	3	3	2	3	1	3	3	3	24
Coutu *et al.* 2013	3	3	3	3	3	1	3	3	3	26
Crooks 2007	3	3	3	2	3	1	3	3	3	24
Dionne *et al.* 2013	3	3	3	2	3	1	3	3	3	24
Edén *et al.* 2007	3	3	3	3	3	1	3	3	2	24
Fassier *et al.* 2015	3	3	3	2	3	2	3	2	3	24
Gard & Sandberg 1998	3	3	3	2	3	1	1	2	3	21
Glavare *et al.* 2012	3	3	3	2	3	3	3	3	3	26
Grataloup *et al.* 2016	3	3	3	3	3	1	3	3	3	25
Hansson *et al.* 2001	3	3	3	2	3	1	3	3	3	24
Hansson *et al.* 2006	3	3	3	3	3	1	3	3	3	25
Johansson *et al.* 1997	3	3	3	3	3	3	1	3	3	25
Juuso *et al.* 2016	3	3	3	3	3	2	3	3	3	26
Kalsi *et al.* 2016	3	3	3	2	3	1	3	3	3	24
Kvam *et al.* 2013	3	3	3	3	3	3	3	3	2	26

Kvam & Vik 2015	3	3	3	3	3	1	3	3	3	25
Liedberg & Henriksson 2002	3	3	3	2	3	2	3	3	3	25
Magnussen *et al.* 2007	3	3	3	3	3	2	2	2	3	24
McCluskey *et al.* 2011	3	3	3	3	3	1	3	3	3	25
McCluskey *et al.* 2014	3	3	3	3	3	2	3	3	3	26
Nilsen & Anderssen 2014	3	3	3	3	3	1	3	3	3	25
Nordqvist *et al.* 2003	3	3	3	3	3	1	1	3	3	23
Patel *et al.* 2007	3	3	3	3	3	1	2	3	3	24
Rydstad *et al.* 2010	3	3	3	3	3	3	3	3	3	27
Saunders *et al.* 2015	3	3	3	3	3	2	3	3	3	26
Scheermesser *et al.* 2012	3	3	3	3	3	3	3	3	3	27
Shaw & Huang 2005	3	3	3	2	3	2	3	2	2	23
Sjöström *et al.* 2011	3	3	3	3	3	2	3	2	3	25
Soeker *et al.* 2008.	3	3	3	3	3	2	3	3	3	26
Soklaridis *et al.* 2010	3	3	3	2	3	2	3	3	2	24
Svensson *et al.* 2010	3	3	3	3	3	1	3	2	3	24
Williams-Whitt *et al.* 2016	3	3	3	2	3	1	3	3	3	24
Wynne-Jones *et al.* 2011	3	3	3	3	3	1	3	3	3	25

Supplementary file 3

The remaining 13 conceptual categories that underpin the three key categories described in the main paper and summarised in Table 2 are now explained.

Societal expectations

Many people living with chronic pain felt that society, institutions, family, friends and the media expected them to work and that those who did not do so were portrayed negatively and perceived as a burden.[47] This contributed to them feeling they were outsiders who were judged and discriminated against and for some this motivated them to return to work so they could contribute and belong.[36 59]

Meaning of work

The meaning of work for each individual plays a part in whether they return or not. For some work provides financial security and independence[45] but for others there is a strong moral work ethic influenced by their upbringing or it is a way of strengthening self-esteem[36] and achieving fulfilment in life.[48] A lack of meaning in work when combined with a chronic pain condition can lead to demotivation in relation to work return[59] whereas for some work was central to their identity and purpose and loss of this aspect of their lives was perceived as devastating.[39 42 44 52] Social contact and relationships with others and feeling needed and valued are an important aspect of work for some people.[48 61]

A number of conflicts were highlighted in this conceptual category. One was the differing perceptions of employees with low back pain and those of their employers. Some employers felt that employees perceived sick leave as a right and this formed part of a culture of entitlement they perceived was encouraged by the unions which made employees demotivated about returning to work.[60] However this was a view strongly contested by the employees in the same study who reported they would often accept modified jobs in order to return to work, even if not appropriate to them, due to the tough economic climate and fear of losing their livelihood.[60]

Another conflict was that of balancing the demands of work with those of family life. Competing priorities sometimes meant that some chose to focus on family rather than paid work as they were unable to balance the two.[46]

Autonomy

Concepts in this category focus on the individual's ability to have control or agency in relation to their pain and their work situation. There was a sense that if they had control over their pain then this was the key to having autonomy in other areas including return to work.[45] Being allowed some control and flexibility at work, for example in the hours they worked, was seen as a pre-requisite by some for returning.[36] Psychological distress, including anxiety and depression, was linked with a perceived lack of control over pain and as a result return to work became a secondary issue[12] whereas opportunity for job control was a motivating factor in relation to returning to work.[38]

Self-belief/ self-efficacy

Some studies indicated that people with chronic pain had low self-esteem and a pessimistic outlook about their ability to handle work[49] and this related to concerns about their ability to meet the job demands, obtain help from others and manage their pain.[57]

Health and illness and pain representations impact on return to work

The way people think about their pain and the mental representations they form in relation to beliefs about it's cause and their perception of its impact on their lives [29] are seen as very important in relation to their pain experience and return to work. A clear distinction was made between those who perceived themselves as disabled by the pain and therefore unable to work [28 31 32 44 50] and those who accepted the pain as part of their lives but something they could exert some control over and therefore felt able to work.[31 39 54] There was a perception amongst some employers that attitudes were influenced by family and the community in which people grew up.[60] However, among people with pain, there was a resistance to and even rejection of the idea of psychosocial influences on pain.[31 56] Some studies asserted

that the way people with pain, often incorrectly visualised their injuries and formulated explanatory models led to a fear of movement and this had negative implications for their work life.[27]

Influence of pre-return to work support and rehabilitation

A number of studies in the review were evaluating the impact of return to work interventions and rehabilitation programmes.[26 29 33 39 54] Participants were largely positive about the intervention received and the strategies for managing pain and life they had developed but those who did not return to work experienced anxiety, disappointment, loneliness [39] and some felt useless[56] or a sense of powerlessness and guilt.[26]

Not being understood

The concept of not being understood mainly arose when participants were describing difficulties in relationships with health professionals, for example physicians not understanding their work situation[59] or not listening or taking them seriously in relation to their pain.[52] However, the same phenomenon occurred even with people's closest relatives, leaving them with a sense of abandonment.[54]

Being believed

People with chronic pain often struggled with not being believed or trusted and this was evident when employers talked about people taking sick leave that was not perceived as legitimate.[63] This feeling of being judged and doubted and having to justify absence or limitations became an obstacle to returning to work.[37 59] The pursuit of authenticity also became apparent from the perspective of people claiming benefits.[50] These individuals felt the need to stress their desire to work but also to emphasise how the severity of their pain condition was preventing them from doing so.[50]

Impact of and on family

People with chronic pain often rely on family members for practical support[56 59] which leads to a renegotiation of roles and responsibilities[54] and financial difficulties are prevalent.[27]

Significant others are also seen to be highly influential in terms of their beliefs and thinking in relation to pain and return to work. Some studies have highlighted sceptical views of significant others in relation to treatment received and pessimism about return to work and support that would be offered[50 51] and sometimes their well-intentioned support reinforces a position of disability and legitimacy and this reduces the possibility of gaining employment.[28]

The conflicting demands of family and work were also reported as a challenge for people with chronic pain with women sometimes choosing to prioritise family.[48 47]

Mismatch between employee and employer expectations of return to work

Participants expressed a fear of letting employers down and not being able to fulfil work expectations.[12 58] Some were also fearful of re-injury.[57] Finding modified work was difficult and some felt the employer would rather dismiss them than find them a suitable job.[58] High demands for effectiveness and productivity made it difficult to return to work[49] as people with pain were concerned they would not be able to achieve the required quality, quantity or speed of work.[57] Information given to employers about health problems or limitations was perceived as insufficient or incorrect which led to misunderstandings and distorted employer expectations.[53] Difficulties arose in relationships with colleagues especially where expectations of employees with health problems were lower than for other employees doing the same job.[53] Participants felt they would want to do as much as their colleagues and not be a burden on them.[30 38 61]

Social isolation as a consequence of pain

One of the consequences of chronic pain was a withdrawal from social networks and this was partly linked with financial and physical restrictions.[27 48] This led to loneliness and a sense of being abandoned by those around and therefore lacking the support needed to enable return to work.[52 54]

System factors (healthcare, social security and workplace systems)

System factors influencing return to work were within healthcare, social security and workplace systems. Delays in accessing appropriate healthcare, for example waiting lists for specialists, diagnostic testing and rehabilitation programmes, interfered with the return to work process.[12 55 60] Another issue was that employers did not always feel they could accommodate injured workers hospital appointments in work time and so preferred that they remained off sick until they were able to fulfil their work hours.[60]

Social security authorities were sometimes seen as unhelpful and inflexible benefits arrangements caused economic uncertainty for people wanting to make a gradual transition into work.[49] Interactions with social insurance personnel were perceived as difficult with conflict arising when staff put pressure on people with pain to complete training or enter employment that was deemed unsuitable or not in line with their interests.[49 55]

Finally workplace systems delayed work return through inadequate policies[37] and a perceived lack of education on disability management procedures.[59] There also appeared to be a lack of trust in occupational health who were seen to be on the side of the employer and more concerned with absence management than supporting people to return to work.[30]

Finance and benefits

Many people with chronic pain had serious concerns about their finances.[41 43 56] For some this was linked with social security and disability benefits and the economic insecurity of moving back into work.[12 49] Some receiving state support struggled with shame whereas others felt they were entitled to it[43] or saw no alternative.[41] Some argued there should be greater flexibility for people with fluctuating musculoskeletal diseases to allow them to make a gradual return to work without incurring financial hardship.[34]

Supplementary File 4 - Concepts within each conceptual category

Conceptual category	Concepts
Managing pain	1. Adaptions via new knowledge of how to manage daily occupations<br>2. Being out in nature – pain management facilitator<br>3. Controlling pain<br>4. Coping with pain<br>5. Coping with symptoms<br>6. Developing individual strategies to deal with pain<br>7. Fatigue<br>8. Fluctuating work status<br>9. Keeping pain at bay<br>10. Knowledge of limits and listening to body<br>11. Learning to manage whiplash associated disorder - a rehabilitation process<br>12. Management of back pain<br>13. Previous management of back pain<br>14. Mastering life despite pain<br>15. New knowledge about how to manage daily life<br>16. Pain management linked with RTW<br>17. Pain management strategies<br>18. Pain representations underpin strategies to manage condition<br>19. Passive coping strategies<br>20. Patients' coping strategies<br>21. Physical activity<br>22. Strategies for managing long-term pain<br>23. Pain representations underpin strategies to manage condition<br>24. Passive coping strategies<br>25. Patients' coping strategies<br>26. Physical activity<br>27. Strategies for managing long-term pain<br>28. Strategies to prevent pain<br>29. Treatment for pain<br>30. Use of sick leave<br>31. When no treatment helps self-care management strategies develop<br>32. Working to control the pain<br>33. Being under control of pain and fear of pain<br>34. Commuting (as an obstacle to work)<br>35. Difficulties caused by back pain<br>36. Fear of reinjury<br>37. Impact of back pain on ability to work (for those at work and those not at work)<br>38. Impact of health on work<br>39. Impact of pain medication on ability to work<br>40. Impact of pain on doing work in a satisfactory way<br>41. Living with uncertainty<br>42. Negative impact of chronic pain on ability to work<br>43. Negative impact of pain on wellbeing and daily activities<br>44. Pain and somatic symptoms<br>45. Painful condition is a barrier to working<br>46. Persistent pain is an important obstacle to return to work

	47. Return to employment and fear of movement<br>48. The body as an obstacle to working (pain and fatigue)<br>49. Unpredictable pain difficult with respect to work
Managing relationships	1. Active engagement of supervisor<br>2. Asking for help is problematic for people with chronic pain<br>3. Attitude of employer in the workplace<br>4. Attitudes towards presenteeism (managers and employees perspectives)<br>5. Being needed at work<br>6. Colleague support important<br>7. Communication and contact (between managers and employees)<br>8. Co-workers and employers attitudes, disbelief and lack of understanding<br>9. Earlier negative workplace experiences<br>10. Employers limited understanding and support<br>11. Fear of disclosing pain to employer<br>12. Gap in work history and disclosure<br>13. Harassment from colleagues due to modified work<br>14. Impact of employees with job restrictions on supervisors and managers<br>15. Impact of sickness absence on others<br>16. Individuals (workers, colleagues, managers) barriers<br>17. Individuals facilitators (collaboration with colleagues support empathy problem solving mutual trust)<br>18. Interpersonal conflict with colleagues; being judged, justify the pain<br>19. Lack of collaboration and understanding from employer is an obstacle to return to work<br>20. Lack of communication between manager and the team about RTW<br>21. Lack of support and communication with line manager<br>22. Lack of support from line manager for injured workers<br>23. Lack of understanding from employer<br>24. Line manager role important<br>25. Maintaining contact with absent employee<br>26. Managerial attitude and effort<br>27. Managerial autonomy<br>28. Mutual distrust between employees and their managers and colleagues<br>29. Negative response from employer<br>30. Peer conflict<br>31. Physical barriers not significant obstacle to maintaining employment<br>32. Psychosocial environment (at work)<br>33. Psychosocial factors at work influence RTW management<br>34. Reassignment of workers to other areas due to physical demands of job causes tension with supervisors due to perceived injustice<br>35. Relationship between managers and employees<br>36. Relationship with employer<br>37. Relationships with supervisors and colleagues important to work satisfaction<br>38. Reluctance of employer to take on injured worker with gaps in employment history

	39. Responsibility for workmates<br>40. Social tensions in the workplace<br>41. Stigmatisation and blame<br>42. Struggling interactions with stakeholders<br>43. Support from employer and workmates<br>44. Supportive work environment<br>45. Supportive work environment and manager key to RTW success<br>46. Sympathy from manager if fellow back pain sufferer<br>47. Treatment from line manager inequitable<br>48. Understanding from an employer<br>49. Work relationships influence RTW<br>50. Working relations<br>51. Workmates attitudes
Making workplace adjustments	1. Being marked out as different in the workplace<br>Austerity and economic climate<br>2. Competitive economic climate with restructuring and workforce reduction is a barrier for RTW<br>3. Economic climate impacts on ability to take sick leave and make work adjustments<br>4. Fast management turnover, lack of latitude in decision making and fear of increasing costs and claims for better working conditions are barriers to RTW<br>5. Job availability and competitive job markets<br>6. Work restructuring (labour market)<br>Flexible working<br>7. Flexibility from employers re hours facilitates RTW<br>8. Flexible working a pre-requisite for RTW<br>9. Impossibility of a gradual return to work is an obstacle to return<br>10. Modified hours of employment<br>11. Policy and programme recommendations<br>12. Flexible work hours<br>13. Job sharing and work-from-home<br>14. Work place adjustments<br>Involve managers and colleagues<br>15. Communication quality<br>16. Maintaining routines for sharing information about work accommodations with colleagues<br>Manager knowing options<br>17. Absenteeism destabilises work organisation and makes work accommodations challenging<br>18. Accommodation demands<br>19. Job aptitude restriction certificates<br>20. Lack of pre-planning for RTW makes job accommodations and communication with colleagues challenging<br>21. Poor matching of the worker and the work<br>22. Process of accommodation of back injured workers<br>Resources for decision making about accommodations<br>23. Information - Accommodation options<br>24. Information - Employee abilities<br>25. Information - Job demands

	26. Information - Medical restrictions<br>27. Organisational support<br>28. Supervisors return to work experience<br>29. Size of workplace and difficulties of modified duties<br>30. Support from line managers over-cautious<br>31. Work modifications - assistance from Occupational Health<br>32. Work organisation and the challenges of work accommodations<br>Not consulted or involved in decision making.<br>33. Modified duties<br>34. Perceived lack of choice and control in relation to modified duties<br>35. Possibility of work adaptations and confidence and ability to negotiate adaptations with employer<br>36. Stakeholder perspective<br>37. Psychosocial stressors<br>38. Work modifications - patient control<br>Personal factors<br>39. Age and educational status - perceived as obstacle to finding work by people with back injury<br>40. Personal obstacles - qualifications and experience<br>41. Personal obstacles to RTW - older age<br>42. Resistance to change<br>Reducing demands of job or physical adjustments<br>43. Adjustment of work demands upon return to work<br>44. Challenges to work participation (to different type of work and work adjustments)<br>45. Change to less physically demanding job<br>46. Impossibility of being assigned lighter duties and working at one's own pace is an obstacle to return to work<br>47. Improvement of work environment and working conditions<br>48. Physical accommodations<br>49. Provision of appropriate modified work can be challenging and complex<br>50. Work modifications influence RTW possibilities<br>51. Working conditions - physical work<br>Type of job influences RTW<br>52. Benefits of self-employment<br>53. Physical stressors (working at lower surface areas and different weights) cause constant pain<br>54. Sharing staff over different departments makes work accommodations and assessment of work demands difficult<br>55. Type of job- profession influences RTW
Autonomy	1. Autonomy<br>2. In the hands of the professionals - reduced control over life situation<br>3. Increased job control and contact with supervisors<br>4. Influencing factors for RTW - internal and external<br>5. Lack of agency<br>6. Locus of control influences return to work<br>7. Making sense of intervention - regaining control of situation<br>8. Own agency is important

	9. Own power and resources<br>10. Perceived lack of control influences ability to work<br>11. Psychological barriers to return to work (lack of control, anxiety, depression, loss of confidence, frustration)<br>12. Taking control of and responsibility for work and life situation
Self-belief/ self-efficacy	1. Being needed at work<br>2. Changed self-image<br>3. Controlling RTW interactions with stakeholders (employer, health care, social insurance system, union, public employment service)<br>4. Low self- evaluation of work ability and low self esteem<br>5. Obtaining help at work<br>6. Patient identity (when working and not working)<br>7. Positive coping linked with RTW self-efficacy<br>8. Positive self-identity a beneficial consequence of employment<br>9. Psychological effects of chronic pain affect RTW confidence<br>10. Relationship with family influences self-confidence and esteem<br>11. Satisfaction with self-image; confidence<br>12. Self-confidence through working<br>13. Self-efficacy<br>14. Self esteem<br>15. Self-image in relation to work<br>16. Self-identity<br>17. Unintended consequences - physical bodily changes post-injury like weight gain affect emotional readiness to return to work<br>18. Work morale
Being believed	1. Being judged by colleagues<br>2. Disbelief from physicians<br>3. Distrustful attitude of the medical profession<br>4. Feeling doubted<br>5. Having to justify pain condition in the workplace<br>6. Legitimacy of absence and perceptions of others<br>7. Legitimising back pain<br>8. Legitimacy<br>9. Not being believed<br>10. Personally and socially legitimate explanations of LBP important<br>11. Pursuit of authenticity<br>12. Rights and responsibilities<br>13. Stigmatisation
Impact on and of family	1. Being a 'good' significant other<br>2. Cultural differences in family support between women and men<br>3. Family support<br>4. Impact of family<br>5. Loss of social roles<br>6. Participation in work - a family matter<br>7. Relationship changes<br>8. Re-negotiation or loss of work role<br>9. Return to work is dependent on a cure (significant other viewpoint)

	10. Role of significant others is important in return to work<br>11. Unpaid work (home, family, carer responsibilities)<br>12. Waiting for an answer (diagnosis, treatment, cure) (significant other viewpoint)<br>13. We have come to the end of the road (treatment options exhausted) (significant other viewpoint)
Not being understood	1. Communication in rehab - cultural differences cause problems<br>2. Language barriers<br>3. Talking at cross purposes with health professionals<br>4. Congruence between clinicians understanding of workers representations and actual worker's representations during work rehab<br>5. Cultural factors influence RTW - family attitudes, language barriers, cultural beliefs<br>6. Differences between clinical judgement and workers representations during work rehab<br>7. Difficult to explain the pain<br>8. Lack of client centredness<br>9. Medical discourse of work participation - focus on pain rather than RTW
Finance and Benefits	1. Finances<br>2. Financial concerns<br>3. Financial - job security<br>4. Interaction with benefits organisation<br>5. Permitted work<br>6. Limited staff skills in benefits organisation<br>7. Looking for a different way of living - transition to disability pension<br>8. Need for financial security<br>9. Part-time work by people receiving disability income assistance<br>10. State as supporter
Health and illness and pain representations	1. Acceptance challenges - Difficulties in acceptance of pain and limitations impacts on participation in work<br>2. Acceptance of chronic pain as a long term disability is a barrier to return to work<br>3. Acceptance of limitations<br>4. Acceptance of pain as part of life facilitates RTW<br>5. Accepting the inability to work<br>6. Barriers to rehabilitation<br>7. Cultural influence on psychological factors<br>8. Beliefs about causality of back pain<br>9. Beliefs about course of illness and the sick role<br>10. Beliefs about treatment and effective management of LBP<br>11. Cause and meaning of back pain<br>12. Crystallising the abnormal pain representation<br>13. Cultural factors influence RTW - family attitudes, language barriers, cultural beliefs<br>14. Cultural factors influencing return to work (language and passive coping strategies)

	15. Explanatory models of illness<br>16. Illness beliefs coherent<br>17. Impact of pain representations on return to work<br>18. Integrating explanations into daily life<br>19. Loss of ability<br>20. Loss of hope<br>21. Need to construct their own models of pain<br>22. Resignation to permanent effect of back problem on employment status in those not working and their significant others
Meaning of work	1. Competing priorities mean work not necessarily prioritised<br>2. Effort to remain in or return to pre-injury jobs<br>3. Effort to return to employment following job loss<br>4. Employee motivation<br>5. Family orientated considerations take priority over working<br>6. Fulfilment in a work role<br>7. Goal orientated participation (work related achievements and values)<br>8. Importance of work<br>9. Lack of meaning and satisfaction in work<br>10. Meaning of work<br>11. Meaningful job- highly needed by others<br>12. Mentality (outlook) in relation to determination to RTW<br>13. Moral aspects of absence and presenteeism<br>14. Moral stance - importance of work<br>15. Motivation and entitlement<br>16. Motive for RTW<br>17. Organised time structure difficult to maintain without work<br>18. Participation constantly changing (feminine perspective)<br>19. Positive perceptions of work<br>20. Prioritising of work and home is an issue<br>21. Regaining identity (as a worker)<br>22. Sense of coherence<br>23. Sense of coherence and involvement in work, friends and family<br>24. Social aspects of work<br>25. Work as a source of security and independence<br>26. Work on hold
Mismatch of expectations	1. Ability to do as much work as others<br>2. Fear of letting employers down<br>3. Fear of re-injury<br>4. Insufficient or incorrect information about health problems of the returning employee<br>5. Meeting job demands<br>6. Not able to fulfil work requirements<br>7. Own expectations in relation to RTW (optimistic or pessimistic)<br>8. Participating at before (masculine perspective)<br>9. Support expectations<br>10. Workplace productivity demands<br>11. Workplace support

Social isolation as a consequence of chronic pain	1. Abandoned by those around (family, friends and colleagues)<br>2. Feeling on their own<br>3. Impact on social relations - many women with fibromyalgia fail to maintain social network due to demands of work and family<br>4. Loneliness in pain<br>5. Paid work - the struggle for social capital<br>6. Social isolation
Influence of return to work support and rehabilitation	1. “A light at the end of the tunnel”; support, hope, new knowledge<br>2. A light in the tunnel - experience of work rehab programme<br>3. Hope of returning to work through rehab<br>4. Support<br>5. Believing in the intervention - effectiveness of exercise to manage LBP<br>6. Close social network – family, rehab team<br>7. Difficulty accessing worker representations and problem targeting in work rehab<br>8. Feelings about outcome of rehab<br>9. Function-centred treatment<br>10. Patients' expectations of treatment<br>11. Goals of rehabilitation - patient's perspective<br>12. Goals of rehabilitation - return to work<br>13. Joining a physical exercise programme<br>14. Lack of access to information or support groups is an obstacle to return to work<br>15. Mismatch of goals - patient and programme<br>16. Rehabilitation by activity and exercise<br>17. Specialised vocational rehab support needed<br>18. Support is important<br>19. Unsuccessful responses to intervention (rehabilitation)
System factors	*1. Health care system*<br>2. Healthcare barriers<br>3. Lack of communication, lack of coordination and fear of communication within compensation and health care systems is a barrier to RTW<br>4. Lack of knowledge in primary care and no support from social insurance office<br>5. Slowness of health care system is an obstacle to return to work<br>*6. Social security, insurance, unemployment office system*<br>7. From Social Insurance Office<br>8. From unemployment office<br>9. Inefficiency of the insurance companies<br>10. Lack of support from social security authorities<br>*11. Workplace system*<br>12. Inadequate workplace policy<br>13. Lack of education on disability management procedures by employers and rehabilitation professionals<br>14. Occupational health is for employers not employees<br>15. OH employer orientated - unequal relationship<br>16. Organisational policies - Return to work policies

	17. Poor communication between stakeholders (doctors and employers about back condition and lighter duties duration)<br>18. Systems factors - workplace and union policies compensation system and healthcare system are barriers to RTW process<br>19. Wage support programmes awarded to employee<br>20. Workplace system barriers<br>21. External context barriers<br>22. Workplace barriers<br>23. Workplace system facilitators<br>24. External context facilitators<br>25. Workplace facilitators
Societal expectations	1. Experiences of societal expectations of participation in work<br>2. Feeling of being outsider in society; work part of natural life<br>3. Unsupportive society
