# Supplementary material for: The work of return to work. Challenges of returning to work when you have chronic pain: a meta-ethnography
Source: BMJ Open. 2019 Jun 20;9(6):e025743. doi: 10.1136/bmjopen-2018-025743 (PMC6596973; doi:10.1136/bmjopen-2018-025743)
Supplement: Author's manuscript [file bmjopen-2018-025743.draft_revisions.pdf]

BMJ Open

BMJ Open is committed to open peer review. As part of this commitment we make the peer review history of every article we publish publicly available.

When an article is published we post the peer reviewers' comments and the authors' responses online. We also post the versions of the paper that were used during peer review. These are the versions that the peer review comments apply to.

The versions of the paper that follow are the versions that were submitted during the peer review process. They are not the versions of record or the final published versions. They should not be cited or distributed as the published version of this manuscript.

BMJ Open is an open access journal and the full, final, typeset and author-corrected version of record of the manuscript is available on our site with no access controls, subscription charges or pay-per-view fees (http://bmjopen.bmj.com).

If you have any questions on BMJ Open's open peer review process please email info.bmjopen@bmj.com

BMJ Open

# The work of return to work. Challenges of returning to work when you have chronic pain: a meta-ethnography



| | |
|---|---|
| Journal: | *BMJ Open* |
| Manuscript ID | bmjopen-2018-025743 |
| Article Type: | Research |
| Date Submitted by the Author: | 03-Aug-2018 |



| | |
|---|---|
| Complete List of Authors: | Grant, Mary; University of Warwick Warwick Medical School, Clinical Trials Unit<br>O-Beirne-Elliman, Joanne; University of Warwick Warwick Medical School, Clinical Trials Unit<br>Froud, Robert; University of Warwick Warwick Medical School, Clinical Trials Unit; Kristiana University College, Prinsens gate 7-9, 0152 Oslo, Norway, Department of Health Sciences<br>Underwood, Martin; Warwick University, Warwick Medical School<br>Seers, Kate; University of Warwick Warwick Medical School, Warwick Research in Nursing |



| | |
|---|---|
| Keywords: | PAIN MANAGEMENT, occupational health, return to work, metaethnography |


Note: The following files were submitted by the author for peer review, but cannot be converted to PDF. You must view these files (e.g. movies) online.

RISE metaethnog - Enhancing transparency in reporting the synthesis of qualitative research.docx

SCHOLARONE™ Manuscripts

# The work of return to work. Challenges of returning to work when you have chronic pain: a meta-ethnography

**BMJ OPEN**

**Corresponding author:**

Mary Grant, Warwick Clinical Trials Unit, University of Warwick, Coventry, UK

M.Grant.2@warwick.ac.uk

**Co-authors:**

Joanne O'Beirne-Elliman, Warwick Clinical Trials Unit, University of Warwick, Coventry, UK

Robert Froud, Warwick Clinical Trials Unit, University of Warwick, Coventry, UK and Institute of Health Sciences, Kristiania University College, Oslo, Norway.

Martin Underwood, Warwick Clinical Trials Unit, University of Warwick, Coventry, UK

Kate Seers, Warwick Research in Nursing, Warwick Medical School, University of Warwick, Coventry, UK

**Key words: chronic pain, return to work, meta-ethnography**

For peer review only

**ABSTRACT**

**Aim:** To understand obstacles to returning to work, as perceived by people with chronic non-malignant pain, and as perceived by employers.

**Design:** Synthesis of qualitative research using meta-ethnography.

**Data sources:** Eleven bibliographic databases from inception to April 2017 supplemented by citation tracking.

**Review methods:** We used the methods of meta-ethnography. We identified concepts, conceptual categories and developed a conceptual model and line of argument.

**Results:** We included 41 studies. We identified three core categories in the conceptual model; managing pain, managing work relationships and making workplace adjustments. All were influenced by societal expectations in relation to work, self (self-belief, self-efficacy, legitimacy, autonomy and the meaning of work for the individual), health/ illness/ pain representations, pre-return to work support and rehabilitation, and system factors (healthcare, workplace and social security). A mismatch of expectations between the individual with pain and the workplace contributed to a feeling of being judged, and difficulties asking for help. The ability to navigate obstacles and negotiate change underpinned mastering return to work despite the pain. Where this ability was not apparent, there could be a downward spiral resulting in not working.

**Conclusions:** For people with chronic pain, and for their employers, navigating obstacles to return to work entails balancing the needs of: (1) the person with chronic pain; (2) work colleagues; and (3) the employing organisation. Managing pain, managing work relationships and making workplace adjustments are central, but not straightforward, and require substantial effort to culminate in successful return to work.

**[Word count = 244]**

For peer review only

***Strengths and Limitations of this study*** *(five bullet points - one sentence each)*

Strengths

- This is the first study to present employer and employee perspectives together.
- This study draws together what is known from qualitative studies to inform practice.
- This study highlights health and illness and pain representations in relation to return to work.

Limitation

- Only five studies covered employers' perspectives, so there are fewer data on employers' perspectives compared to the perspectives of people with chronic pain.

For peer review only

# INTRODUCTION

Chronic pain, defined as pain lasting three months or more[1], is a global public health problem affecting one-in--ten adults.[2] A 2017 mega-ethnography brought together eleven qualitative evidence syntheses to explore the experience of living with chronic non-malignant pain.[3] Previous reviews have identified the importance of the effect of chronic pain on people's work life.[4 5] Chronic pain is strongly associated with claiming disability and unemployment benefit in Australia[1] and with unemployment in the USA.[6] The obstacles to staying in work for people with musculoskeletal pain have previously been explored in a meta-ethnography[7] and factors promoting staying at work are the focus of a previous mixed methods systematic review.[8] A qualitative systematic review of the impact of chronic pain in the workplace[9] takes a broad perspective including impact on employment status, sickness absence and loss of productivity in contrast to a condition. A gender-specific literature review focused on work and rehabilitation for women with fibromyalgia.[10] The lack of focus on return to work for people with chronic non-malignant pain and the perspective of employers presents a knowledge gap in existing reviews. Return to work can refer to the process of returning after a period of sick leave[11] or returning after a period of unemployment.[12] This review uses qualitative evidence synthesis to increase understanding of the obstacles to return to work for people with chronic pain and their employers, and this can then inform intervention development to support return to work.[4 13]

# METHODS

## Aims and objectives

This meta-ethnography explores experiences of returning to work, as perceived by people with chronic non-malignant pain and by employers.

## Study design

For peer review only

There are two main approaches to synthesising qualitative research, one that aggregates findings to describe the literature and one that aims to interpret findings and develop a conceptual understanding.[4 13 14] Meta-ethnography is an interpretative form of knowledge synthesis that was chosen for this study in order to both integrate and develop a greater understanding of existing knowledge and identify any other overarching concepts that would explain the data. The seven phases of meta-ethnography are outlined by Noblit & Hare (1988)[14] and elaborated on by Toye *et al.* (2014b).[13] These are 1) getting started by identifying area of interest 2) deciding what is relevant 3) reading and re-reading the studies 4) determining how the studies are related which involves creating a list of key phrases, ideas, metaphors and concepts 5) translating the studies into one another where direct comparisons are made and similar concepts are sorted into categories 6) synthesising the translations where researchers make sense of the conceptual categories to develop new knowledge and understanding and 7) expressing the synthesis. A line of argument was constructed by examining how the conceptual categories relate to each other.

**Identifying and appraising the review articles**

**Search methods**

**Study selection**

Eleven electronic bibliographic databases were searched (AMED; ASSIA; CINAHL; EMBASE; IBSS; MEDLINE; PsycINFO; Social Services Abstracts; Sociological abstracts; Web of Science; Westlaw) from inception up until 25th April 2017 supplemented by backwards and forwards citation tracking using SCOPUS. An academic support librarian undertook the initial search in collaboration with RF in December 2016 and this was updated by MG in April 2017. The search terms used included “Chronic pain” and “Return to work (MeSH) OR Employment OR Employer OR Supported Employment (MeSH)”. In April 2017 two additional search terms were used, ‘pain”’ to broaden search as ‘chronic pain”’ was not identifying all relevant papers and “‘qualitative”’ to focus the search on studies with this type of methodology. All qualitative studies using face to face interviews and focus groups which explored perceptions of obstacles to return to work, in employers and people who were off work, sick-listed and had chronic pain were included. Non-English language texts were excluded.

For peer review only

## Quality appraisal

The quality of studies was evaluated using the Critical Appraisal Skills Programme (CASP) qualitative assessment tool.[15] A scoring system was utilised for CASP (yes = 3, can't tell = 2, no = 1). A score of 20 or higher indicates the paper is deemed to be of satisfactory quality. The GRADE-CERQual (Grading of Recommendations Assessment Development and Evaluation - Confidence in the level of Evidence from Reviews of Qualitative research) approach was also completed.[16 17] Confidence in review findings was assessed based on four components: adequacy of data[18], coherence[19], methodological limitations[20] and relevance.[21]

## Analysis

Initially, the first ten papers (in alphabetical order of author) were read by MG, KS and JOB in order to identify key 'concepts', the raw data of meta-ethnography.[13] These concepts are ideas drawn from the findings of the original papers. They are also known as second order concepts because they are the authors' interpretations of the participant's narratives (known as first-order concepts).[22] The participants' narratives chosen by the author are examples of second-order concepts.[13] After reading these 10 papers, the concepts identified by each researcher were amalgamated through discussion and grouped into conceptual categories that the team then worked collaboratively to name. This took place over a series of three meetings. These conceptual categories are third-order concepts insofar as they are the researchers' interpretations of second order concepts. This is the way that studies were translated and related to each other. The first author then proceeded to read the rest of the papers and continue this process of analysis. Five additional papers were also read by KS and JOB where MG felt a collaborative discussion would be helpful due to the nature and/ or findings of the studies. All the included papers were uploaded to QSR International's NVivo 11 software[23] and nodes were created for the conceptual categories. The next stage was to make sense of these categories through further discussion, make decisions about which were the core categories and develop a line of argument and conceptual model[13] involving a

For peer review only - http://bmjopen.bmj.com/site/about/guidelines.xhtml

further four meetings. The line of argument makes a whole of something more than a sum of the parts.[14] MG, JOB and KS independently drew their own conceptual model before coming together to agree a model, which was revised through several discussions and the final version (the 11th version) which is presented in this paper. The culture described by Toye *et al.* (2013b)[24] of a core team that provided a safe environment in which to freely discuss, agree, disagree and change their position in relation to conceptual analysis and this was seen as a key strength, laying the foundations for a rigorous review. This approach was adopted in this review.

**Patient and Public Involvement**

A patient and public representative was involved in the development of the research funding submission for the overall study as a co-applicant and endorsed the importance of the focus of this meta-ethnography.

**Results**

**Search outcome and overview of studies reviewed**

We include 41 papers (**see Figure 1 [insert near here]**). The initial 3191 hits were screened by titles and abstracts, duplicates excluded and a further 1466 were excluded at this stage. Following the reading of full texts, papers were excluded as they were neither about chronic pain nor specifically about return to work. All the included studies passed the first two screening questions of the CASP tool that related to whether there was a clear statement of the aims of the research and if qualitative methodology was considered appropriate to address the research goal.[15] CASP scores are presented in Supplementary file 1. Of the 41 articles included, 32 reported interview studies and nine focus group studies. Twenty-one studies were from Scandinavia (14 in Sweden, four in Norway, and three in Denmark), seven were from the UK, seven were from Canada, two in France and one each from Australia, South Africa, Switzerland and USA. Only five studies were from employer's perspectives. One study included in the review did not specify the type of chronic pain, but the majority of

For peer review only

the studies involved people or employers of people with musculoskeletal pain, mainly affecting the back and neck and some were injury/ work related. Studies of people with musculoskeletal disease including arthritis, fibromyalgia and systemic lupus erythematosus were also included (Table 1).

For peer review only

165 **Table 1 Description of included studies**

166

| | Author and year of publication | Country | Type of pain | Number, gender and age of participants | Participants | Method of data collection | Methodological approach – Analysis |
|---|---|---|---|---|---|---|---|
| 1 | Ahlstrom *et al*. 2017[11] | Sweden | Neck pain | 16 women<br>Mean age of 54 | People with history of long-term sick leave in human service organisations | Interviews | Constructivist grounded theory approach |
| 2 | Andersen *et al.* 2014[25] | Denmark | Back or upper body | 4 men and 3 women aged 33-57 | Participants in chronic pain self-management programme or tailored physical activity programme | Semi-structured interviews | Systematic text condensation – thematic cross case analysis |
| 3 | Angel *et al*. 2012[26] | Denmark | Low back pain | 20 (65% women)<br>Mean age of 46 | Participants of counselling intervention addressing work place barriers and physical activity | Semi-structured clinical interviews | Narrative analysis |
| 4 | Ashby *et al.* 2010[27] | Australia | Chronic low back pain | 11 men aged 23-59 | Participants in a work hardening programme | Semi-structured interviews (ethnographic) | Thematic content analysis |
| 6 | Brooks *et al*. 2013[28] | England | Persistent non-specific low back pain of at least 12 weeks duration | 6 women and 3 men<br>Working participants (5) aged 45-52 (mean 49.2)<br>Unemployed participants (4) aged 51-63 (mean 57) and their significant | Participants from hospital pain management clinic | Semi-structured interviews | Template analysis style of thematic analysis |

For peer review only

| | | | | | | | |
|---|---|---|---|---|---|---|---|
| | | | | others | | | |
| 7 | Buus *et al.* 2015[29] | Denmark | Low back pain | 25 (56% women) (mean age 46.8) | People who had received counselling intervention designed to motivate them to change work routines and exercise | Semi-structured interviews | Interpretative thematic analysis |
| 8 | Coole *et al.* 2010[30] | UK | Low back pain | 13 women and 12 men aged 22-58 years (mean age 44.7) | People offered MDT back pain rehabilitation and concerned about ability to work due to low back pain | Semi-structured interviews | Thematic analysis |
| 9 | Coutu *et al.* 2010[31] | Canada | Persistent musculoskeletal pain - back pain (10), upper extremities (4), mixed (2) | 10 men and 6 women aged 25-56 (mean age 40) | Workers referred to work rehab programme | Semi-structured interviews | Narrative approach – content analysis |
| 10 | Coutu *et al.* 2011[32] | Canada | MSD –related pain for more than 12 weeks accepted and compensated by Quebec Workers Compensation Board<br><br>Back pain (10), upper extremities (4), mixed (2) | 16 workers - 10 men six women<br><br>Aged 25-56 years (mean age 40) | Referred to evidenced based work rehab programme by a third party payer | Semi-structured interviews | Content analysis |

For peer review only

| | | | | | | | |
|---|---|---|---|---|---|---|---|
| 11 | Coutu *et al.* 2013[33] | Canada | MSD –related pain for more than 12 weeks accepted and compensated by Quebec Workers Compensation Board<br>Back pain (8), upper extremities (2), both (2) | 12 workers (8 men and 4 women) aged 25-56 (mean age 31) and five clinicians | Participants from workers starting a work rehab programme at a hospital research centre | Multiple case study design- semi-structured interviews with workers and rehab clinicians at four points in time | Thematic analysis & constant comparison method (grounded theory) |
| 12 | Crooks 2007[34] | Canada | MSD (fibromyalgia, arthritis, RA, OA, Lupus) | 18 women aged 26-69 (mean age 44) | Women who developed MSD while involved in the labour market | In-depth interviews | Thematic analysis |
| 13 | Dionne *et al.* 2013[35] | Canada | Work-disabling back pain | Workers with work-disabling back pain<br>9 = returned to work (7 men, 2 women) aged 30-59<br>10= not returned or recently returned (7 men, 2 women) aged 30-60+ | Recruited through newspaper adverts | Focus groups (two) | Content analysis |
| 14 | Edén *et al.* 2007[36] | Swedish | MSD (type not specified) | 17 individuals (two men, 15 women) aged 41-62 years | People going back to work by means of the Swedish 'resting disability pension' | Interviews | Inductive analysis relevant to research question |
| 15* | Fassier *et al.* 2015[37] | France | Low back pain | Three employers, | Recruited from work | Interviews and | Qualitative content |

For peer review only

| | | | | | | | |
|---|---|---|---|---|---|---|---|
| | | | | one manager and one worker | places with high rates of absence for low back pain – car maker, association providing home services for the dependent and two university hospitals | focus groups | analysis |
| 15 | Gard & Sandberg 1998[38] | Sweden | Musculoskeletal pain (shoulder, neck and low back pain) for at least one year with a period of at least four weeks during that time | 10 patients (9 females and one male) aged 30-54 years (mean age 47) | People sick listed with musculoskeletal pain | Interviews with a low degree of structure | Phenomenological structural analysis |
| 16 | Glavare *et al.* 2012[39] | Sweden | Long-term musculoskeletal pain (whiplash, fibromyalgia, nerve injury (neck), arthrosis of foot) | 11 (8 women and 3 men) aged 22-58 (median age 39) | Participants in a multi-professional pain rehab programme followed by a coached work-training programme | Thematised research interviews | Grounded theory – constant comparative method |
| 17* | Grataloup *et al.* 2016[40] | France | Supervisors of people with musculoskeletal disorders | Employees' supervisors (61 charge nurses, and head nurses supervising one or more workers with restrictions for heavy lifting or repetitive movements) | Staff from three public hospitals | 12 focus groups (charge nurses and head nurses separate) | Thematic qualitative analysis by constant comparison each focus group analysed before the next held |
| 18 | Hansson *et al.* 2001[41] | Sweden | Spine-related | 5 people (4 female | People granted | Interviews | Based on grounded |

For peer review only

| | | | | | | | |
|---|---|---|---|---|---|---|---|
| | | | pain | and 1 male) aged 51-64 (median 55) | disability pension in 1996 | conducted as conversations – approach based on symbolic interactionism | theory |
| 19 | Hansson *et al.* 2006[42] | Sweden | Neck or low back pain (spine related pain) | 33 (20 women and 13 men) aged 32-61 years (median age 48) | Sick listed participants | Qualitative interviews | Qualitative analysis |
| 20 | Johansson *et al.* 1997[43] | Sweden | Undefined musculoskeletal pain disorders | 20 female patients aged 21 – 61 | Females sick listed due to MSD in urban health centre | Repeated thematic interviews | Grounded theory |
| 21 | Juuso *et al.* 2016[44] | Sweden | Fibromyalgia | 15 women aged 38-64 (median 54) | From a rehabilitation centre (4), associations for rheumatism and FM (11) | In-depth qualitative interviews | Hermeneutic approach |
| 22 | Kalsi *et al.* 2016[45] | UK | Chronic pain (type not specified) | 17 patients (8 male, 9 female) aged 18-65+ but majority (14/17) were 18-34 | Patients attending a 3 week high intensity pain management programme | Semi-structured focus group discussion | Thematic analysis |
| 23 | Kvam *et al.* 2013[46] | Norway | Prolonged musculoskeletal pain (unspecified pain in back, neck and shoulders due to fibromyalgia, arthritis, and rheumatism) | 4 men and 6 women aged 26-57 | Volunteers from people undergoing vocational rehabilitation | Semi-structured interviews | Constant comparative analysis |
| 24 | Kvam & Vik 2015[47] | Norway | Prolonged musculoskeletal | 6 women, 4 men aged 26-57 | People undergoing vocational | In-depth interviews | Discourse analysis |

For peer review only

| | | | | | | | |
|---|---|---|---|---|---|---|---|
| | | | pain (unspecified pain in back, neck and shoulders due to fibromyalgia, arthritis, and rheumatism) | | rehabilitation | | |
| 25 | Liedberg & Henriksson 2002[48] | Sweden | Fibromyalgia | 39 women aged 35-63 (mean 49.5) | Patients from a pain and rehab centre | Interviews | Analysed into categories and subcategories |
| 26 | Magnussen *et al.* 2007[49] | Norway | Back pain | 12 women, 5 men aged 38-56 years (mean age 49) | Part of a larger study evaluating the effect of a vocational rehabilitation related intervention | Three focus groups | Analysis of themes and subthemes |
| 27 | McCluskey *et al.* 2011[50] | UK | Persistent back pain | 5 dyads (4 male & 1 female claimants) – aged 29-54 years (mean age 40.2) | Disability claimants and their significant others | Semi-structured interviews | Template analysis |
| 28 | McCluskey *et al.* 2014[51] | UK | Persistent low back pain | 18 (9 benefits claimants - 5 males and 4 females aged 29-63 - mean age 48.1) and 9 significant others (6 females and 3 males aged 21-68 mean age – 49.7) | Work disability benefits claimants and significant others | Semi-structured interviews | Template analysis |
| 29 | Nilsen & Anderssen 2014[52] | Norway | Non-malignant chronic pain (neck and back pain, traffic | 10 men and 10 women aged 26-63 (in the year 2006) - mean age 42.7 | From a specialist pain clinic | Open ended interviews | Narrative analysis Phenomenological meaning condensation |

For peer review only

| | | | | | | | |
|---|---|---|---|---|---|---|---|
| | | | injuries) | | | | framework |
| 30 | Nordqvist *et al.* 2003[53] | Sweden | Back, neck or shoulder diagnoses | 13 women and 5 men | People who in 1985 were 25-34 years old and had a new sick leave spell of at least 28 days. | Five focus groups | Grounded theory coding and categorising |
| 31 | Patel *et al.* 2007[12] | UK | Chronic musculoskeletal pain | 38 patients (15 male 23 female) aged 29-62 years (mean age 49.4) | Unemployed and in receipt of long-term social welfare benefits | In-depth semi-structured interviews | Framework approach and thematic analysis |
| 32 | Rydstad *et al.* 2010[54] | Sweden | Whiplash Associated Disorders | 9 people (5 females, 4 males) aged 32-53 years | Participants of a work-oriented MDT rehab programme | Thematised interviews | Constant comparison method – grounded theory |
| 33 | Saunders *et al.* 2015[55] | Canada | MSK injury<br><br>Arm (1), knee (1), back injuries(7) | 9 people (5 females, 4 males) aged 34-56 years | People with long-term work disability and job loss due to an MSK injury from work rehab and chronic pain programmes | Interviews (27) with 9 people | Thematic analysis (phenomenological approach guided by life world concept) |
| 34 | Scheermesser *et al.* 2012[56] | Switzerland | Low back pain | 13 (9 men, 4 women) aged 38-60 years (mean age of men – 52, mean age of women 48) | Patients with a Southeast European cultural background attending a rehab centre in Switzerland | In-depth semi-structured interviews (5) & two focus groups | Qualitative content analysis |
| 35 | Shaw & Huang 2005[57] | USA | Occupational low back pain | Focus group - 28 people (15 male, 13 female) aged 31-65 (mean age 46)<br><br>Interviewees – 23 people (11 male, 12 | Focus group participants - people recently (<6 months) returned to work after injury responding to newspaper advert | Focus group & interviews | Content analysis |

| | | | | | | | |
|---|---|---|---|---|---|---|---|
| | | | | female) aged 25-64 (mean age 42.6) | Interview participants - patients referred by physios from collaborating OH network | | |
| 36 | Sjöström *et al.* 2011[58] | Sweden | MSK disorders - mainly back and neck pain | 10 people (7 women, 3 men) aged 29-61 (mean age 48) | Attended a rehab programme and still on full time sick leave 2 years after completion | Semi-structured interviews | Qualitative content analysis |
| 37 | Soeker *et al.* 2008 [59] | South Africa | Back injury | 26 people (18 males, 8 females) aged 18 – 60 | Selected by random sampling from a hospital rehab department | Focus groups | Qualitative analysis |
| 38* | Soklaridis *et al.* 2010[60] | Canada | Low back pain – work-related injury | 59 stakeholders including 6 injured workers and 5 small and 9 large employers | Various contacts of the research team | Nine focus groups | Grounded theory approach |
| 39 | Svensson *et al.* 2010[61] | Sweden | Back neck or shoulder diagnosis | 13 women and 5 men | People aged 25-34 years old in 1985 and had a new sick leave spell of at least 28 days | Five focus groups | Descriptive and explorative method of analysis |
| 40* | Williams-Whitt *et al.* 2016[62] | Canada | Low back pain | 23 supervisors | Supervisors of back injured workers from 11 Canadian organisations | Semi-structured in-depth interviews | Constructivist grounded theory principles |
| 41* | Wynne-Jones *et al.* 2011[63] | Wales | Musculoskeletal pain | 18 employees with MSK pain (8 male, 10 female) mean age 49.7 | Two large public sector organisations | Semi-structured interviews | Thematic analysis |

For peer review only

| | | | | | | | |
|---|---|---|---|---|---|---|---|
| | | | | 20 managers (10 male, 10 female) mean age 44.8 | | | |

*Employer studies

For peer review only

**Overarching conceptual categories**

A total of 342 concepts were clustered into 16 conceptual categories summarised in Table 2. This table also highlights the CERQual profile. The three key conceptual categories identified by the team and are described in this section. The remaining 13 conceptual categories are described in more detail in Supplementary File 2. The concepts within each conceptual category are presented in Supplementary File 3.

**Managing pain**

Pain was seen as a major obstacle to return to work. [44 52 58] A plethora of strategies to manage it were described [41 45 52 54 57 58] including use of sick leave.[11 42] However, the strain of living with chronic pain meant fatigue also became a problem and low energy levels prevented work return.[58] The impact of pain on performance [63] and ability to attend and travel to work[48] along with the fear of pain exacerbation[31 45] were also problematic.

**Managing work relationships**

Interpersonal conflict and mutual mistrust can arise between people with pain and their employers and colleagues[37 61] and if relationships with supervisors are perceived as poor then this is demotivating in relation to work return.[38] Employers with few employees expressed reservations about how far to push an employee for fear of upsetting them and causing them to be off sick for longer than necessary.[60] Managers in a public sector study appeared to be walking a fine line between supporting employees, making sure colleagues were not adversely affected, and that services were delivered.[63] Asking for help was perceived as frustrating by people in pain, and incurred feelings of inadequacy and negativity.[25] Some struggled in their interaction with employers and tended to be passive, not believing their views were listened to, or valued, which led to difficulties in sustaining work return.[11] Unsympathetic employer attitude and a lack of understanding of the person's experience of pain were seen as a major obstacles to work return[12 34 45 54 59] but those

For peer review only

employers with personal experience of pain were perceived as more sympathetic and empathic.[30 62]

Team management responsibilities of regulating tension between colleagues was perceived as challenging when work restrictions for those with pain caused unequal work distribution leading to a sense of injustice.[40]

**Making workplace adjustments**

An economic climate of austerity was perceived as an obstacle to work due to reduced job availability and a competitive job market.[12] Reorganisations and rationalisation in the workplace meant jobs had changed and become more demanding and potentially difficult to adapt for people with a pain condition.[48] In this situation, age was also seen as influential with some feeling they were too old to retrain for a different kind of job.[12 59]

The type of job influenced work return decisions with physical work being perceived as more challenging with pain[40 59] and more highly skilled work providing greater scope for flexibility and adaptation.[28]

People with chronic pain often felt they were not consulted or involved in the decision making about workplace redeployment or adjustment and when desired modifications were not possible they could not return to work. [28 30 34 59] Managers' attitudes and efforts, [62] combined with effective routine methods of regular communication of changes made to colleagues[53] were seen as ways of improving the success of workplace adjustments. Managers did not always have the resources or know what options would be available for making these adjustments and saw the planning of these accommodations as an additional demand on their time.[62] Managers also felt that information about work restrictions from occupational health were not always realistic in the work setting and therefore difficult to implement.[40]

A number of workplace adjustments were felt to be helpful including flexible hours or a reduction in hours but were not always forthcoming.[28 34 48] The possibility of a gradual return

For peer review only

to work[35] working from home or participating in a job sharing programme[34] were also seen as helpful by people in chronic pain. Changes to the job itself including physical adjustments and a reduction in job demands were not always feasible, for example in a nursing[34], nursing assistant role[48] or a preschool teaching role.[47]

For peer review only

**Table 2 Conceptual categories, description of category, supporting studies and CERQual assessment**

| | Conceptual category - summary of review finding | Quotations from primary studies to illustrate conceptual category | Supporting studies | Adequacy (number of concepts – see list of concepts in supplement-ary file 3) | Coherence (number of supporting studies) | Methodological limitations (see CASP scores in supplemen-tary file 1) | Relevance | Overall CERQual assessment of confidence in the evidence | Explanation of CERQual assessment |
|---|---|---|---|---|---|---|---|---|---|
| 1 | **Managing pain** - the impact of pain on return to work and how it can be managed | 'Chronic pain itself was the underlying barrier from which most other barriers to work stem. Overall, very few patients reported any attempts to plan for the future, primarily due to the unpredictable nature of the pain condition and physical mobility problems associated: 'I have no objections at all to go back to work. But, I thought about this. I don't know what I could do. I can't sit for very long. I can't stand for very long. Erm, in discomfort 99% of the time.' [Male, 56]'[12] | 11 12 27 28 31 35 38 39 41 42 44 45 48 50 52 54 56-58 63 | Richly described (49) | Fit between underlying data and review finding is very clear (20) | All CASP scores over 20 | Sweden 9<br>UK 5<br>Canada 2<br>Norway 1<br>Switzerland 1<br>USA 1<br>Australia 1<br><br>Three studies partially relevant as only included women not men. | High confidence | Graded as high in relation to adequacy, coherence, methodological limitations and relevance. Employee and employer studies support this finding. |
| 2 | **Managing work relationships** - impact of chronic pain on relationships with employers, line managers and colleagues | 'The existence of interpersonal conflict with colleagues or managers was mentioned as a barrier, as was mutual mistrust. For managers, overwork, role conflict between production | 11 12 25 28 30 34-40 43-45 48 49 53-55 59-63 | Richly described (51) | Fit between underlying data and review finding is very clear (25) | All CASP scores over 20 | Sweden 10<br>Denmark 1<br>UK 5<br>Canada 5<br>France 2<br>Norway 1<br>South Africa 1 | High confidence | Graded as high in relation to adequacy, coherence, methodological limitations and relevance. Employee and |



For peer review only

| | | | | | | | | | |
|---|---|---|---|---|---|---|---|---|---|
| | and how this is managed | targets and occupational health and a lack of hierarchic support were possible barriers. With colleagues, overwork and scepticism about medical problems could induce hostility and rejection. For workers with LBP, the feeling of being judged and having to justify absence, pain and limitations was perceived as a barrier.'[37] | | | | | | | employer studies support this finding. |
| 3 | **Making workplace adjustments** - the scope and process for making changes to the job, work conditions or environment | 'Our data suggest that the ability of participants to remain in employment was in part influenced by the nature of their work (whether or not adaptations could be made to enable employees to continue in post despite their symptoms) and in part due to patients' confidence and ability to negotiate adaptations with their employers (significant others often described themselves as being an important source of support for the patient in this context).'[28]<br><br>'Accommodation | 11 12 26 28-30 34-37 40 42 47 48 52 53 58-60 62 | Richly described (55) | Fit between underlying data and review finding is very clear (20) | All CASP scores over 20 | Denmark 2<br>Sweden 6<br>UK 3<br>Canada 4<br>Norway 2<br>France 2<br>South Africa 1 | High confidence | Graded as high in relation to adequacy, coherence, methodological limitations and relevance. Employee and employer studies support this finding. |

For peer review only

| | | | | | | | | | |
|---|---|---|---|---|---|---|---|---|---|
| | | management is perceived as a considerable addition to supervisors' regular duties, for which they feel ill-prepared, even where guidance is provided by others with the requisite expertise.'[62] | | | | | | | |
| **4** | **Autonomy** - the individual's ability to have control or agency in relation to their pain and their work situation | 'Several respondents emphasized the importance of their possibilities to control what to do and when to do it and considered flexible working hours as a prerequisite for their return to work'[36] | 11 12 26 28-30 35 36 38 39 41 45 | Well described (12) | Fit between underlying data and review finding is clear (12) | All CASP scores over 20 | Sweden 5<br>UK 4<br>Denmark 2<br>Canada 1 | Moderate confidence | Graded as moderate as well described and relevant across four cultures. |
| **5** | **Self-belief/ self-efficacy** - the individual's outlook about their ability to handle work and manage their pain | 'Self-efficacy statements pertaining to more complex work-related functions were subdivided into one of three categories based on the thematic content analysis: the ability to meet job demands, the ability to obtain help from others, and the ability to cope with pain'[57] | 11 26 28 36 38 45 46 49 54 57 61 | Richly described (18) | Fit between underlying data and review finding is very clear (11) | All CASP scores over 20 | Sweden 5<br>Denmark 1<br>Norway 2<br>UK 2<br>USA 1 | High confidence | Graded as high in relation to adequacy, coherence, methodological limitations and relevance |
| **6** | **Being believed** - people struggling with not being believed, trusted or perceived as | 'Employees typically discussed issues around being believed and trusted when they were ill. Managers, on the other hand, were more likely to talk about employees | 26 29 37 39 50 54 57 59 63 | Well described (13) | Fit between underlying data and review finding is clear (8) | All CASP scores over 20 | Denmark 2<br>France 1<br>Sweden 2<br>UK 2<br>South Africa 1 | Moderate confidence | Graded as moderate as well described and relevant across five cultures. Employee and employer studies |

For peer review only

| | | | | | | | | | |
|---|---|---|---|---|---|---|---|---|---|
| | legitimate | taking absence that was not legitimate, for example; ''People just don't turn up, you know. They phone in sick or er... The attitude is they, you know, 'why should I bother?' sort of thing. You get a lot of that.'' [Female manager]' [63] | | | | | | | support this finding. |
| **7** | **Impact of and on the family** - the effects of chronic pain on family members and vice versa | 'Lots of patients with LBP are looked after by their family members who relieve them of physical activities. Many patients receive more attention and are encouraged to take rest. The positive feeling of being supported is counteracted by the negative feeling of uselessness, associated with being off work'[56] | 27 28 47 48 50 51 54 56 | Well described (13) | Fit between underlying data and review finding is clear (8) | All CASP scores over 20 | Australia 1<br>UK 3<br>Sweden 2<br>Switzerland 1<br>Norway 1 | Moderate confidence | Graded as moderate as well described and relevant across five cultures |
| **8** | **Not being understood** - this is in the context of relationships with health professionals | 'Participants felt that physicians did not understand their clients' work environment, such as what functional demands were necessary for them to complete their tasks as well as the psychosocial stressors that could cause their back pathology to become chronic'. [59] | 33 47 52 56 59 60 | Adequately described (9) | Some inconsistency in fit (6) | All CASP scores over 20 | Canada 2<br>Norway 2<br>Switzerland 1<br>South Africa 1 | Low confidence | Graded as low as some concerns about coherence and relevant across four cultures. Employee and employer studies support this finding. |
| **9** | **Finance and** | 'Some patients who were | 12 34 41 43 48 49 56 | Well | Fit between | All CASP | Canada 1 | Moderate | Graded as |

For peer review only

| | | | | | | | | | |
|---|---|---|---|---|---|---|---|---|---|
| | **benefits** - financial difficulties and the economic insecurity of moving from welfare benefits back into work | unemployed on grounds of ill health had serious concerns about their financial future. They complained of sleep disorders and mental problems: "Without medication I can't sleep. I don't know what is going to happen"' [56]<br><br>'It must be possible to find transition solutions when trying to get back to work, solutions that make us feel economically secure. Otherwise, who would dare to try?'[49] | 57 | described (10) | underlying data and review finding is clear (8) | scores over 20 | UK 1<br>USA 1<br>Switzerland 1<br>Sweden 3<br>Norway 1 | confidence | moderate as well described and relevant across six cultures |
| **10** | **Health and illness and pain representations impact on return to work** - the way people think about their pain and the mental representations they form in relation to beliefs about its cause and their perception of its impact on their lives | 'Beliefs about causality. All claimants reported work as the initial cause of their back pain condition, and most also perceived previous work/certain types of work (manual/heavy/repetitive) as a 'trigger' for subsequent episodes and therefore not conducive to return to work'. [50]<br><br>'Both patients and significant others in the non-working sample were resigned to the permanent effects of the patient's | 26-29 31 32 39 44 45 50 54 56 60 | Richly described (22) | Fit between underlying data and review finding is very clear (13) | All CASP scores over 20 | Denmark 2<br>Australia 1<br>UK 3<br>Canada 3<br>Sweden 3<br>Switzerland 1 | High confidence | Graded as high in relation to adequacy, coherence, methodological limitations and relevance. Employee and employer studies support this finding. |

For peer review only

| | | | | | | | | | |
|---|---|---|---|---|---|---|---|---|---|
| | | back problem on their employment status and were thus more likely to consider the patient as 'disabled', a role which might become self-fulfilling'. [28] | | | | | | | |
| 11 | **Meaning of work** - the meaning of work for an individual linked with motivation | 'Work was viewed by most patients as a source of financial security and a means of independence. However, the financial aspect of work did not seem to motivate all patients. A separate subgroup placed greater focus on health status and pain reduction strategies. This was a more prevalent attitude amongst those who had had longer durations of sick leave. For example, a patient that had been unemployed for more than one year stated: 'Money wouldn't be a motivator for me, I'd have to be well enough to return to work – that would be the motivator' [45].<br><br>'Participants who were placed in jobs that had no meaning to them caused them to become | [36 38 39 42-46 48 52 55 59-63] | Richly described (26) | Fit between underlying data and review finding is very clear (14) | All CASP scores over 20 | Sweden 7<br>UK 2<br>Norway 2<br>Canada 2<br>South Africa 1 | High confidence | Graded as high in relation to relevance, coherence, adequacy & methodological limitations. Employee and employer studies support this finding. |

For peer review only

| | | | | | | | | | |
|---|---|---|---|---|---|---|---|---|---|
| | | frustrated. When these meaningless tasks were coupled with relapses of back pain, there would be a downward spiral into depression and demotivation amongst the participants'. [59] | | | | | | | |
| 12 | **Mismatch between employee and employer expectations of return to work** | Meeting job demands typically referred to producing a certain quantity of work (e.g. 'I need to be at full capacity'), quality of work (e.g. 'I may not do a good job'), speed of work (e.g. 'I won't be able to keep up'), or fulfilling a particular role at work (e.g. 'I need to be able to respond to an emergency'). [57] | 12 36 38 45 46 49 53 57 58 62 | Well described (11) | Fit between underlying data and review finding is clear (10) | All CASP scores over 20 | Sweden 4<br>UK 2<br>Norway 2<br>USA 1<br>Canada 1 | Moderate confidence | Graded as moderate as well described and relevant across five cultures. Employee and employer studies support this finding. |
| 13 | **Social isolation as a consequence of chronic pain** - leading to a lack of support to return to work | Paid work – the pain sufferer's struggle for social capital.<br>The informants were concerned about how the unpredictability of the pain broke into their daily lives and social contact with others, and challenged their normal way of dealing with everyday problems.[52] | 27 29 48 52 54 | Adequately described (6) | Fit between underlying data and review finding is clear (5) | All CASP scores over 20 | Australia 1<br>Denmark 1<br>Sweden 2<br>Norway 1 | Low confidence | Graded as low as adequately described and relevant across four cultures |
| 14 | **Influence of return to work support and** | 'Support. The informants felt that the rehabilitation programme was the right | 25 26 29 31 33 35 36 39 45 48 54 56 57 | Well described (19) | Some inconsistency in fit | All CASP scores over 20 | Denmark 3<br>Canada 3<br>Sweden 4 | Moderate confidence | Graded as moderate as well described and |

For peer review only

| | | | | | | | | | |
|---|---|---|---|---|---|---|---|---|---|
| | **rehabilitation** | place to come to when living with long-term pain. The team was described as empathetic and knowledgeable: when the informants told about their difficulties they felt understood for the first time. The informants also got support from each other, and a good feeling of fellowship developed; on the other hand, some also described how they were negatively affected by other participants who were depressed'. [54] | | | (13) | | Switzerland 1<br>USA 1<br>UK 1 | | relevant across six cultures. |
| 15 | **System factors (healthcare, social security and workplace systems)** - how policies and procedures in these three systems impact on people with chronic pain | 'The way "systems" (dys)function delay return to work. When a worker becomes injured, they enter into complex relationships with the compensation system, unions, workplace, and health care system. How these systems interact with one other and with the injured worker can affect the RTW process.' [60]<br><br>'Within the healthcare system, participants talked of their frustrations with long waiting times, such as waiting for specialist | 12 30 34 35 37 39 49 54 55 59 60 63 | Richly described (25) | Fit between underlying data and review finding is very clear (12) | All CASP scores over 20 | UK 3<br>Canada 4<br>Sweden 2<br>Norway 1<br>South Africa 1<br>France 1 | High confidence | Graded as high in relation to relevance, coherence, adequacy & methodological limitations. Employee and employer studies support this finding. |

For peer review only

| | | | | | | | | | |
|---|---|---|---|---|---|---|---|---|---|
| | | appointments, diagnostic tests, treatments and entry into programs.' [55]<br><br>De-motivating economic arrangements for pensioners were also mentioned as a barrier. Some pointed to the fact that income under re-education was so low that the effort was not worthwhile. In addition, several had experienced that taking a small part time job when receiving pension would reduce the benefit so that nothing was gained economically. Finally, trying out for new jobs arranged by the job centre put them in an economically uncertain position and made them afraid of losing their disability benefit all together. More appropriate transitional arrangements were asked for. [49] | | | | | | | |
| 16 | **Societal expectations** - expectations of family, friends and wider society that | 'Experiences of societal expectations of participation in work. In the societal discourse of work participation, inclusion in society was | 47 36 46 59 | Adequately described (3) | Some inconsistency in fit (4) | All CASP scores over 20 | Sweden 1 Norway 2 South Africa 1 | Low | Graded as low as adequately described and relevant across three cultures. |

For peer review only

| | | | | | | | | | |
|---|---|---|---|---|---|---|---|---|---|
| | everyone should work resulting in judgement and discrimination against those who don't work | connected to employment.' [47]<br><br>'Some of the participants viewed their family and society as being judgemental, unsupportive and discriminatory, whereas others felt that they could not have rehabilitated themselves without the support of society.' [59] | | | | | | | |

N.B. No more than half female only studies supported any of the review findings.

For peer review only

### Line of argument

A line of argument was constructed by examining how the conceptual categories relate to each other. A flow diagram/ conceptual model was then developed, see figure 2.

(**insert Figure 2 here**)

The conceptual model of return to work (figure 2) is now explained, going anti-clockwise from 1) to 7).

1) The underpinning foundation lies in the cultural expectation within society that people should work and contribute to the economy. Societal expectations are manifested within institutions, families and the media.
2) Societal and family expectations influence the individual's sense of self and what work means to each person. Meaning can relate to financial remuneration, rewards or survival, meeting of social, cognitive and achievement needs or purpose in life. The individual's level of self-belief and autonomy will both play a part in how much agency and control can be exerted over pain and the work situation.
3) The way someone thinks about their pain and the mental representation they create will also influence their behaviour and the possibility of returning to work. People's perceptions of whether pain is a long-term disability could influence whether they feel able to work and thus their return to work decisions, whereas someone who has accepted the pain as part of their life and adapts may be more likely to consider return to work.
4) Some studies in the review evaluated pre-return to work support or rehabilitation programmes and not being understood by health professionals was cited as an obstacle. In the same way, not being believed or being judged by people in the workplace was also perceived to make return to work challenging.
5) The three key tenets of return to work are managing pain, managing work relationships and making workplace adjustments. Tension exists between these

For peer review only

three facets and they can be influenced by a mismatch between the individual and the employer expectations, difficulties asking for help and system factors in the workplace, health and social security systems.

6) Individuals must negotiate a wide range of obstacles and navigate change.
7) This could result in a downward spiral (and not working) at one end of a continuum through to an upward spiral of mastering return to work despite pain.

## Discussion

In this meta-ethnography we identified obstacles to return to work for people with chronic pain centred around three key conceptual categories; managing pain, managing work relationships in the workplace and making workplace adjustments. The balancing of these three inter-related categories and the way they are influenced by other factors is central to negotiating a successful return to work. The scope for managing pain and making adjustments in the workplace is influenced by the quality of the relationship an individual has with their employer and/ or line manager and what is feasible within a particular work setting.

The concepts of health and pain representations and the role of significant others and their thinking about pain and return to work do not appear to be highlighted by previous reviews. Another neglected area is the influence of pre-return to work support or rehabilitation. The employer perspective is missing in earlier reviews which have focused on the experience of people with chronic pain. Only five of the included studies were conducted with employers so there is still limited research with this group despite people with pain emphasising the importance of employer attitudes and knowledge in the return to work process[53].

Some studies that were included in the review appear to suggest that those people with chronic pain who manage to stay in work have different characteristics to those who are unable to do so. This is seen in part to be connected with their cognitive appraisal of their pain and whether they are able to adapt.[31 45] It has been proposed that in those who do not return to work their pain representation of 'abnormal pain' becomes crystallised with their

For peer review only

goal of pain elimination firmly intact whereas those who returned to work began to perceive pain as ‘ the new normal’ and something they learn to live with.[31] Eden *et al.* (2007)[36] described three different adaptation patterns, the go-getter, realist and indifferent. They proposed the pessimistic and passive outlook of the latter type meant work return was less likely. Passivity in the interaction with stakeholders like the employer was found to be linked with reduced drive to return to work.[11] Angel *et al*. (2012) [26] and Dionne *et al*. (2013) [35] also found passivity in relation to pain was not helpful when addressing workplace obstacles.

The provision of professional individualised support and coaching in the workplace was seen to be valuable in the work return process [39] and this concept supports the idea of developing work based interventions to help people with chronic pain return to work.

When comparing findings with previous reviews that have highlighted obstacles to return to work, similarities include fears of not being able to fulfil employer expectations, not being believed by colleagues and financial concerns.[4] Worries for the future including financial and job security were also uncovered by MacNeela *et al*. (2015).[5] Strain on the family relationships including those with partners and children[4] and gender differences regarding role as carer or breadwinner were revealed.[5] Unsatisfying relationships with health professionals where people felt they were not being listened to and frustrations with limitations of medical treatment were another common feature.[5] Social withdrawal as a result of pain was highlighted in both of these reviews.[4 5] A struggle for legitimacy with colleagues and stigma in the workplace was highlighted by Toye[7] and Froud[4]. This review also drew attention to the system not supporting return to work due to a lack of dialogue between employers, occupational health and the health system to facilitate a gradual return with appropriate adjustments.

In relation to methodology, focus on studies that dealt with obstacles to return to work was achieved through carefully reading and re-reading abstracts and then full texts.

For peer review only

The collaborative team approach to conceptual analysis increased the rigour of the review.[13] Independently drawing flow diagrams to illustrate the conceptual model and then coming together to amalgamate these through discussion and debate, combined with checking all concepts had been included, ensured this process was thorough.

The CERQual assessments indicated there was a high level of confidence in the findings for managing pain, managing work relationships, managing the workplace, self-belief, health and illness representations, the meaning of work and system factors. Although we have used CERQual, we found we agreed with many comments on its use by Toye[64], namely that for relevance, studies rated as partially or indirectly relevant could also contain helpful concepts. They suggest 'gravitational pull' of an idea may be important. They argue providing clear information about concepts is critical, and we have provided this in Supplementary Files 2 and 3. They also note for adequacy "The power of concepts to make us think, however, is not based on quantity of data included". We agree when looking at coherence that inconsistent findings do not necessarily call the findings into question. It may be one study has developed an insight not considered in other studies. No tool can guarantee confidence in findings, and authors still need to carefully consider issues rigour.

In addition, our view is that CERQual combines qualitative and quantitative concepts. Some of the quantitative concepts used have very specific meanings within inferential statistics, which could cause confusion and potentially over-reaching conclusions in some reviews.

**Implications**

This review identifies obstacles faced by people with chronic pain in returning to work after a period of sick leave or unemployment and can be used to inform the development of a return to work intervention. The focus of such intervention should be working collaboratively with the person who has chronic pain and the employer to explore ways of addressing managing pain, managing work relationships and making workplace adjustments.

**Limitations**

For peer review only

It is apparent that more research is required from the employer perspective. The five studies included in the review were from the perspective of employers working in car making, university hospitals, home care provision for disabled people in France[37], public hospitals in France[40] and NHS Trust and local authority in Wales.[63] The Canadian study that included small and large employers did not specify the nature of the industry in which they were engaged. [60]

## Conclusions

The navigation of obstacles to return to work for people with chronic pain and their employers entails balancing the needs of the person with chronic pain, colleagues and the employing organisation. The influence of health and pain representations the person formulates has not been emphasised in previous reviews. Managing pain, managing relationships in the workplace and making adjustments are central to achieving a successful return to work and these can be hard work for the person with chronic pain.

For peer review only

**Acknowledgments**

We would like to thank Samantha Johnson, Senior Medical Librarian at University of Warwick for her help with initial literature search and helpful guidance during the updated search process.

This research is part of the RISE (Return to work with Individualised Supported Employment) feasibility study supported by the Arthritis Research UK charity (project number 9401). We would like to thank Debs Smith, patient and public involvement representative, who participated in developing the research protocol as a co-applicant.

**Author's contributions**

KS, RF and MU made a substantial contribution to the design of the study. MG and KS were responsible for acquisition and MG, KS and JOB were responsible for analysis and interpretation of the data. MG drafted the first, subsequent and final versions and KS, JOB, RF and MU revised all versions for important intellectual content and approved the final version. All authors agree to be accountable for the accuracy and integrity of the work.

**Competing interests**

MU was Chair of the NICE accreditation advisory committee until March 2017 for which he received a fee. He is chief investigator or co-investigator on multiple previous and current research grants from the UK National Institute for Health Research, Arthritis Research UK and is a co-investigator on grants funded by Arthritis Australia and Australian NHMRC. He has received travel expenses for speaking at conferences from the professional organisations hosting the conferences. He has received travel and subsistence to attend meetings by the EU Joint Research Centre. He is an editor of the NIHR journal series for which he receives a fee. He has published multiple papers on chronic pain some of which are referenced in this paper.

For peer review only

RF and MU are directors and shareholders of Clinvivo Ltd that provides electronic data collection for health services research. RF and MU are part of an academic partnership with Serco Ltd related to return to work initiatives.

KS has published multiple papers on pain and meta-ethnography, some of which are referenced in this paper.

## References


1. Blyth FM, March LM, Brnabic AJM, et al. Chronic pain in Australia: a prevalence study. Pain 2001;**89**(2):127-34.
2. Goldberg DS, McGee SJ. Pain as a global public health priority. BMC Public Health 2011;**11**:770.
3. Toye F SK, Hannink E, Barker K. A mega-ethnography of eleven qualitative evidence syntheses exploring the experience of living with chronic malignant pain. BMC Medical Research Methodology 2017;**17**:116-.
4. Froud R, Patterson S, Eldridge S, et al. A systematic review and meta-synthesis of the impact of low back pain on people's lives. BMC Musculoskelet Disord 2014;**15**:50.
5. MacNeela P, Doyle C, O'Gorman D, et al. Experiences of chronic low back pain: a meta-ethnography of qualitative research. Health Psychology Review 2015;**9**(1):63-82.
6. Johannes CB, Le TK, Zhou X, et al. The Prevalence of Chronic Pain in United States Adults: Results of an Internet-Based Survey. The Journal of Pain;**11**(11):1230-39.
7. Toye F, Seers K, Allcock N, et al. A synthesis of qualitative research exploring the barriers to staying in work with chronic musculoskeletal pain. Disabil Rehabil 2016;**38**(6):566-72.
8. de Vries HJ, Brouwer S, Groothoff JW, et al. Staying at work with chronic nonspecific musculoskeletal pain: a qualitative study of workers' experiences. BMC Musculoskelet Disord 2011;**12**:126.
9. Patel AS, Farquharson R, Carroll D, et al. The Impact and Burden of Chronic Pain in the Workplace: A Qualitative Systematic Review. Pain pract 2012;**12**(7):578-89.
10. Henriksson CM, Liedberg GM, Gerdle B. Women with fibromyalgia: Work and rehabilitation. Disability and Rehabilitation 2005;**27**(12):685-95.
11. Ahlstrom L, Dellve L, Hagberg M, et al. Women with Neck Pain on Long-Term Sick Leave. Approaches Used in the Return to Work Process: A Qualitative Study. J Occup Rehabil Journal of Occupational Rehabilitation 2017;**27**(1):92-105.
12. Patel S, Greasley K, Watson PJ. Barriers to rehabilitation and return to work for unemployed chronic pain patients: a qualitative study. Eur J Pain 2007;**11**(8):831-40.
13. Toye F, Seers K, Allcock N, et al. Meta-ethnography 25 years on: challenges and insights for synthesising a large number of qualitative studies. BMC Medical Research Methodology 2014b;**14**:80.
14. Noblit GW, Hare RD. *Meta-Ethnography : synthesizing qualitative studies*. Newbury Park, Calif. : Sage, 1988.
15. CASP. Critical appraisal skills programme qualitative assessment tool 2018 [Available from: http://media.wix.com/ugd/dded87_29c5b002d99342f788c6ac670e49f274.pdf.
16. Lewin S, Booth A, Glenton C, et al. Applying GRADE-CERQual to qualitative evidence synthesis findings: introduction to the series. Implement Sci 2018;**13**(Suppl 1):2.
17. Lewin S, Bohren M, Rashidian A, et al. Applying GRADE-CERQual to qualitative evidence synthesis findings-paper 2: how to make an overall CERQual assessment of confidence and create a Summary of Qualitative Findings table. Implement Sci 2018;**13**(Suppl 1):10.


For peer review only

18. Glenton C, Carlsen B, Lewin S, et al. Applying GRADE-CERQual to qualitative evidence synthesis findings-paper 5: how to assess adequacy of data. Implement Sci 2018;**13**(Suppl 1):14.
19. Colvin CJ, Garside R, Wainwright M, et al. Applying GRADE-CERQual to qualitative evidence synthesis findings-paper 4: how to assess coherence. Implement Sci 2018;**13**(Suppl 1):13.
20. Munthe-Kaas H, Bohren MA, Glenton C, et al. Applying GRADE-CERQual to qualitative evidence synthesis findings-paper 3: how to assess methodological limitations. Implement Sci 2018;**13**(Suppl 1):9.
21. Noyes J, Booth A, Lewin S, et al. Applying GRADE-CERQual to qualitative evidence synthesis findings-paper 6: how to assess relevance of the data. Implement Sci 2018;**13**(Suppl 1):4.
22. Schutz A. Concept and theory formation in the social sciences. Journal of Philosophy 1954;**51**(9):270-73.
23. NVivo 11 [program]: QSR International Pty Ltd. , 2012.
24. Toye F, Seers K, Allcock N, et al. 'Trying to pin down jelly' - exploring intuitive processes in quality assessment for meta-ethnography. BMC Medical Research Methodology 2013b;**13**:46.
25. Andersen LN, Kohberg M, Herborg LG, et al. Here we're all in the same boat - a qualitative study of group based rehabilitation for sick-listed citizens with chronic pain. SJOP Scandinavian Journal of Psychology 2014;**55**(4):333-42.
26. Angel S, Jensen LD, Gonge BK, et al. Patients' interpretations of a counselling intervention for low back pain: a narrative analysis. Int J Nurs Stud 2012;**49**(7):784-92.
27. Ashby S, Richards K, James C. The effect of fear of movement on the lives of people with chronic low back pain...including commentary by Carleton RN, Poulain C, Meyer K, and Glombiewski JA. International Journal of Therapy & Rehabilitation 2010;**17**(5):232-43.
28. Brooks J, McCluskey S, King N, et al. Illness perceptions in the context of differing work participation outcomes: exploring the influence of significant others in persistent back pain. BMC Musculoskelet Disord 2013;**14**.
29. Buus N, Jensen LD, Maribo T, et al. Low back pain patients' beliefs about effective/ineffective constituents of a counseling intervention: a follow-up interview study. Disabil Rehabil 2015;**37**(11):936-41.
30. Coole C, Watson PJ, Drummond A. Low back pain patients' experiences of work modifications; a qualitative study. BMC Musculoskelet Disord 2010;**11**:277.
31. Coutu MF, Baril R, Durand MJ, et al. Transforming the meaning of pain: an important step for the return to work. Work (Reading, Mass) 2010;**35**(2):209-19.
32. Coutu MF, Baril R, Durand MJ, et al. Health and illness representations of workers with a musculoskeletal disorder-related work disability during work rehabilitation: a qualitative study. J Occup Rehabil 2011;**21**(4):591-600.
33. Coutu MF, Baril R, Durand MJ, et al. Clinician-patient agreement about the work disability problem of patients having persistent pain: why it matters. J Occup Rehabil 2013;**23**(1):82-92.
34. Crooks VA. Women's experiences of developing musculoskeletal diseases: Employment challenges and policy recommendations. Disability and Rehabilitation 2007;**29**(14):1107-16.
35. Dionne C, Bourbonnais R, Frémont P, et al. Obstacles to and Facilitators of Return to Work After Work-Disabling Back Pain: The Workers' Perspective. J Occup Rehabil 2013;**23**(2):280-89.
36. Edén L, Andersson HI, Ejlertsson G, et al. Characteristics of disability pensioners returning to work: an interview study among individuals with musculoskeletal disorders. Disabil Rehabil 2007;**29**(22):1720-26.
37. Fassier J-B, Durand M-J, Caillard J-F, et al. Results of a feasibility study: barriers and facilitators in implementing the Sherbrooke model in France. Scand J Work Environ Health 2015;**41**(3):223-33.
38. Gard G, Sandberg AC. Motivating factors for return to work. Physiother Res Int 1998;**3**(2):100-8.
39. Glavare M, Löfgren M, Schult M-L. Between unemployment and employment: Experience of unemployed long-term pain sufferers. Work 2012;**43**(4):475-85.

For peer review only

40. Grataloup M, Massardier-Pilonchéry A, Bergeret A, et al. Job Restrictions for Healthcare Workers with Musculoskeletal Disorders: Consequences from the Superior's Viewpoint. J Occup Rehabil 2016;**26**(3):245-52.
41. Hansson M, Boström C, Harms-Ringdahl K. Living with spine-related pain in a changing society—a qualitative study. Disability and Rehabilitation: An International, Multidisciplinary Journal 2001;**23**(7):286-95.
42. Hansson M, Bostrom C, Harms-Ringdahl K. Sickness absence and sickness attendance--what people with neck or back pain think. Soc Sci Med 2006;**62**(9):2183-95.
43. Johansson EE, Hamberg K, Lindgren G, et al. 'How could I even think of a job?' - Ambiguities in working life in a group of female patients with undefined musculoskeletal pain. Scand J Prim Health Care 1997;**15**(4):169-74.
44. Juuso P, Skär L, Sundin K, et al. The Workplace Experiences of Women with Fibromyalgia. MSC Musculoskeletal Care 2016;**14**(2):69-76.
45. Kalsi P, Turkistani W, Sykes C, et al. "Work is a beautiful thing." Exploring attitudes towards employment in chronic pain (CP) patients attending a pain management programme (PMP). Journal of Vocational Rehabilitation 2016;**44**(1):97-107.
46. Kvam L, Eide AH, Vik K. Understanding experiences of participation among men and women with chronic musculoskeletal pain in vocational rehabilitation. Work 2013;**45**(2):161-74.
47. Kvam L, Vik K. Discourses of participation in work among men and women in vocational rehabilitation. Journal of Occupational Science 2015;**22**(3):345-57.
48. Liedberg GM, Henriksson CM. Factors of importance for work disability in women with fibromyalgia: An interview study. Arthritis Care and Research 2002;**47**(3):266-74.
49. Magnussen L, Nilsen S, Råheim M. Barriers against returning to work--as perceived by disability pensioners with back pain: a focus group based qualitative study. Disabil Rehabil 2007;**29**(3):191-97.
50. McCluskey S, Brooks J, King N, et al. The influence of 'significant others' on persistent back pain and work participation: a qualitative exploration of illness perceptions. BMC Musculoskelet Disord 2011;**12**:236.
51. McCluskey S, Brooks J, King N, et al. Are the treatment expectations of 'significant others' psychosocial obstacles to work participation for those with persistent low back pain? Work 2014;**48**(3):391-8.
52. Nilsen G, Anderssen N. Struggling for a normal life: Work as an individual self-care management strategy among persons living with non-malignant chronic pain. Work 2014;**49**(1):123-32.
53. Nordqvist C, Holmqvist C, Alexanderson K. Views of Laypersons on the Role Employers Play in Return to Work When Sick-Listed. J Occup Rehabil 2003;**13**(1):11-20.
54. Rydstad M, Schult M, Löfgren M. Whiplash patients' experience of a multimodal rehabilitation programme and its usefulness one year later. Disabil Rehabil 2010;**32**(22):1810-18.
55. Saunders SL, MacEachen E, Nedelec B. Understanding and building upon effort to return to work for people with long-term disability and job loss. Work 2015;**52**(1):103-14.
56. Scheermesser M, Bachmann S, Schamann A, et al. A qualitative study on the role of cultural background in patients' perspectives on rehabilitation. BMC Musculoskelet Disord 2012;**13**:5.
57. Shaw WS, Huang YH. Concerns and expectations about returning to work with low back pain: identifying themes from focus groups and semi-structured interviews. Disabil Rehabil 2005;**27**(21):1269-81.
58. Sjöström R, Melin-Johansson C, Asplund R, et al. Barriers to and possibilities of returning to work after a multidisciplinary rehabilitation programme. A qualitative interview study. Work 2011;**39**(3):243-50.
59. Soeker MS, Wegner L, Pretorius B. I'm going back to work: Back injured clients' perceptions and experiences of their worker roles. Work 2008;**30**(2):161-70.

For peer review only

60. Soklaridis S, Ammendolia C, Cassidy D. Looking upstream to understand low back pain and return to work: Psychosocial factors as the product of system issues. Soc Sci Med 2010;**71**(9):1557-66.
61. Svensson T, Mussener U, Alexanderson K. Sickness absence, social relations, and self-esteem: a qualitative study of the importance of relationships with family, workmates, and friends among persons initially long-term sickness absent due to back diagnoses. Work 2010;**37**(2):187-97.
62. Williams-Whitt K, Kristman V, Shaw WS, et al. A Model of Supervisor Decision-Making in the Accommodation of Workers with Low Back Pain. J Occup Rehabil 2016;**26**(3):366-81.
63. Wynne-Jones G, Buck R, Porteous C, et al. What happens to work if you're unwell? Beliefs and attitudes of managers and employees with musculoskeletal pain in a public sector setting. J Occup Rehabil 2011;**21**(1):31-42.
64. Toye F Seers K Barker K (2018) A meta-ethnography of healthcare professionals' experience of treating adults with chronic musculoskeletal pain to improve the experience and quality of healthcare. NIHR Health Services and Delivery Research. **6**(17) APRIL 2018 ISSN 2050-4349 DOI 10.3310/hsdr06170

**Figure 1 legend:**

**Title: Figure 1 - Flow chart illustrating search outcome**

Figure 1 legend: This flow chart illustrates the search outcome

**Figure 2 legend:**

**Title: Figure 2 – Conceptual model – The work of return to work**

**Figure 2 legend:** This conceptual model of return to work is explained in the text, going anti-clockwise from 1) to 7).

**Data Sharing Statement**

We have tried to include all relevant data for the qualitative systematic review in supplementary files. Any other reasonable requests will be considered on a case by case basis by Mary Grant (lead author) email: M.Grant.2@warwick.ac.uk=

For peer review only

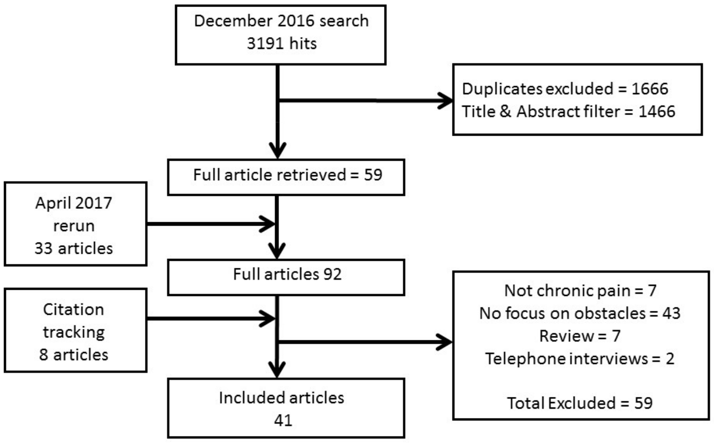


Figure 1 - Flow chart illustrating search outcome

108x60mm (300 x 300 DPI)

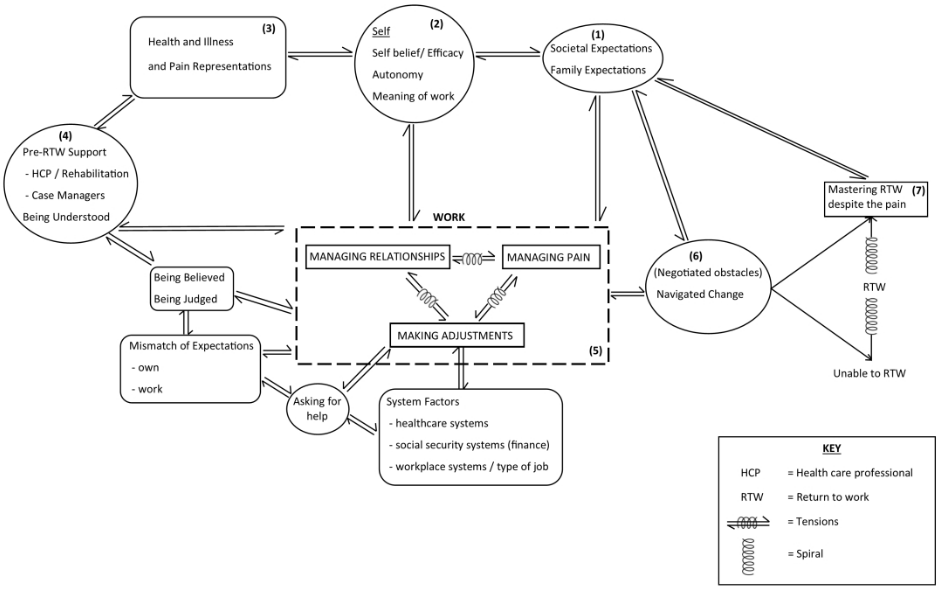


Figure 2 – Conceptual model – The work of return to work
This conceptual model of return to work is explained in the text, going anti-clockwise from 1) to 7).

297x209mm (300 x 300 DPI)

**Supplementary File 1 – Critical Appraisal Skills (CASP) scores – yes =3, can't tell = 2, no=1**

| | CASP questions (question 10 is not scorable) | | | | | | | | | |
|---|---|---|---|---|---|---|---|---|---|---|
| **Article authors** | **1** | **2** | **3** | **4** | **5** | **6** | **7** | **8** | **9** | **Total score** |
| Ahlstrom *et al.* 2017 | 3 | 3 | 3 | 2 | 3 | 1 | 3 | 3 | 2 | 24 |
| Andersen *et al.* 2014 | 3 | 3 | 3 | 3 | 3 | 1 | 3 | 3 | 3 | 27 |
| Angel *et al.* 2012 | 3 | 3 | 3 | 2 | 3 | 1 | 3 | 2 | 3 | 23 |
| Ashby *et al.* 2010 | 3 | 3 | 3 | 2 | 3 | 1 | 3 | 2 | 3 | 23 |
| Brooks *et al.* 2013 | 3 | 3 | 3 | 3 | 3 | 1 | 3 | 3 | 3 | 25 |
| Buus *et al.* 2015 | 3 | 3 | 3 | 3 | 3 | 3 | 3 | 3 | 2 | 26 |
| Coole *et al.* 2010 | 3 | 3 | 2 | 3 | 3 | 2 | 3 | 2 | 2 | 23 |
| Coutu *et al.* 2010 | 3 | 3 | 3 | 3 | 3 | 1 | 3 | 3 | 3 | 25 |
| Coutu *et al.* 2011 | 3 | 3 | 3 | 2 | 3 | 1 | 3 | 3 | 3 | 24 |
| Coutu *et al.* 2013 | 3 | 3 | 3 | 3 | 3 | 1 | 3 | 3 | 3 | 26 |
| Crooks 2007 | 3 | 3 | 3 | 2 | 3 | 1 | 3 | 3 | 3 | 24 |
| Dionne *et al.* 2013 | 3 | 3 | 3 | 2 | 3 | 1 | 3 | 3 | 3 | 24 |
| Edén *et al.* 2007 | 3 | 3 | 3 | 3 | 3 | 1 | 3 | 3 | 2 | 24 |
| Fassier *et al.* 2015 | 3 | 3 | 3 | 2 | 3 | 2 | 3 | 2 | 3 | 24 |
| Gard & Sandberg 1998 | 3 | 3 | 3 | 2 | 3 | 1 | 1 | 2 | 3 | 21 |
| Glavare *et al.* 2012 | 3 | 3 | 3 | 2 | 3 | 3 | 3 | 3 | 3 | 26 |
| Grataloup *et al.* 2016 | 3 | 3 | 3 | 3 | 3 | 1 | 3 | 3 | 3 | 25 |
| Hansson *et al.* 2001 | 3 | 3 | 3 | 2 | 3 | 1 | 3 | 3 | 3 | 24 |
| Hansson *et al.* 2006 | 3 | 3 | 3 | 3 | 3 | 1 | 3 | 3 | 3 | 25 |
| Johansson *et al.* 1997 | 3 | 3 | 3 | 3 | 3 | 3 | 1 | 3 | 3 | 25 |
| Juuso *et al.* 2016 | 3 | 3 | 3 | 3 | 3 | 2 | 3 | 3 | 3 | 26 |
| Kalsi *et al.* 2016 | 3 | 3 | 3 | 2 | 3 | 1 | 3 | 3 | 3 | 24 |
| Kvam *et al.* 2013 | 3 | 3 | 3 | 3 | 3 | 3 | 3 | 3 | 2 | 26 |

For peer review only

| | | | | | | | | | |
|---|---|---|---|---|---|---|---|---|---|
| Kvam & Vik 2015 | 3 | 3 | 3 | 3 | 3 | 1 | 3 | 3 | 3 | 25 |
| Liedberg & Henriksson 2002 | 3 | 3 | 3 | 2 | 3 | 2 | 3 | 3 | 3 | 25 |
| Magnussen *et al.* 2007 | 3 | 3 | 3 | 3 | 3 | 2 | 2 | 2 | 3 | 24 |
| McCluskey *et al.* 2011 | 3 | 3 | 3 | 3 | 3 | 1 | 3 | 3 | 3 | 25 |
| McCluskey *et al.* 2014 | 3 | 3 | 3 | 3 | 3 | 2 | 3 | 3 | 3 | 26 |
| Nilsen & Anderssen 2014 | 3 | 3 | 3 | 3 | 3 | 1 | 3 | 3 | 3 | 25 |
| Nordqvist *et al.* 2003 | 3 | 3 | 3 | 3 | 3 | 1 | 1 | 3 | 3 | 23 |
| Patel *et al.* 2007 | 3 | 3 | 3 | 3 | 3 | 1 | 2 | 3 | 3 | 24 |
| Rydstad *et al.* 2010 | 3 | 3 | 3 | 3 | 3 | 3 | 3 | 3 | 3 | 27 |
| Saunders *et al.* 2015 | 3 | 3 | 3 | 3 | 3 | 2 | 3 | 3 | 3 | 26 |
| Scheermesser *et al.* 2012 | 3 | 3 | 3 | 3 | 3 | 3 | 3 | 3 | 3 | 27 |
| Shaw & Huang 2005 | 3 | 3 | 3 | 2 | 3 | 2 | 3 | 2 | 2 | 23 |
| Sjöström *et al.* 2011 | 3 | 3 | 3 | 3 | 3 | 2 | 3 | 2 | 3 | 25 |
| Soeker *et al.* 2008. | 3 | 3 | 3 | 3 | 3 | 2 | 3 | 3 | 3 | 26 |
| Soklaridis *et al.* 2010 | 3 | 3 | 3 | 2 | 3 | 2 | 3 | 3 | 2 | 24 |
| Svensson *et al.* 2010 | 3 | 3 | 3 | 3 | 3 | 1 | 3 | 2 | 3 | 24 |
| Williams-Whitt *et al.* 2016 | 3 | 3 | 3 | 2 | 3 | 1 | 3 | 3 | 3 | 24 |
| Wynne-Jones *et al.* 2011 | 3 | 3 | 3 | 3 | 3 | 1 | 3 | 3 | 3 | 25 |

For peer review only

## Supplementary file 2

The remaining 13 conceptual categories that underpin the three key categories described in the main paper and summarised in Table 2 are now explained.

### Societal expectations

Many people living with chronic pain felt that society, institutions, family, friends and the media expected them to work and that those who did not do so were portrayed negatively and perceived as a burden.[47] This contributed to them feeling they were outsiders who were judged and discriminated against and for some this motivated them to return to work so they could contribute and belong.[36 59]

### Meaning of work

The meaning of work for each individual plays a part in whether they return or not. For some work provides financial security and independence[45] but for others there is a strong moral work ethic influenced by their upbringing or it is a way of strengthening self-esteem[36] and achieving fulfilment in life.[48] A lack of meaning in work when combined with a chronic pain condition can lead to demotivation in relation to work return[59] whereas for some work was central to their identity and purpose and loss of this aspect of their lives was perceived as devastating.[39 42 44 52] Social contact and relationships with others and feeling needed and valued are an important aspect of work for some people.[48 61]

A number of conflicts were highlighted in this conceptual category. One was the differing perceptions of employees with low back pain and those of their employers. Some employers felt that employees perceived sick leave as a right and this formed part of a culture of entitlement they perceived was encouraged by the unions which made employees demotivated about returning to work.[60] However this was a view strongly contested by the employees in the same study who reported they would often accept modified jobs in order to return to work, even if not appropriate to them, due to the tough economic climate and fear of losing their livelihood.[60]

For peer review only

Another conflict was that of balancing the demands of work with those of family life. Competing priorities sometimes meant that some chose to focus on family rather than paid work as they were unable to balance the two.[46]

**Autonomy**

Concepts in this category focus on the individual's ability to have control or agency in relation to their pain and their work situation. There was a sense that if they had control over their pain then this was the key to having autonomy in other areas including return to work.[45] Being allowed some control and flexibility at work, for example in the hours they worked, was seen as a pre-requisite by some for returning.[36] Psychological distress, including anxiety and depression, was linked with a perceived lack of control over pain and as a result return to work became a secondary issue[12] whereas opportunity for job control was a motivating factor in relation to returning to work.[38]

**Self-belief/ self-efficacy**

Some studies indicated that people with chronic pain had low self-esteem and a pessimistic outlook about their ability to handle work[49] and this related to concerns about their ability to meet the job demands, obtain help from others and manage their pain.[57]

**Health and illness and pain representations impact on return to work**

The way people think about their pain and the mental representations they form in relation to beliefs about it's cause and their perception of its impact on their lives [29] are seen as very important in relation to their pain experience and return to work. A clear distinction was made between those who perceived themselves as disabled by the pain and therefore unable to work [28 31 32 44 50] and those who accepted the pain as part of their lives but something they could exert some control over and therefore felt able to work.[31 39 54] There was a perception amongst some employers that attitudes were influenced by family and the community in which people grew up.[60] However, among people with pain, there was a resistance to and even rejection of the idea of psychosocial influences on pain.[31 56] Some studies asserted

For peer review only

that the way people with pain, often incorrectly visualised their injuries and formulated explanatory models led to a fear of movement and this had negative implications for their work life.[27]

**Influence of pre-return to work support and rehabilitation**

A number of studies in the review were evaluating the impact of return to work interventions and rehabilitation programmes.[26 29 33 39 54] Participants were largely positive about the intervention received and the strategies for managing pain and life they had developed but those who did not return to work experienced anxiety, disappointment, loneliness [39] and some felt useless[56] or a sense of powerlessness and guilt.[26]

**Not being understood**

The concept of not being understood mainly arose when participants were describing difficulties in relationships with health professionals, for example physicians not understanding their work situation[59] or not listening or taking them seriously in relation to their pain.[52] However, the same phenomenon occurred even with people's closest relatives, leaving them with a sense of abandonment.[54]

**Being believed**

People with chronic pain often struggled with not being believed or trusted and this was evident when employers talked about people taking sick leave that was not perceived as legitimate.[63] This feeling of being judged and doubted and having to justify absence or limitations became an obstacle to returning to work.[37 59] The pursuit of authenticity also became apparent from the perspective of people claiming benefits.[50] These individuals felt the need to stress their desire to work but also to emphasise how the severity of their pain condition was preventing them from doing so.[50]

**Impact of and on family**

For peer review only

People with chronic pain often rely on family members for practical support[56 59] which leads to a renegotiation of roles and responsibilities[54] and financial difficulties are prevalent.[27]

Significant others are also seen to be highly influential in terms of their beliefs and thinking in relation to pain and return to work. Some studies have highlighted sceptical views of significant others in relation to treatment received and pessimism about return to work and support that would be offered[50 51] and sometimes their well-intentioned support reinforces a position of disability and legitimacy and this reduces the possibility of gaining employment.[28]

The conflicting demands of family and work were also reported as a challenge for people with chronic pain with women sometimes choosing to prioritise family.[48 47]

**Mismatch between employee and employer expectations of return to work**

Participants expressed a fear of letting employers down and not being able to fulfil work expectations.[12 58] Some were also fearful of re-injury.[57] Finding modified work was difficult and some felt the employer would rather dismiss them than find them a suitable job.[58] High demands for effectiveness and productivity made it difficult to return to work[49] as people with pain were concerned they would not be able to achieve the required quality, quantity or speed of work.[57] Information given to employers about health problems or limitations was perceived as insufficient or incorrect which led to misunderstandings and distorted employer expectations.[53] Difficulties arose in relationships with colleagues especially where expectations of employees with health problems were lower than for other employees doing the same job.[53] Participants felt they would want to do as much as their colleagues and not be a burden on them.[30 38 61]

**Social isolation as a consequence of pain**

One of the consequences of chronic pain was a withdrawal from social networks and this was partly linked with financial and physical restrictions.[27 48] This led to loneliness and a sense of being abandoned by those around and therefore lacking the support needed to enable return to work.[52 54]

For peer review only

**System factors (healthcare, social security and workplace systems)**

System factors influencing return to work were within healthcare, social security and workplace systems. Delays in accessing appropriate healthcare, for example waiting lists for specialists, diagnostic testing and rehabilitation programmes, interfered with the return to work process.[12 55 60] Another issue was that employers did not always feel they could accommodate injured workers hospital appointments in work time and so preferred that they remained off sick until they were able to fulfil their work hours.[60]

Social security authorities were sometimes seen as unhelpful and inflexible benefits arrangements caused economic uncertainty for people wanting to make a gradual transition into work.[49] Interactions with social insurance personnel were perceived as difficult with conflict arising when staff put pressure on people with pain to complete training or enter employment that was deemed unsuitable or not in line with their interests.[49 55]

Finally workplace systems delayed work return through inadequate policies[37] and a perceived lack of education on disability management procedures.[59] There also appeared to be a lack of trust in occupational health who were seen to be on the side of the employer and more concerned with absence management than supporting people to return to work.[30]

**Finance and benefits**

Many people with chronic pain had serious concerns about their finances.[41 43 56] For some this was linked with social security and disability benefits and the economic insecurity of moving back into work.[12 49] Some receiving state support struggled with shame whereas others felt they were entitled to it[43] or saw no alternative.[41] Some argued there should be greater flexibility for people with fluctuating musculoskeletal diseases to allow them to make a gradual return to work without incurring financial hardship.[34]

For peer review only

**Supplementary File 3 - Concepts within each conceptual category**

| Conceptual category | Concepts |
|---|---|
| Managing pain | 1. Adaptions via new knowledge of how to manage daily occupations<br>2. Being out in nature – pain management facilitator<br>3. Controlling pain<br>4. Coping with pain<br>5. Coping with symptoms<br>6. Developing individual strategies to deal with pain<br>7. Fatigue<br>8. Fluctuating work status<br>9. Keeping pain at bay<br>10. Knowledge of limits and listening to body<br>11. Learning to manage whiplash associated disorder - a rehabilitation process<br>12. Management of back pain<br>13. Previous management of back pain<br>14. Mastering life despite pain<br>15. New knowledge about how to manage daily life<br>16. Pain management linked with RTW<br>17. Pain management strategies<br>18. Pain representations underpin strategies to manage condition<br>19. Passive coping strategies<br>20. Patients' coping strategies<br>21. Physical activity<br>22. Strategies for managing long-term pain<br>23. Pain representations underpin strategies to manage condition<br>24. Passive coping strategies<br>25. Patients' coping strategies<br>26. Physical activity<br>27. Strategies for managing long-term pain<br>28. Strategies to prevent pain<br>29. Treatment for pain<br>30. Use of sick leave<br>31. When no treatment helps self-care management strategies develop<br>32. Working to control the pain<br>33. Being under control of pain and fear of pain<br>34. Commuting (as an obstacle to work)<br>35. Difficulties caused by back pain<br>36. Fear of reinjury<br>37. Impact of back pain on ability to work (for those at work and those not at work)<br>38. Impact of health on work<br>39. Impact of pain medication on ability to work<br>40. Impact of pain on doing work in a satisfactory way<br>41. Living with uncertainty<br>42. Negative impact of chronic pain on ability to work<br>43. Negative impact of pain on wellbeing and daily activities<br>44. Pain and somatic symptoms<br>45. Painful condition is a barrier to working<br>46. Persistent pain is an important obstacle to return to work |

For peer review only

| | |
|---|---|
| | 47. Return to employment and fear of movement<br>48. The body as an obstacle to working (pain and fatigue)<br>49. Unpredictable pain difficult with respect to work |
| Managing relationships | 1. Active engagement of supervisor<br>2. Asking for help is problematic for people with chronic pain<br>3. Attitude of employer in the workplace<br>4. Attitudes towards presenteeism (managers and employees perspectives)<br>5. Being needed at work<br>6. Colleague support important<br>7. Communication and contact (between managers and employees)<br>8. Co-workers and employers attitudes, disbelief and lack of understanding<br>9. Earlier negative workplace experiences<br>10. Employers limited understanding and support<br>11. Fear of disclosing pain to employer<br>12. Gap in work history and disclosure<br>13. Harassment from colleagues due to modified work<br>14. Impact of employees with job restrictions on supervisors and managers<br>15. Impact of sickness absence on others<br>16. Individuals (workers, colleagues, managers) barriers<br>17. Individuals facilitators (collaboration with colleagues support empathy problem solving mutual trust)<br>18. Interpersonal conflict with colleagues; being judged, justify the pain<br>19. Lack of collaboration and understanding from employer is an obstacle to return to work<br>20. Lack of communication between manager and the team about RTW<br>21. Lack of support and communication with line manager<br>22. Lack of support from line manager for injured workers<br>23. Lack of understanding from employer<br>24. Line manager role important<br>25. Maintaining contact with absent employee<br>26. Managerial attitude and effort<br>27. Managerial autonomy<br>28. Mutual distrust between employees and their managers and colleagues<br>29. Negative response from employer<br>30. Peer conflict<br>31. Physical barriers not significant obstacle to maintaining employment<br>32. Psychosocial environment (at work)<br>33. Psychosocial factors at work influence RTW management<br>34. Reassignment of workers to other areas due to physical demands of job causes tension with supervisors due to perceived injustice<br>35. Relationship between managers and employees<br>36. Relationship with employer<br>37. Relationships with supervisors and colleagues important to work satisfaction<br>38. Reluctance of employer to take on injured worker with gaps in employment history |

For peer review only

| | |
|---|---|
| | 39. Responsibility for workmates<br>40. Social tensions in the workplace<br>41. Stigmatisation and blame<br>42. Struggling interactions with stakeholders<br>43. Support from employer and workmates<br>44. Supportive work environment<br>45. Supportive work environment and manager key to RTW success<br>46. Sympathy from manager if fellow back pain sufferer<br>47. Treatment from line manager inequitable<br>48. Understanding from an employer<br>49. Work relationships influence RTW<br>50. Working relations<br>51. Workmates attitudes |
| Making workplace adjustments | 1. Being marked out as different in the workplace<br>Austerity and economic climate<br>2. Competitive economic climate with restructuring and workforce reduction is a barrier for RTW<br>3. Economic climate impacts on ability to take sick leave and make work adjustments<br>4. Fast management turnover, lack of latitude in decision making and fear of increasing costs and claims for better working conditions are barriers to RTW<br>5. Job availability and competitive job markets<br>6. Work restructuring (labour market)<br>Flexible working<br>7. Flexibility from employers re hours facilitates RTW<br>8. Flexible working a pre-requisite for RTW<br>9. Impossibility of a gradual return to work is an obstacle to return<br>10. Modified hours of employment<br>11. Policy and programme recommendations<br>12. Flexible work hours<br>13. Job sharing and work-from-home<br>14. Work place adjustments<br>Involve managers and colleagues<br>15. Communication quality<br>16. Maintaining routines for sharing information about work accommodations with colleagues<br>Manager knowing options<br>17. Absenteeism destabilises work organisation and makes work accommodations challenging<br>18. Accommodation demands<br>19. Job aptitude restriction certificates<br>20. Lack of pre-planning for RTW makes job accommodations and communication with colleagues challenging<br>21. Poor matching of the worker and the work<br>22. Process of accommodation of back injured workers<br>Resources for decision making about accommodations<br>23. Information - Accommodation options<br>24. Information - Employee abilities<br>25. Information - Job demands |

For peer review only

| | |
|---|---|
| | 26. Information - Medical restrictions<br>27. Organisational support<br>28. Supervisors return to work experience<br>29. Size of workplace and difficulties of modified duties<br>30. Support from line managers over-cautious<br>31. Work modifications - assistance from Occupational Health<br>32. Work organisation and the challenges of work accommodations<br>Not consulted or involved in decision making.<br>33. Modified duties<br>34. Perceived lack of choice and control in relation to modified duties<br>35. Possibility of work adaptations and confidence and ability to negotiate adaptations with employer<br>36. Stakeholder perspective<br>37. Psychosocial stressors<br>38. Work modifications - patient control<br>Personal factors<br>39. Age and educational status - perceived as obstacle to finding work by people with back injury<br>40. Personal obstacles - qualifications and experience<br>41. Personal obstacles to RTW - older age<br>42. Resistance to change<br>Reducing demands of job or physical adjustments<br>43. Adjustment of work demands upon return to work<br>44. Challenges to work participation (to different type of work and work adjustments)<br>45. Change to less physically demanding job<br>46. Impossibility of being assigned lighter duties and working at one's own pace is an obstacle to return to work<br>47. Improvement of work environment and working conditions<br>48. Physical accommodations<br>49. Provision of appropriate modified work can be challenging and complex<br>50. Work modifications influence RTW possibilities<br>51. Working conditions - physical work<br>Type of job influences RTW<br>52. Benefits of self-employment<br>53. Physical stressors (working at lower surface areas and different weights) cause constant pain<br>54. Sharing staff over different departments makes work accommodations and assessment of work demands difficult<br>55. Type of job- profession influences RTW |
| Autonomy | 1. Autonomy<br>2. In the hands of the professionals - reduced control over life situation<br>3. Increased job control and contact with supervisors<br>4. Influencing factors for RTW - internal and external<br>5. Lack of agency<br>6. Locus of control influences return to work<br>7. Making sense of intervention - regaining control of situation<br>8. Own agency is important |

For peer review only

| | |
|---|---|
| | 9. Own power and resources<br>10. Perceived lack of control influences ability to work<br>11. Psychological barriers to return to work (lack of control, anxiety, depression, loss of confidence, frustration)<br>12. Taking control of and responsibility for work and life situation |
| Self-belief/ self-efficacy | 1. Being needed at work<br>2. Changed self-image<br>3. Controlling RTW interactions with stakeholders (employer, health care, social insurance system, union, public employment service)<br>4. Low self- evaluation of work ability and low self esteem<br>5. Obtaining help at work<br>6. Patient identity (when working and not working)<br>7. Positive coping linked with RTW self-efficacy<br>8. Positive self-identity a beneficial consequence of employment<br>9. Psychological effects of chronic pain affect RTW confidence<br>10. Relationship with family influences self-confidence and esteem<br>11. Satisfaction with self-image; confidence<br>12. Self-confidence through working<br>13. Self-efficacy<br>14. Self esteem<br>15. Self-image in relation to work<br>16. Self-identity<br>17. Unintended consequences - physical bodily changes post-injury like weight gain affect emotional readiness to return to work<br>18. Work morale |
| Being believed | 1. Being judged by colleagues<br>2. Disbelief from physicians<br>3. Distrustful attitude of the medical profession<br>4. Feeling doubted<br>5. Having to justify pain condition in the workplace<br>6. Legitimacy of absence and perceptions of others<br>7. Legitimising back pain<br>8. Legitimacy<br>9. Not being believed<br>10. Personally and socially legitimate explanations of LBP important<br>11. Pursuit of authenticity<br>12. Rights and responsibilities<br>13. Stigmatisation |
| Impact on and of family | 1. Being a 'good' significant other<br>2. Cultural differences in family support between women and men<br>3. Family support<br>4. Impact of family<br>5. Loss of social roles<br>6. Participation in work - a family matter<br>7. Relationship changes<br>8. Re-negotiation or loss of work role<br>9. Return to work is dependent on a cure (significant other viewpoint) |

For peer review only

| | |
|---|---|
| | 10. Role of significant others is important in return to work<br>11. Unpaid work (home, family, carer responsibilities)<br>12. Waiting for an answer (diagnosis, treatment, cure) (significant other viewpoint)<br>13. We have come to the end of the road (treatment options exhausted) (significant other viewpoint) |
| Not being understood | 1. Communication in rehab - cultural differences cause problems<br>2. Language barriers<br>3. Talking at cross purposes with health professionals<br>4. Congruence between clinicians understanding of workers representations and actual worker's representations during work rehab<br>5. Cultural factors influence RTW - family attitudes, language barriers, cultural beliefs<br>6. Differences between clinical judgement and workers representations during work rehab<br>7. Difficult to explain the pain<br>8. Lack of client centredness<br>9. Medical discourse of work participation - focus on pain rather than RTW |
| Finance and Benefits | 1. Finances<br>2. Financial concerns<br>3. Financial - job security<br>4. Interaction with benefits organisation<br>5. Permitted work<br>6. Limited staff skills in benefits organisation<br>7. Looking for a different way of living - transition to disability pension<br>8. Need for financial security<br>9. Part-time work by people receiving disability income assistance<br>10. State as supporter |
| Health and illness and pain representations | 1. Acceptance challenges - Difficulties in acceptance of pain and limitations impacts on participation in work<br>2. Acceptance of chronic pain as a long term disability is a barrier to return to work<br>3. Acceptance of limitations<br>4. Acceptance of pain as part of life facilitates RTW<br>5. Accepting the inability to work<br>6. Barriers to rehabilitation<br>7. Cultural influence on psychological factors<br>8. Beliefs about causality of back pain<br>9. Beliefs about course of illness and the sick role<br>10. Beliefs about treatment and effective management of LBP<br>11. Cause and meaning of back pain<br>12. Crystallising the abnormal pain representation<br>13. Cultural factors influence RTW - family attitudes, language barriers, cultural beliefs<br>14. Cultural factors influencing return to work (language and passive coping strategies) |

For peer review only

| | |
|---|---|
| | 15. Explanatory models of illness<br>16. Illness beliefs coherent<br>17. Impact of pain representations on return to work<br>18. Integrating explanations into daily life<br>19. Loss of ability<br>20. Loss of hope<br>21. Need to construct their own models of pain<br>22. Resignation to permanent effect of back problem on employment status in those not working and their significant others |
| Meaning of work | 1. Competing priorities mean work not necessarily prioritised<br>2. Effort to remain in or return to pre-injury jobs<br>3. Effort to return to employment following job loss<br>4. Employee motivation<br>5. Family orientated considerations take priority over working<br>6. Fulfilment in a work role<br>7. Goal orientated participation (work related achievements and values)<br>8. Importance of work<br>9. Lack of meaning and satisfaction in work<br>10. Meaning of work<br>11. Meaningful job- highly needed by others<br>12. Mentality (outlook) in relation to determination to RTW<br>13. Moral aspects of absence and presenteeism<br>14. Moral stance - importance of work<br>15. Motivation and entitlement<br>16. Motive for RTW<br>17. Organised time structure difficult to maintain without work<br>18. Participation constantly changing (feminine perspective)<br>19. Positive perceptions of work<br>20. Prioritising of work and home is an issue<br>21. Regaining identity (as a worker)<br>22. Sense of coherence<br>23. Sense of coherence and involvement in work, friends and family<br>24. Social aspects of work<br>25. Work as a source of security and independence<br>26. Work on hold |
| Mismatch of expectations | 1. Ability to do as much work as others<br>2. Fear of letting employers down<br>3. Fear of re-injury<br>4. Insufficient or incorrect information about health problems of the returning employee<br>5. Meeting job demands<br>6. Not able to fulfil work requirements<br>7. Own expectations in relation to RTW (optimistic or pessimistic)<br>8. Participating at before (masculine perspective)<br>9. Support expectations<br>10. Workplace productivity demands<br>11. Workplace support |

For peer review only

| | |
|---|---|
| Social isolation as a consequence of chronic pain | 1. Abandoned by those around (family, friends and colleagues)<br>2. Feeling on their own<br>3. Impact on social relations - many women with fibromyalgia fail to maintain social network due to demands of work and family<br>4. Loneliness in pain<br>5. Paid work - the struggle for social capital<br>6. Social isolation |
| Influence of return to work support and rehabilitation | 1. "A light at the end of the tunnel"; support, hope, new knowledge<br>2. A light in the tunnel - experience of work rehab programme<br>3. Hope of returning to work through rehab<br>4. Support<br>5. Believing in the intervention - effectiveness of exercise to manage LBP<br>6. Close social network – family, rehab team<br>7. Difficulty accessing worker representations and problem targeting in work rehab<br>8. Feelings about outcome of rehab<br>9. Function-centred treatment<br>10. Patients' expectations of treatment<br>11. Goals of rehabilitation - patient's perspective<br>12. Goals of rehabilitation - return to work<br>13. Joining a physical exercise programme<br>14. Lack of access to information or support groups is an obstacle to return to work<br>15. Mismatch of goals - patient and programme<br>16. Rehabilitation by activity and exercise<br>17. Specialised vocational rehab support needed<br>18. Support is important<br>19. Unsuccessful responses to intervention (rehabilitation) |
| System factors | *1. Health care system*<br>2. Healthcare barriers<br>3. Lack of communication, lack of coordination and fear of communication within compensation and health care systems is a barrier to RTW<br>4. Lack of knowledge in primary care and no support from social insurance office<br>5. Slowness of health care system is an obstacle to return to work<br>*6. Social security, insurance, unemployment office system*<br>7. From Social Insurance Office<br>8. From unemployment office<br>9. Inefficiency of the insurance companies<br>10. Lack of support from social security authorities<br>*11. Workplace system*<br>12. Inadequate workplace policy<br>13. Lack of education on disability management procedures by employers and rehabilitation professionals<br>14. Occupational health is for employers not employees<br>15. OH employer orientated - unequal relationship<br>16. Organisational policies - Return to work policies |

For peer review only

| | |
|---|---|
| | 17. Poor communication between stakeholders (doctors and employers about back condition and lighter duties duration)<br>18. Systems factors - workplace and union policies compensation system and healthcare system are barriers to RTW process<br>19. Wage support programmes awarded to employee<br>20. Workplace system barriers<br>21. External context barriers<br>22. Workplace barriers<br>23. Workplace system facilitators<br>24. External context facilitators<br>25. Workplace facilitators |
| Societal expectations | 1. Experiences of societal expectations of participation in work<br>2. Feeling of being outsider in society; work part of natural life<br>3. Unsupportive society |

For peer review only

**Enhancing transparency in reporting the synthesis of qualitative research: the ENTREQ statement**

| No | Item | Guide and description | Reported |
|---|---|---|---|
| **1** | Aim | State the research question the synthesis addresses. | p2 and p4 |
| **2** | Synthesis methodology | Identify the synthesis methodology or theoretical framework which underpins the synthesis, and describe the rationale for choice of methodology *(e.g. meta-ethnography, thematic synthesis, critical interpretive synthesis, grounded theory synthesis, realist synthesis, meta-aggregation, meta-study, framework synthesis).* | p5 |
| **3** | Approach to searching | Indicate whether the search was pre-planned (*comprehensive search strategies to seek all available studies)* or iterative (*to seek all available concepts until they theoretical saturation is achieved)*. | p5 |
| **4** | Inclusion criteria | Specify the inclusion/exclusion criteria *(e.g. in terms of population, language, year limits, type of publication, study type).* | p5 |
| **5** | Data sources | Describe the information sources used (e.g. *electronic databases (MEDLINE, EMBASE, CINAHL, psycINFO, Econlit), grey literature databases (digital thesis, policy reports), relevant organisational websites, experts, information specialists, generic web searches (Google Scholar) hand searching, reference lists)* and when the searches conducted; provide the rationale for using the data sources. | p5 |
| **6** | Electronic Search strategy | Describe the literature search *(e.g. provide electronic search strategies with population terms, clinical or health topic terms, experiential or social phenomena related terms, filters for qualitative research, and search limits)*. | p5 |
| **7** | Study screening methods | Describe the process of study screening and sifting *(e.g. title, abstract and full text review, number of independent reviewers who screened studies).* | p7 |
| **8** | Study characteristics | Present the characteristics of the included studies *(e.g. year of publication, country, population, number of participants, data collection, methodology, analysis, research questions).* | p9-p17 |

For peer review only

| No | Item | Guide and description | Reported |
|---|---|---|---|
| **9** | Study selection results | Identify the number of studies screened and provide reasons for study exclusion *(e,g, for comprehensive searching, provide numbers of studies screened and reasons for exclusion indicated in a figure/flowchart; for iterative searching describe reasons for study exclusion and inclusion based on modifications t the research question and/or contribution to theory development).* | p7 |
| **10** | Rationale for appraisal | Describe the rationale and approach used to appraise the included studies or selected findings *(e.g. assessment of conduct (validity and robustness), assessment of reporting (transparency), assessment of content and utility of the findings).* | p6 |
| **11** | Appraisal items | State the tools, frameworks and criteria used to appraise the studies or selected findings *(e.g. Existing tools: CASP, QARI, COREQ, Mays and Pope*[25]*; reviewer developed tools; describe the domains assessed: research team, study design, data analysis and interpretations, reporting).* | p6 |
| **12** | Appraisal process | Indicate whether the appraisal was conducted independently by more than one reviewer and if consensus was required. | p6 |
| **13** | Appraisal results | Present results of the quality assessment and indicate which articles, if any, were weighted/excluded based on the assessment and give the rationale. | Supp file 1 |
| **14** | Data extraction | Indicate which sections of the primary studies were analysed and how were the data extracted from the primary studies? *(e.g. all text under the headings "results /conclusions" were extracted electronically and entered into a computer software).* | p6 |
| **15** | Software | State the computer software used, if any. | p6 |
| **16** | Number of reviewers | Identify who was involved in coding and analysis. | p6 |
| **17** | Coding | Describe the process for coding of data *(e.g. line by line coding to search for concepts).* | p6 |
| **18** | Study comparison | Describe how were comparisons made within and across studies *(e.g. subsequent studies were coded into pre-existing concepts, and new concepts were created when deemed necessary).* | p6-7 |

For peer review only

| No | Item | Guide and description | Reported |
|---|---|---|---|
| **19** | Derivation of themes | Explain whether the process of deriving the themes or constructs was inductive or deductive. | p7 |
| **20** | Quotations | Provide quotations from the primary studies to illustrate themes/constructs, and identify whether the quotations were participant quotations of the author's interpretation. | p21-30 |
| **21** | Synthesis output | Present rich, compelling and useful results that go beyond a summary of the primary studies (e.g. *new interpretation, models of evidence, conceptual models, analytical framework, development of a new theory or construct).* | p31-35 |

From Equator Network. Tong A, Flemming K McInnes E Oliver S & Craig J (2012) Enhancing transparency in reporting the synthesis of qualitative research: ENTREQ. BMC Medical Research methodology **12**:181

For peer review only

# The work of return to work. Challenges of returning to work when you have chronic pain: a meta-ethnography


| | |
|---|---|
| Journal: | *BMJ Open* |
| Manuscript ID | bmjopen-2018-025743.R1 |
| Article Type: | Research |
| Date Submitted by the Author: | 26-Feb-2019 |



| | |
|---|---|
| Complete List of Authors: | Grant, Mary; University of Warwick Warwick Medical School, Clinical Trials Unit<br>O-Beirne-Elliman, Joanne; University of Warwick Warwick Medical School, Clinical Trials Unit<br>Froud, Robert; University of Warwick Warwick Medical School, Clinical Trials Unit; Kristiana University College, Prinsens gate 7-9, 0152 Oslo, Norway, Department of Health Sciences<br>Underwood, Martin; University of Warwick Warwick Medical School, Clinical Trials Unit<br>Seers, Kate; University of Warwick Warwick Medical School, Warwick Research in Nursing |



| | |
|---|---|
| <b>Primary Subject Heading</b>: | Qualitative research |
| Secondary Subject Heading: | Qualitative research |
| Keywords: | PAIN MANAGEMENT, occupational health, return to work, metaethnography |


Note: The following files were submitted by the author for peer review, but cannot be converted to PDF. You must view these files (e.g. movies) online.

RISE metaethnog - Enhancing transparency in reporting the synthesis of qualitative research.docx

SCHOLARONE™ Manuscripts

# The work of return to work. Challenges of returning to work when you have chronic pain: a meta-ethnography

**BMJ OPEN**

**Corresponding author:**

Mary Grant, Warwick Clinical Trials Unit, University of Warwick, Coventry, UK

M.Grant.2@warwick.ac.uk

**Co-authors:**

Joanne O'Beirne-Elliman, Warwick Clinical Trials Unit, University of Warwick, Coventry, UK

Robert Froud, Warwick Clinical Trials Unit, University of Warwick, Coventry, UK and Institute of Health Sciences, Kristiania University College, Oslo, Norway.

Martin Underwood, Warwick Clinical Trials Unit, University of Warwick, Coventry, UK

Kate Seers, Warwick Research in Nursing, Warwick Medical School, University of Warwick, Coventry, UK

**Key words: chronic pain, return to work, meta-ethnography**

For peer review only

**ABSTRACT**

**Aim:** To understand obstacles to returning to work, as perceived by people with chronic non-malignant pain, and as perceived by employers and to develop a conceptual model.

**Design:** Synthesis of qualitative research using meta-ethnography.

**Data sources:** Eleven bibliographic databases from inception to April 2017 supplemented by citation tracking.

**Review methods:** We used the methods of meta-ethnography. We identified concepts, conceptual categories and developed a conceptual model and line of argument.

**Results:** We included 41 studies. We identified three core categories in the conceptual model; managing pain, managing work relationships and making workplace adjustments. All were influenced by societal expectations in relation to work, self (self-belief, self-efficacy, legitimacy, autonomy and the meaning of work for the individual), health/ illness/ pain representations, pre-return to work support and rehabilitation, and system factors (healthcare, workplace and social security). A mismatch of expectations between the individual with pain and the workplace contributed to a feeling of being judged, and difficulties asking for help. The ability to navigate obstacles and negotiate change underpinned mastering return to work despite the pain. Where this ability was not apparent, there could be a downward spiral resulting in not working.

**Conclusions:** For people with chronic pain, and for their employers, navigating obstacles to return to work entails balancing the needs of: (1) the person with chronic pain; (2) work colleagues; and (3) the employing organisation. Managing pain, managing work relationships and making workplace adjustments appear to be central, but not straightforward, and require substantial effort to culminate in successful return to work.

**[Word count = 252]**

For peer review only

***Strengths and Limitations of this study*** *(five bullet points - one sentence each)*

Strengths

- This is the first study to present employer and employee perspectives together.
- This study draws together what is known from qualitative studies to inform practice.
- This study highlights health and illness and pain representations in relation to return to work.

Limitation

- Only five studies covered employers' perspectives, so there are fewer data on employers' perspectives compared to the perspectives of people with chronic pain.

For peer review only

# INTRODUCTION

Chronic pain, defined as pain lasting three months or more[1], is a global public health problem affecting one-in-ten adults.[2] A 2017 mega-ethnography brought together eleven qualitative evidence syntheses to explore the experience of living with chronic non-malignant pain.[3] Previous reviews have identified the importance of the effect of chronic pain on people's work life.[4 5] Chronic pain is strongly associated with claiming disability and unemployment benefit in Australia[1] and with unemployment in the USA.[6] The obstacles to staying in work for people with musculoskeletal pain have previously been explored in a meta-ethnography[7] and factors promoting staying at work are the focus of a previous mixed methods systematic review.[8] A qualitative systematic review of the impact of chronic pain in the workplace[9] takes a broad perspective including impact on employment status, sickness absence and loss of productivity in contrast to a condition and gender-specific literature review focused on work and rehabilitation for women with fibromyalgia.[10] There is qualitative research on the perspective of doctors [11] but this is not considered further in this paper.

The lack of focus on return to work for people with chronic non-malignant pain and the perspective of employers presents a knowledge gap in existing reviews. Return to work can refer to the process of returning after a period of sick leave[12] or returning after a period of unemployment.[13] This review uses qualitative evidence synthesis to increase understanding of the obstacles to return to work for people with chronic pain and their employers, and this can then inform intervention development to support return to work.[4 14]

# METHODS

## Aims and objectives

This meta-ethnography explores experiences of returning to work, as perceived by people with chronic non-malignant pain and by employers and to develop a conceptual model.

## Study design

For peer review only

There are two main approaches to synthesising qualitative research, one that aggregates findings to describe the literature and one that aims to interpret findings and develop a conceptual understanding.[4 14 15] Meta-ethnography is an interpretative form of knowledge synthesis that was chosen for this study in order to both integrate and develop a greater understanding of existing knowledge and identify any other overarching concepts that would explain the data. The seven phases of meta-ethnography are outlined by Noblit & Hare (1988)[15] and elaborated on by Toye *et al.* (2014b).[14] These are 1) getting started by identifying area of interest 2) deciding what is relevant 3) reading and re-reading the studies 4) determining how the studies are related which involves creating a list of key phrases, ideas, metaphors and concepts 5) translating the studies into one another where direct comparisons are made and similar concepts are sorted into categories 6) synthesising the translations where researchers make sense of the conceptual categories to develop new knowledge and understanding and 7) expressing the synthesis. A line of argument was constructed by examining how the conceptual categories relate to each other.

**Identifying and appraising the review articles**

**Search methods**

**Study selection**

Eleven electronic bibliographic databases were searched (AMED; ASSIA; CINAHL; EMBASE; IBSS; MEDLINE; PsycINFO; Social Services Abstracts; Sociological abstracts; Web of Science; Westlaw) from inception up until 25th April 2017 supplemented by backwards and forwards citation tracking using SCOPUS. These databases were considered appropriate because in early scoping work we identified relevant studies in these databases. An academic support librarian undertook the initial search in collaboration with RF in December 2016 and this was updated by MG in April 2017 who continued the screening and selection of papers. The search terms used included “Chronic pain” and “Return to work (MeSH) OR Employment OR Employer OR Supported Employment (MeSH)”. In April 2017 two additional search terms were used, ‘pain’ to broaden search as

For peer review only

104 ‘chronic pain’ was not identifying all relevant papers and ‘qualitative’ as suggested by Shaw et al (2004)[16] to focus the search on studies with this type of methodology. Search strategy is detailed in Supplementary File 1. All qualitative studies using face-to-face interviews and focus groups which explored perceptions of obstacles to return to work, in employers and people who were off work, sick-listed and had chronic pain were included. Non-English language texts were excluded.

**Quality appraisal**

The quality of studies was evaluated using the Critical Appraisal Skills Programme (CASP) qualitative assessment tool.[17] A scoring system was utilised for CASP (yes = 3, can’t tell = 2, no = 1). A score of 20 or higher indicates the paper is deemed to be of satisfactory quality. The GRADE-CERQual (Grading of Recommendations Assessment Development and Evaluation - Confidence in the level of Evidence from Reviews of Qualitative research) approach was also completed.[18 19] Confidence in review findings was assessed based on four components: adequacy of data[20], coherence[21], methodological limitations[22] and relevance.[23]

**Analysis**

Initially, the first ten papers (in alphabetical order of author) were read by MG, KS and JOB in order to identify key ‘concepts’, the raw data of meta-ethnography.[14] These concepts are ideas drawn from the findings of the original papers. They are also known as second order concepts because they are the authors’ interpretations of the participant’s narratives (known as first-order concepts).[24] The participants’ narratives chosen by the author are examples of second-order concepts.[14] After reading these 10 papers, the concepts identified by each researcher were amalgamated through discussion and grouped into conceptual categories that the team then worked collaboratively to name. This took place over a series of three meetings. These conceptual categories are third-order concepts insofar as they are the researchers’ interpretations of second order concepts. All concepts were identified by all

For peer review only

three authors (KS MG JOB) even if exact wording differed, the concept was the same. This is the way that studies were translated and related to each other. The first author then proceeded to read the rest of the papers and continue this process of analysis. Five additional papers were also read by KS and JOB where MG felt a collaborative discussion would be helpful due to the nature and/ or findings of the studies. Thus 25% of papers were checked (n=10) then an additional 10% were checked (i.e. 35% in total) to ensure ratings and concepts were in agreement. All the included papers were uploaded to QSR International's NVivo 11 software[25] and nodes were created for the conceptual categories. The next stage was to make sense of these categories through further discussion, make decisions about which were the core categories and develop a line of argument and conceptual model[14] involving a further four meetings. Recurring and common concepts were compared across studies[15] where directly comparable (reciprocal translation) together they contributed to our line of argument. We did not find studies that stood in opposition (refutational translation). The line of argument makes a whole of something more than a sum of the parts.[15] MG, JOB and KS independently drew their own conceptual model before coming together to agree a model, which was revised through several discussions and the final version is presented in this paper. The culture described by Toye *et al.* (2013b)[26] of a core team that provided a safe environment in which to freely discuss, agree, disagree and change their position in relation to conceptual analysis was seen as a key strength, laying the foundations for a rigorous review. This approach was adopted in this review. We explored alternative interpretations and explanations including locus of control, navigating relationships, normalising participants' pain condition, agency but these were not supported as major concepts. Many of these concepts were subsumed in other categories.

**Patient and Public Involvement**

A patient and public representative was involved in the development of the research funding submission for the overall study as a co-applicant and endorsed the importance of the focus of this meta-ethnography recognising the central nature of obstacles to return to work.

For peer review only

## Results

### Search outcome and overview of studies reviewed

We include 41 papers and search outcome is illustrated by a flow chart in **Figure 1 [insert Figure 1 near here]**. The initial 3191 hits were screened by titles and abstracts, duplicates excluded and a further 1466 were excluded at this stage. Following the reading of full texts, papers were excluded as they were neither about chronic pain nor specifically about return to work. All studies that were critically appraised passed the first two screening questions of the CASP tool that related to whether there was a clear statement of the aims of the research and if qualitative methodology was considered appropriate to address the research goal.[17] CASP scores are presented in Supplementary File 2. Of the 41 articles included, 32 reported interview studies and nine focus group studies. Twenty-one studies were from Scandinavia (14 in Sweden, four in Norway, and three in Denmark), seven were from the UK, seven were from Canada, two in France and one each from Australia, South Africa, Switzerland and USA. Only five studies were from employer's perspectives. One study included in the review did not specify the type of chronic pain, but the majority of the studies involved people or employers of people with musculoskeletal pain, mainly affecting the back and neck and some were injury/ work related. Studies of people with musculoskeletal disease including arthritis, fibromyalgia and systemic lupus erythematosus were also included (Table 1).

For peer review only

178 **Table 1 Description of included studies**

179

| | Author and year of publication | Country | Type of pain | Number, gender and age of participants | Participants | Method of data collection | Methodological approach – Analysis |
|---|---|---|---|---|---|---|---|
| 1 | Ahlstrom *et al.* 2017[12] | Sweden | Neck pain | 16 women<br>Mean age of 54 | People with history of long-term sick leave in human service organisations | Interviews | Constructivist grounded theory approach |
| 2 | Andersen *et al.* 2014[27] | Denmark | Back or upper body | 4 men and 3 women aged 33-57 | Participants in chronic pain self-management programme or tailored physical activity programme | Semi-structured interviews | Systematic text condensation – thematic cross case analysis |
| 3 | Angel *et al.* 2012[28] | Denmark | Low back pain | 20 (65% women)<br>Mean age of 46 | Participants of counselling intervention addressing work place barriers and physical activity | Semi-structured clinical interviews | Narrative analysis |
| 4 | Ashby *et al.* 2010[29] | Australia | Chronic low back pain | 11 men aged 23-59 | Participants in a work hardening programme | Semi-structured interviews (ethnographic) | Thematic content analysis |
| 6 | Brooks *et al.* 2013[30] | England | Persistent non-specific low back pain of at least 12 weeks duration | 6 women and 3 men<br>Working participants (5) aged 45-52 (mean 49.2)<br>Unemployed participants (4) aged 51-63 (mean 57) and | Participants from hospital pain management clinic | Semi-structured interviews | Template analysis style of thematic analysis |

For peer review only

| | | | | | | | |
|---|---|---|---|---|---|---|---|
| | | | | their significant others | | | |
| 7 | Buus *et al.* 2015[31] | Denmark | Low back pain | 25 (56% women) (mean age 46.8) | People who had received counselling intervention designed to motivate them to change work routines and exercise | Semi-structured interviews | Interpretative thematic analysis |
| 8 | Coole *et al.* 2010[32] | UK | Low back pain | 13 women and 12 men aged 22-58 years (mean age 44.7) | People offered MDT back pain rehabilitation and concerned about ability to work due to low back pain | Semi-structured interviews | Thematic analysis |
| 9 | Coutu *et al.* 2010[33] | Canada | Persistent musculoskeletal pain - back pain (10), upper extremities (4), mixed (2) | 10 men and 6 women aged 25-56 (mean age 40) | Workers referred to work rehab programme | Semi-structured interviews | Narrative approach – content analysis |
| 10 | Coutu *et al.* 2011[34] | Canada | MSD –related pain for more than 12 weeks accepted and compensated by Quebec Workers Compensation Board<br><br>Back pain (10), upper | 16 workers - 10 men six women<br><br>Aged 25-56 years (mean age 40) | Referred to evidenced based work rehab programme by a third party payer | Semi-structured interviews | Content analysis |

| | | | | | | | |
|---|---|---|---|---|---|---|---|
| | | | extremities (4), mixed (2) | | | | |
| 11 | Coutu *et al.* 2013[35] | Canada | MSD –related pain for more than 12 weeks accepted and compensated by Quebec Workers Compensation Board<br>Back pain (8), upper extremities (2), both (2) | 12 workers (8 men and 4 women) aged 25-56 (mean age 31) and five clinicians | Participants from workers starting a work rehab programme at a hospital research centre | Multiple case study design-semi-structured interviews with workers and rehab clinicians at four points in time | Thematic analysis & constant comparison method (grounded theory) |
| 12 | Crooks 2007[36] | Canada | MSD (fibromyalgia, arthritis, RA, OA, Lupus) | 18 women aged 26-69 (mean age 44) | Women who developed MSD while involved in the labour market | In-depth interviews | Thematic analysis |
| 13 | Dionne *et al.* 2013[37] | Canada | Work-disabling back pain | Workers with work-disabling back pain<br>9 = returned to work (7 men, 2 women) aged 30-59<br>10= not returned or recently returned (7 men, 2 women) aged 30-60+ | Recruited through newspaper adverts | Focus groups (two) | Content analysis |
| 14 | Edén *et al.* 2007[38] | Swedish | MSD (type not specified) | 17 individuals (two men, 15 women) aged 41-62 years | People going back to work by means of the | Interviews | Inductive analysis relevant to research question |

For peer review only

| | | | | | | | |
|---|---|---|---|---|---|---|---|
| | | | | | Swedish 'resting disability pension' | | |
| 15* | Fassier *et al.* 2015[39] | France | Low back pain | Three employers, one manager and one worker | Recruited from work places with high rates of absence for low back pain – car maker, association providing home services for the dependent and two university hospitals | Interviews and focus groups | Qualitative content analysis |
| 15 | Gard & Sandberg 1998[40] | Sweden | Musculoskeletal pain (shoulder, neck and low back pain) for at least one year with a period of at least four weeks during that time | 10 patients (9 females and one male) aged 30-54 years (mean age 47) | People sick listed with musculoskeletal pain | Interviews with a low degree of structure | Phenomenological structural analysis |
| 16 | Glavare *et al.* 2012[41] | Sweden | Long-term musculoskeletal pain (whiplash, fibromyalgia, nerve injury (neck), arthrosis of foot) | 11 (8 women and 3 men) aged 22-58 (median age 39) | Participants in a multi-professional pain rehab programme followed by a coached work-training programme | Thematised research interviews | Grounded theory – constant comparative method |
| 17* | Grataloup *et al.* 2016[42] | France | Supervisors of people with musculoskeletal disorders | Employees' supervisors (61 charge nurses, and head nurses supervising one or more workers with restrictions for heavy | Staff from three public hospitals | 12 focus groups (charge nurses and head nurses separate) | Thematic qualitative analysis by constant comparison each focus group analysed before the next held |

For peer review only

| | | | | | | | |
|---|---|---|---|---|---|---|---|
| | | | | lifting or repetitive movements) | | | |
| 18 | Hansson *et al.* 2001[43] | Sweden | Spine-related pain | 5 people (4 female and 1 male) aged 51-64 (median 55) | People granted disability pension in 1996 | Interviews conducted as conversations – approach based on symbolic interactionism | Based on grounded theory |
| 19 | Hansson *et al.* 2006[44] | Sweden | Neck or low back pain (spine related pain) | 33 (20 women and 13 men) aged 32-61 years (median age 48) | Sick listed participants | Qualitative interviews | Qualitative analysis |
| 20 | Johansson *et al.* 1997[45] | Sweden | Undefined musculoskeletal pain disorders | 20 female patients aged 21 – 61 | Females sick listed due to MSD in urban health centre | Repeated thematic interviews | Grounded theory |
| 21 | Juuso *et al.* 2016[46] | Sweden | Fibromyalgia | 15 women aged 38-64 (median 54) | From a rehabilitation centre (4), associations for rheumatism and FM (11) | In-depth qualitative interviews | Hermeneutic approach |
| 22 | Kalsi *et al.* 2016[47] | UK | Chronic pain (type not specified) | 17 patients (8 male, 9 female) aged 18-65+ but majority (14/17) were 18-34 | Patients attending a 3 week high intensity pain management programme | Semi-structured focus group discussion | Thematic analysis |
| 23 | Kvam *et al.* 2013[48] | Norway | Prolonged musculoskeletal pain (unspecified pain in back, neck and shoulders due to fibromyalgia, | 4 men and 6 women aged 26-57 | Volunteers from people undergoing vocational rehabilitation | Semi-structured interviews | Constant comparative analysis |

For peer review only

| | | | | | | | |
|---|---|---|---|---|---|---|---|
| | | | arthritis, and rheumatism) | | | | |
| 24 | Kvam & Vik 2015[49] | Norway | Prolonged musculoskeletal pain (unspecified pain in back, neck and shoulders due to fibromyalgia, arthritis, and rheumatism) | 6 women, 4 men aged 26-57 | People undergoing vocational rehabilitation | In-depth interviews | Discourse analysis |
| 25 | Liedberg & Henriksson 2002[50] | Sweden | Fibromyalgia | 39 women aged 35-63 (mean 49.5) | Patients from a pain and rehab centre | Interviews | Analysed into categories and subcategories |
| 26 | Magnussen *et al.* 2007[51] | Norway | Back pain | 12 women, 5 men aged 38-56 years (mean age 49) | Part of a larger study evaluating the effect of a vocational rehabilitation related intervention | Three focus groups | Analysis of themes and subthemes |
| 27 | McCluskey *et al.* 2011[52] | UK | Persistent back pain | 5 dyads (4 male & 1 female claimants) – aged 29-54 years (mean age 40.2) | Disability claimants and their significant others | Semi-structured interviews | Template analysis |
| 28 | McCluskey *et al.* 2014[53] | UK | Persistent low back pain | 18 (9 benefits claimants - 5 males and 4 females aged 29-63 - mean age 48.1) and 9 significant others (6 females and 3 males aged 21-68 mean age – 49.7) | Work disability benefits claimants and significant others | Semi-structured interviews | Template analysis |

For peer review only

| | | | | | | | |
|---|---|---|---|---|---|---|---|
| 29 | Nilsen & Anderssen 2014[54] | Norway | Non-malignant chronic pain (neck and back pain, traffic injuries) | 10 men and 10 women aged 26-63 (in the year 2006) - mean age 42.7 | From a specialist pain clinic | Open ended interviews | Narrative analysis Phenomenological meaning condensation framework |
| 30 | Nordqvist *et al.* 2003[55] | Sweden | Back, neck or shoulder diagnoses | 13 women and 5 men | People who in 1985 were 25-34 years old and had a new sick leave spell of at least 28 days. | Five focus groups | Grounded theory coding and categorising |
| 31 | Patel *et al.* 2007[13] | UK | Chronic musculoskeletal pain | 38 patients (15 male 23 female) aged 29-62 years (mean age 49.4) | Unemployed and in receipt of long-term social welfare benefits | In-depth semi-structured interviews | Framework approach and thematic analysis |
| 32 | Rydstad *et al.* 2010[56] | Sweden | Whiplash Associated Disorders | 9 people (5 females, 4 males) aged 32-53 years | Participants of a work-oriented MDT rehab programme | Thematised interviews | Constant comparison method – grounded theory |
| 33 | Saunders *et al.* 2015[57] | Canada | MSK injury<br><br>Arm (1), knee (1), back injuries(7) | 9 people (5 females, 4 males) aged 34-56 years | People with long-term work disability and job loss due to an MSK injury from work rehab and chronic pain programmes | Interviews (27) with 9 people | Thematic analysis (phenomenological approach guided by life world concept) |
| 34 | Scheermesser *et al.* 2012[58] | Switzerland | Low back pain | 13 (9 men, 4 women) aged 38-60 years (mean age of men – 52, mean age of women 48) | Patients with a Southeast European cultural background attending a rehab centre in Switzerland | In-depth semi-structured interviews (5) & two focus groups | Qualitative content analysis |
| 35 | Shaw & Huang 2005[59] | USA | Occupational low back pain | Focus group - 28 people (15 male, 13 | Focus group participants - people recently (<6 months) | Focus group & interviews | Content analysis |

For peer review only - http://bmjopen.bmj.com/site/about/guidelines.xhtml

| | | | | | | | |
|---|---|---|---|---|---|---|---|
| | | | | female) aged 31-65 (mean age 46)<br><br>Interviewees – 23 people (11 male, 12 female) aged 25-64 (mean age 42.6) | returned to work after injury responding to newspaper advert<br><br>Interview participants - patients referred by physios from collaborating OH network | | |
| 36 | Sjöström *et al.* 2011[60] | Sweden | MSK disorders - mainly back and neck pain | 10 people (7 women, 3 men) aged 29-61 (mean age 48) | Attended a rehab programme and still on full time sick leave 2 years after completion | Semi-structured interviews | Qualitative content analysis |
| 37 | Soeker *et al.* 2008 [61] | South Africa | Back injury | 26 people (18 males, 8 females) aged 18 – 60 | Selected by random sampling from a hospital rehab department | Focus groups | Qualitative analysis |
| 38* | Soklaridis *et al.* 2010[62] | Canada | Low back pain – work-related injury | 59 stakeholders including 6 injured workers and 5 small and 9 large employers | Various contacts of the research team | Nine focus groups | Grounded theory approach |
| 39 | Svensson *et al.* 2010[63] | Sweden | Back neck or shoulder diagnosis | 13 women and 5 men | People aged 25-34 years old in 1985 and had a new sick leave spell of at least 28 days | Five focus groups | Descriptive and explorative method of analysis |
| 40* | Williams-Whitt *et al.* 2016[64] | Canada | Low back pain | 23 supervisors | Supervisors of back injured workers from 11 Canadian organisations | Semi-structured in-depth interviews | Constructivist grounded theory principles |

For peer review only - http://bmjopen.bmj.com/site/about/guidelines.xhtml

| | | | | | | | |
|---|---|---|---|---|---|---|---|
| 41* | Wynne-Jones *et al.* 2011[65] | Wales | Musculoskeletal pain | 18 employees with MSK pain (8 male, 10 female) mean age 49.7<br><br>20 managers (10 male, 10 female) mean age 44.8 | Two large public sector organisations | Semi-structured interviews | Thematic analysis |

180 *Employer studies

For peer review only

**Overarching conceptual categories**

A total of 342 concepts were clustered into 16 conceptual categories summarised in Table 2. The first column of Table 2 contains third order concepts. We worked with second order concepts and the second column of Table 2 is second order data some of which is illustrated with first order participant quotations. This table also highlights the CERQual profile. The three key conceptual categories identified by the team and are described in this section. The balancing of these three inter-related categories and the way they are influenced by other factors appears to be central to negotiating a successful return to work. The scope for managing pain and making adjustments in the workplace can be influenced by the quality of the relationship an individual has with their employer and/ or line manager and what is feasible within a particular work setting. The remaining 13 conceptual categories are described in more detail in Supplementary File 3. The concepts within each conceptual category are presented in Supplementary File 4.

**Managing pain**

Pain was seen as a major obstacle to return to work.[46 54 60] A plethora of strategies to manage it were described[43 47 54 56 59 60] including use of sick leave.[12 44]

> "They used the strategies doing a little at a time, taking continuous breaks, working slower and being aware of body posture and workloads. These strategies improved their endurance and prevented further pain."[56]

However, the strain of living with chronic pain meant fatigue also became a problem and low energy levels prevented work return.[60]

> "Pain developed and became continuous, was easily provoked by work tasks and relatively resistant to pain-controlling strategies. Life became strenuous and energy was reduced."[43]

For peer review only

The impact of pain on performance [65] and ability to attend and travel to work[50] along with the fear of pain exacerbation[33 47] were also problematic.

**Managing work relationships**

Interpersonal conflict and mutual mistrust can arise between people with pain and their employers and colleagues[39 63] and if relationships with supervisors are perceived as poor then this is demotivating in relation to work return.[40] Employers with few employees expressed reservations about how far to push an employee for fear of upsetting them and causing them to be off sick for longer than necessary.[62] Managers in a public sector study appeared to be walking a fine line between supporting employees, making sure colleagues were not adversely affected, and that services were delivered.[65] Asking for help was perceived as frustrating by people in pain, and incurred feelings of inadequacy and negativity.[27] Some struggled in their interaction with employers and tended to be passive, not believing their views were listened to, or valued, which led to difficulties in sustaining work return.[12] Unsympathetic employer attitude and a lack of understanding of the person's experience of pain were seen as a major obstacles to work return[13 36 47 56 61] but those employers with personal experience of pain were perceived as more sympathetic and empathic.[32 64]

> "One of the important employment related obstacles is the perception that employers have limited understanding about pain due to ignorance and a lack of awareness. However, patients do acknowledge that chronic pain is difficult to understand without personal experience."[13]

Team management responsibilities of regulating tension between colleagues was perceived as challenging when work restrictions for those with pain caused unequal work distribution leading to a sense of injustice.[42]

For peer review only

> "However, if duties were reduced indefinitely, with no extra cover, workers might feel that they were burdening their colleagues. There were doubts as to how long their colleagues support might continue" [32]

**Making workplace adjustments**

An economic climate of austerity was perceived as an obstacle to work due to reduced job availability and a competitive job market.[13] Reorganisations and rationalisation in the workplace meant jobs had changed and become more demanding and potentially difficult to adapt for people with a pain condition.[50] In this situation, age was also seen as influential with some feeling they were too old to retrain for a different kind of job.[13 61]

The type of job influenced work return decisions with physical work being perceived as more challenging with pain[42 61] and more highly skilled work providing greater scope for flexibility and adaptation.[30]

> "Modifying work hours and days is a potential accommodation for women who develop musculoskeletal diseases, but it is only appropriate in certain work environments where such flexibility is allowed." [36]

People with chronic pain often felt they were not consulted or involved in the decision making about workplace redeployment or adjustment and when desired modifications were not possible they could not return to work. [30 32 36 61] Managers' attitudes and efforts, [64] combined with effective routine methods of regular communication of changes made to colleagues[55] were seen as ways of improving the success of workplace adjustments. Managers did not always have the resources or know what options would be available for making these adjustments and saw the planning of these accommodations as an additional demand on their time.[64] Managers also felt that information about work restrictions from occupational health were not always realistic in the work setting and therefore difficult to implement.[42]

For peer review only

"Many charge and head nurses complained that occupational physicians formulated unrealistic restrictions that were impossible to respect due to work organization." [42]

A number of workplace adjustments were felt to be helpful including flexible hours or a reduction in hours but were not always forthcoming.[30 36 50] The possibility of a gradual return to work[37] working from home or participating in a job sharing programme[36] were also seen as helpful by people in chronic pain. Changes to the job itself including physical adjustments and a reduction in job demands were not always feasible, for example in a nursing[36], nursing assistant role[50] or a preschool teaching role.[49]

For peer review only

**Table 2 Conceptual categories, description of category, supporting studies and CERQual assessment**

| | Conceptual category - summary of review finding | Quotations from primary studies to illustrate conceptual category | Supporting studies | Adequacy (number of concepts – see list of concepts in Supplement -ary File 3) | Coherence (number of supporting studies) | Methodological limitations (see CASP scores in Supplementary File 1) | Relevance | Overall CERQual assessment of confidence in the evidence | Explanation of CERQual assessment |
|---|---|---|---|---|---|---|---|---|---|
| **1** | **Managing pain** - the impact of pain on return to work and how it can be managed | Chronic pain itself was the underlying barrier from which most other barriers to work stem. Overall, very few patients reported any attempts to plan for the future, primarily due to the unpredictable nature of the pain condition and physical mobility problems associated: "*I have no objections at all to go back to work. But, I thought about this. I don't know what I could do. I can't sit for very long. I can't stand for very long. Erm, in discomfort 99% of the time.*"[Male, 56] [13] | 12 13 29 30 33 37 40 41 43 44 46 47 50 52 54 56 58-60 65 | Richly described (49) | Fit between underlying data and review finding is very clear (20) | All CASP scores over 20 | Sweden 9<br>UK 5<br>Canada 2<br>Norway 1<br>Switzerland 1<br>USA 1<br>Australia 1<br><br>Three studies partially relevant as only included women not men. | High confidence | Graded as high in relation to adequacy, coherence, methodological limitations and relevance. Employee and employer studies support this finding. |
| **2** | **Managing work relationships** - impact of chronic pain on relationships with employers, line managers and colleagues | The existence of interpersonal conflict with colleagues or managers was mentioned as a barrier, as was mutual mistrust. For managers, overwork, role conflict between production | 12 13 27 30 32 36-42 45-47 50 51 55-57 61-65 | Richly described (51) | Fit between underlying data and review finding is very clear (25) | All CASP scores over 20 | Sweden 10<br>Denmark 1<br>UK 5<br>Canada 5<br>France 2<br>Norway 1<br>South Africa 1 | High confidence | Graded as high in relation to adequacy, coherence, methodological limitations and relevance. |

For peer review only

| | | | | | | | | | |
|---|---|---|---|---|---|---|---|---|---|
| | and how this is managed | targets and occupational health and a lack of hierarchic support were possible barriers. With colleagues, overwork and scepticism about medical problems could induce hostility and rejection. For workers with LBP, the feeling of being judged and having to justify absence, pain and limitations was perceived as a barrier. [39] | | | | | | | Employee and employer studies support this finding. |
| **3** | **Making workplace adjustments** - the scope and process for making changes to the job, work conditions or environment | Our data suggest that the ability of participants to remain in employment was in part influenced by the nature of their work (whether or not adaptations could be made to enable employees to continue in post despite their symptoms) and in part due to patients' confidence and ability to negotiate adaptations with their employers (significant others often described themselves as being an important source of support for the patient in this context). [30] | 12 13 28 30-32 36-39 42 44 49 50 54 55 60-62 64 | Richly described (55) | Fit between underlying data and review finding is very clear (20) | All CASP scores over 20 | Denmark 2<br>Sweden 6<br>UK 3<br>Canada 4<br>Norway 2<br>France 2<br>South Africa 1 | High confidence | Graded as high in relation to adequacy, coherence, methodological limitations and relevance. Employee and employer studies support this finding. |

For peer review only

| | | | | | | | | | |
|---|---|---|---|---|---|---|---|---|---|
| | | Accommodation management is perceived as a considerable addition to supervisors' regular duties, for which they feel ill-prepared, even where guidance is provided by others with the requisite expertise. [64] | | | | | | | |
| 4 | **Autonomy** - the individual's ability to have control or agency in relation to their pain and their work situation | Several respondents emphasized the importance of their possibilities to control what to do and when to do it and considered flexible working hours as a prerequisite for their return to work[38] | 12 13 28 30-32 37 38 40 41 43 47 | Well described (12) | Fit between underlying data and review finding is clear (12) | All CASP scores over 20 | Sweden 5<br>UK 4<br>Denmark 2<br>Canada 1 | Moderate confidence | Graded as moderate as well described and relevant across four cultures. |
| 5 | **Self-belief/ self-efficacy** - the individual's outlook about their ability to handle work and manage their pain | Self-efficacy statements pertaining to more complex work-related functions were subdivided into one of three categories based on the thematic content analysis: the ability to meet job demands, the ability to obtain help from others, and the ability to cope with pain.[59] | 12 28 30 38 40 47 48 51 56 59 63 | Richly described (18) | Fit between underlying data and review finding is very clear (11) | All CASP scores over 20 | Sweden 5<br>Denmark 1<br>Norway 2<br>UK 2<br>USA 1 | High confidence | Graded as high in relation to adequacy, coherence, methodological limitations and relevance |
| 6 | **Being believed** - people struggling with not being believed, trusted or | Employees typically discussed issues around being believed and trusted when they were ill. Managers, on the other hand, were more likely to | 28 31 39 41 52 56 59 61 65 | Well described (13) | Fit between underlying data and review finding is clear (8) | All CASP scores over 20 | Denmark 2<br>France 1<br>Sweden 2<br>UK 2<br>South Africa 1 | Moderate confidence | Graded as moderate as well described and relevant across five cultures. |

For peer review only

| | | | | | | | | | |
|---|---|---|---|---|---|---|---|---|---|
| | perceived as legitimate | talk about employees taking absence that was not legitimate, for example; *''People just don't turn up, you know. They phone in sick or er... The attitude is they, you know, 'why should I bother?' sort of thing. You get a lot of that.''* [Female manager] [65] | | | | | | | Employee and employer studies support this finding. |
| **7** | **Impact of and on the family** - the effects of chronic pain on family members and vice versa | Lots of patients with LBP are looked after by their family members who relieve them of physical activities. Many patients receive more attention and are encouraged to take rest. The positive feeling of being supported is counteracted by the negative feeling of uselessness, associated with being off work.[58] | 29 30 49 50 52 53 56 58 | Well described (13) | Fit between underlying data and review finding is clear (8) | All CASP scores over 20 | Australia 1<br>UK 3<br>Sweden 2<br>Switzerland 1<br>Norway 1 | Moderate confidence | Graded as moderate as well described and relevant across five cultures |
| **8** | **Not being understood** - this is in the context of relationships with health professionals | Participants felt that physicians did not understand their clients' work environment, such as what functional demands were necessary for them to complete their tasks as well as the psychosocial stressors that could cause their back pathology to become chronic.' [61] | 35 49 54 58 61 62 | Adequately described (9) | Some inconsistency in fit (6) | All CASP scores over 20 | Canada 2<br>Norway 2<br>Switzerland 1<br>South Africa 1 | Low confidence | Graded as low as some concerns about coherence and relevant across four cultures. Employee and employer studies support this finding. |

For peer review only

| | | | | | | | | | |
|---|---|---|---|---|---|---|---|---|---|
| **9** | **Finance and benefits** - financial difficulties and the economic insecurity of moving from welfare benefits back into work | Some patients who were unemployed on grounds of ill health had serious concerns about their financial future. They complained of sleep disorders and mental problems: *"Without medication I can't sleep. I don't know what is going to happen"* [58]<br><br>*"It must be possible to find transition solutions when trying to get back to work, solutions that make us feel economically secure. Otherwise, who would dare to try?"*[51] | 13 36 43 45 50 51 58 59 | Well described (10) | Fit between underlying data and review finding is clear (8) | All CASP scores over 20 | Canada 1<br>UK 1<br>USA 1<br>Switzerland 1<br>Sweden 3<br>Norway 1 | Moderate confidence | Graded as moderate as well described and relevant across six cultures |
| **10** | **Health and illness and pain representations impact on return to work** - the way people think about their pain and the mental representations they form in relation to beliefs about its cause and their perception of its impact on their lives | Beliefs about causality. All claimants reported work as the initial cause of their back pain condition, and most also perceived previous work/certain types of work (manual/heavy/repetitive) as a 'trigger' for subsequent episodes and therefore not conducive to return to work. [52]<br><br>Both patients and significant others in the non-working sample were resigned to the permanent | 28-31 33 34 41 46 47 52 56 58 62 | Richly described (22) | Fit between underlying data and review finding is very clear (13) | All CASP scores over 20 | Denmark 2<br>Australia 1<br>UK 3<br>Canada 3<br>Sweden 3<br>Switzerland 1 | High confidence | Graded as high in relation to adequacy, coherence, methodological limitations and relevance. Employee and employer studies support this finding. |

For peer review only

| | | | | | | | | | |
|---|---|---|---|---|---|---|---|---|---|
| | | effects of the patient's back problem on their employment status and were thus more likely to consider the patient as 'disabled', a role which might become self-fulfilling.' [30] | | | | | | | |
| 11 | **Meaning of work** - the meaning of work for an individual linked with motivation | Work was viewed by most patients as a source of financial security and a means of independence. However, the financial aspect of work did not seem to motivate all patients. A separate subgroup placed greater focus on health status and pain reduction strategies. This was a more prevalent attitude amongst those who had had longer durations of sick leave. For example, a patient that had been unemployed for more than one year stated: "*Money wouldn't be a motivator for me, I'd have to be well enough to return to work – that would be the motivator*"' .[47]<br><br>Participants who were placed in jobs that had no meaning to them caused | 38 40 41 44-48 50 54 57 61-65 | Richly described (26) | Fit between underlying data and review finding is very clear (14) | All CASP scores over 20 | Sweden 7<br>UK 2<br>Norway 2<br>Canada 2<br>South Africa 1 | High confidence | Graded as high in relation to relevance, coherence, adequacy & methodological limitations. Employee and employer studies support this finding. |

For peer review only

| | | | | | | | | | |
|---|---|---|---|---|---|---|---|---|---|
| | | them to become frustrated. When these meaningless tasks were coupled with relapses of back pain, there would be a downward spiral into depression and demotivation amongst the participants.' [61] | | | | | | | |
| 12 | **Mismatch between employee and employer expectations of return to work** | 'Meeting job demands typically referred to producing a certain quantity of work (e.g. 'I need to be at full capacity'), quality of work (e.g. 'I may not do a good job'), speed of work (e.g. 'I won't be able to keep up'), or fulfilling a particular role at work (e.g. 'I need to be able to respond to an emergency').' [59] | 13 38 40 47 48 51 55 59 60 64 | Well described (11) | Fit between underlying data and review finding is clear (10) | All CASP scores over 20 | Sweden 4<br>UK 2<br>Norway 2<br>USA 1<br>Canada 1 | Moderate confidence | Graded as moderate as well described and relevant across five cultures. Employee and employer studies support this finding. |
| 13 | **Social isolation as a consequence of chronic pain** - leading to a lack of support to return to work | 'Paid work – the pain sufferer's struggle for social capital. The informants were concerned about how the unpredictability of the pain broke into their daily lives and social contact with others, and challenged their normal way of dealing with everyday problems.'[54] | 29 31 50 54 56 | Adequately described (6) | Fit between underlying data and review finding is clear (5) | All CASP scores over 20 | Australia 1<br>Denmark 1<br>Sweden 2<br>Norway 1 | Low confidence | Graded as low as adequately described and relevant across four cultures |

For peer review only

| | | | | | | | | | |
|---|---|---|---|---|---|---|---|---|---|
| 14 | **Influence of return to work support and rehabilitation** | Support. The informants felt that the rehabilitation programme was the right place to come to when living with long-term pain. The team was described as empathetic and knowledgeable: when the informants told about their difficulties they felt understood for the first time. The informants also got support from each other, and a good feeling of fellowship developed; on the other hand, some also described how they were negatively affected by other participants who were depressed.' [56] | 27 28 31 33 35 37 38 41 47 50 56 58 59 | Well described (19) | Some inconsistency in fit (13) | All CASP scores over 20 | Denmark 3<br>Canada 3<br>Sweden 4<br>Switzerland 1<br>USA 1<br>UK 1 | Moderate confidence | Graded as moderate as well described and relevant across six cultures. |
| 15 | **System factors (healthcare, social security and workplace systems)** - how policies and procedures in these three systems impact on people with chronic pain | The way "systems" (dys)function delay return to work. When a worker becomes injured, they enter into complex relationships with the compensation system, unions, workplace, and health care system. How these systems interact with one other and with the injured worker can affect the RTW process. [62]<br><br>Within the healthcare system, participants talked | 13 32 36 37 39 41 51 56 57 61 62 65 | Richly described (25) | Fit between underlying data and review finding is very clear (12) | All CASP scores over 20 | UK 3<br>Canada 4<br>Sweden 2<br>Norway 1<br>South Africa 1<br>France 1 | High confidence | Graded as high in relation to relevance, coherence, adequacy & methodological limitations. Employee and employer studies support this finding. |

For peer review only

| | | | | | | | | | |
|---|---|---|---|---|---|---|---|---|---|
| | | of their frustrations with long waiting times, such as waiting for specialist appointments, diagnostic tests, treatments and entry into programs. [57]<br><br>'De-motivating economic arrangements for pensioners were also mentioned as a barrier. Some pointed to the fact that income under re-education was so low that the effort was not worthwhile. In addition, several had experienced that taking a small part time job when receiving pension would reduce the benefit so that nothing was gained economically. Finally, trying out for new jobs arranged by the job centre put them in an economically uncertain position and made them afraid of losing their disability benefit all together. More appropriate transitional arrangements were asked for.' [51] | | | | | | | |
| 16 | **Societal expectations** - expectations of | Experiences of societal expectations of participation in work. In | [49 38 48 61] | Adequately described (3) | Some inconsistency in fit | All CASP scores over 20 | Sweden 1 Norway 2 | Low | Graded as low as adequately described and |

For peer review only

| | | | | | | | | | |
|---|---|---|---|---|---|---|---|---|---|
| | family, friends and wider society that everyone should work resulting in judgement and discrimination against those who don't work | the societal discourse of work participation, inclusion in society was connected to employment. [49]<br><br>Some of the participants viewed their family and society as being judgemental, unsupportive and discriminatory, whereas others felt that they could not have rehabilitated themselves without the support of society. [61] | | | (4) | | South Africa 1 | | relevant across three cultures. |

N.B. No more than half female only studies supported any of the review findings.

For peer review only

**Line of argument**

A line of argument was constructed by examining how the conceptual categories relate to each other. A flow diagram/ conceptual model was then developed (Figure 2).

(**insert Figure 2 here**)

This conceptual model of return to work is now explained, going anti-clockwise from 1) to 7).

1) The underpinning foundation lies in the cultural expectation within society that people should work and contribute to the economy. Societal expectations are manifested within institutions, families and the media.
2) Societal and family expectations influence the individual's sense of self and what work means to each person. Meaning can relate to financial remuneration, rewards or survival, meeting of social, cognitive and achievement needs or purpose in life. The individual's level of self-belief and autonomy will both play a part in how much agency and control can be exerted over pain and the work situation.
3) The way someone thinks about their pain and the mental representation they create will also influence their behaviour and the possibility of returning to work. People's perceptions of whether pain is a long-term disability could influence whether they feel able to work and thus their return to work decisions, whereas someone who has accepted the pain as part of their life and adapts may be more likely to consider return to work.
4) Some studies in the review evaluated pre-return to work support or rehabilitation programmes and not being understood by health professionals was cited as an obstacle. In the same way, not being believed or being judged by people in the workplace was also perceived to make return to work challenging.
5) The three key tenets of return to work are managing pain, managing work relationships and making workplace adjustments. Tension exists between these three facets and they can be influenced by a mismatch between the individual

For peer review only

and the employer expectations, difficulties asking for help and system factors in the workplace, health and social security systems.

6) Individuals must negotiate a wide range of obstacles and navigate change.
7) This could result in a downward spiral (and not working) at one end of a continuum through to an upward spiral of mastering return to work despite pain.

## Discussion

In this meta-ethnography we identified obstacles to return to work for people with chronic pain centred around three key conceptual categories; managing pain, managing work relationships in the workplace and making workplace adjustments. The dynamic relationship between these three closely linked categories appears to be highly influential in navigating change and overcoming obstacles individuals with chronic pain face. The ability to manage pain and negotiate workplace adjustments can be affected by the strength of relationships with employer and colleagues and what is practicable in the work environment.

The concepts of health and pain representations and the role of significant others and their thinking about pain and return to work do not appear to be highlighted by previous reviews. Another neglected area is the influence of pre-return to work support or rehabilitation. The employer perspective is missing in earlier reviews which have focused on the experience of people with chronic pain. Only five of the included studies were conducted with employers so there is still limited research with this group despite people with pain emphasising the importance of employer attitudes and knowledge in the return to work process[55].

Some studies that were included in the review appear to suggest that those people with chronic pain who manage to stay in work have different characteristics to those who are unable to do so. This is seen in part to be connected with their cognitive appraisal of their pain and whether they are able to adapt.[33 47] It has been proposed that in those who do not return to work their pain representation of 'abnormal pain' becomes crystallised with their goal of pain elimination firmly intact whereas those who returned to work began to perceive

For peer review only

pain as ‘ the new normal’ and something they learn to live with.[33] Eden *et al.* (2007)[38] described three different adaptation patterns, the go-getter, realist and indifferent. They proposed the pessimistic and passive outlook of the latter type meant work return was less likely. Passivity in the interaction with stakeholders like the employer was found to be linked with reduced drive to return to work.[12] Angel *et al*. (2012) [28] and Dionne *et al*. (2013) [37] also found passivity in relation to pain was not helpful when addressing workplace obstacles.

The provision of professional individualised support and coaching in the workplace was seen to be valuable in the work return process [41] and this concept supports the idea of developing work based interventions to help people with chronic pain return to work.

When comparing findings with previous reviews that have highlighted obstacles to return to work, similarities include fears of not being able to fulfil employer expectations, not being believed by colleagues and financial concerns.[4] Worries for the future including financial and job security were also uncovered by MacNeela *et al*. (2015).[5] Strain on the family relationships including those with partners and children[4] and gender differences regarding role as carer or breadwinner were revealed.[5] Unsatisfying relationships with health professionals where people felt they were not being listened to and frustrations with limitations of medical treatment were another common feature.[5] Social withdrawal as a result of pain was highlighted in both of these reviews.[4] [5] A struggle for legitimacy with colleagues and stigma in the workplace was highlighted by Toye[7] and Froud[4]. This review also drew attention to the system not supporting return to work due to a lack of dialogue between employers, occupational health and the health system to facilitate a gradual return with appropriate adjustments.

The collaborative team approach to conceptual analysis increased the rigour of the review.[14] Independently drawing flow diagrams to illustrate the conceptual model and then coming together to amalgamate these through discussion and debate, combined with checking all concepts had been included, ensured this process was thorough.

For peer review only

The CERQual assessments indicated there was a high level of confidence in the findings for managing pain, managing work relationships, managing the workplace, self-belief, health and illness representations, the meaning of work and system factors. Although we have used CERQual, we found we agreed with many comments on its use by Toye[66], namely that for relevance, studies rated as partially or indirectly relevant could also contain helpful concepts. They suggest 'gravitational pull' of an idea may be important. They argue providing clear information about concepts is critical, and we have provided this in Supplementary Files 3 and 4. They also note for adequacy "The power of concepts to make us think, however, is not based on quantity of data included". We agree when looking at coherence that inconsistent findings do not necessarily call the findings into question. It may be one study has developed an insight not considered in other studies. No tool can guarantee confidence in findings, and authors still need to carefully consider issues rigour.

**Implications**

This review identifies obstacles faced by people with chronic pain in returning to work after a period of sick leave or unemployment and can be used to inform the development of a return to work intervention. The focus of such intervention should be working collaboratively with the person who has chronic pain and the employer to explore ways of addressing managing pain, managing work relationships and making workplace adjustments. The way in which the different factors work together either to enhance or inhibit return to work is highly individual and clinicians will need to assess what is most important for the person and employer with whom they are working. This intervention could be located in community/ primary healthcare and delivered by case managers, for example, occupational therapists or occupational health nurses working alongside general practitioners. Alternatively it could be delivered by employment specialists working in employment services and trained in pain management strategies. This type of intervention would provide support tailored to the specific needs of people with chronic pain. Discussion may be needed between the employer, the employee and the case manager to enable exploration of the ways in which obstacles to return to work

For peer review only

might be overcome. This collaborative approach has the potential to improve healthcare services and change workplace culture and is the kind of innovation envisioned by the UK government in their 10 year plan for people with long term health conditions to realise their working potential.[67]

## Limitations

It is apparent that more research is required from the employer perspective. The five studies included in the review were from the perspective of employers working in car making, university hospitals, home care provision for disabled people in France[39], public hospitals in France[42] and NHS Trust and local authority in Wales.[65] The Canadian study that included small and large employers did not specify the nature of the industry in which they were engaged.[62]

It is likely that the reviewers' backgrounds and experiences had an impact on synthesis findings. The authors came from health care professional and non-health care professional backgrounds, and these backgrounds and experiences of chronic pain provided certain lenses, which we would expect to influence our understanding.

At the time we did this work the eMerge Reporting Guidance for meta-ethnography [68] had not been published. They were published close to the end of the peer review process for this paper.

## Conclusions

The navigation of obstacles to return to work for people with chronic pain and their employers entails balancing the needs of the person with chronic pain, colleagues and the employing organisation. The influence of health and pain representations the person formulates has not been emphasised in previous reviews. Managing pain, managing relationships in the workplace and making adjustments are central to achieving a successful return to work and these can be hard work for the person with chronic pain.

For peer review only

For peer review only

**Acknowledgments**

We would like to thank Samantha Johnson, Academic Support Librarian (Medicine, Life Sciences & Psychology) at University of Warwick for her help with initial literature search and helpful guidance during the updated search process.

We would like to thank Debs Smith, patient and public involvement representative, who participated in developing the research protocol as a co-applicant.

**Author's contributions**

KS, RF and MU made a substantial contribution to the design of the study. MG and KS were responsible for acquisition and MG, KS and JOB were responsible for analysis and interpretation of the data. MG drafted the first, subsequent and final versions and KS, JOB, RF and MU revised all versions for important intellectual content and approved the final version. All authors agree to be accountable for the accuracy and integrity of the work.

**Competing interests**

RF is chief investigator on the Arthritis Research UK grant from which this project was funded. He has published multiple papers on chronic pain some of which are referenced in this paper.

RF and MU are part of an academic partnership with Serco Ltd related to return-to-work initiatives. RF and MU are directors and shareholders of Clinvivo Ltd, a university spin-out company that provides data collection services for health services research.

MU was Chair of the NICE accreditation advisory committee until March 2017 for which he received a fee. He is chief investigator or co-investigator on multiple previous and current research grants from the UK National Institute for Health Research, Arthritis Research UK and is a co-investigator on grants funded by the Australian NHMRC. He is an NIHR Senior Investigator. He has received travel expenses for speaking at conferences from the professional organisations hosting the conferences. He is a co-investigator on a study

For peer review only

receiving support in kind from Orthospace Ltd. He has accepted an honorarium from CARTA. He is an editor of the NIHR journal series, and a member of the NIHR Journal Editors Group, for which he receives a fee.

KS is an investigator in multiple previous and current research grants from UK National Institute for Health Research, Arthritis Research UK. She has received travel and accommodation expenses for speaking at conferences from the professional organisations hosting the conferences. She has published multiple papers on pain some of which are referenced in this paper.

Funding

This research is part of the RISE (Return to work with Individualised Supported Employment) feasibility study supported by the Arthritis Research UK charity (project number 9401) and the funder has had no involvement in this paper.

For peer review only

## References

1. Blyth FM, March LM, Brnabic AJM, et al. Chronic pain in Australia: a prevalence study. *Pain* 2001;89(2):127-34. doi: https://doi.org/10.1016/S0304-3959(00)00355-9

2. Goldberg DS, McGee SJ. Pain as a global public health priority. *BMC Public Health* 2011;11:770. doi: https://dx.doi.org/10.1186/1471-2458-11-770

3. Toye F SK, Hannink E, Barker K. A mega-ethnography of eleven qualitative evidence syntheses exploring the experience of living with chronic malignant pain. *BMC Medical Research Methodology* 2017;17:116-.

4. Froud R, Patterson S, Eldridge S, et al. A systematic review and meta-synthesis of the impact of low back pain on people's lives. *BMC Musculoskelet Disord* 2014;15:50.

5. MacNeela P, Doyle C, O'Gorman D, et al. Experiences of chronic low back pain: a meta-ethnography of qualitative research. *Health Psychology Review* 2015;9(1):63-82. doi: 10.1080/17437199.2013.840951

6. Johannes CB, Le TK, Zhou X, et al. The Prevalence of Chronic Pain in United States Adults: Results of an Internet-Based Survey. *The Journal of Pain* 2010;11(11):1230-39. doi: 10.1016/j.jpain.2010.07.002

7. Toye F, Seers K, Allcock N, et al. A synthesis of qualitative research exploring the barriers to staying in work with chronic musculoskeletal pain. *Disabil Rehabil* 2016;38(6):566-72.

8. de Vries HJ, Brouwer S, Groothoff JW, et al. Staying at work with chronic nonspecific musculoskeletal pain: a qualitative study of workers' experiences. *BMC Musculoskelet Disord* 2011;12:126. doi: http://dx.doi.org/10.1186/1471-2474-12-126

9. Patel AS, Farquharson R, Carroll D, et al. The Impact and Burden of Chronic Pain in the Workplace: A Qualitative Systematic Review. *Pain pract* 2012;12(7):578-89. doi: doi:10.1111/j.1533-2500.2012.00547.x

10. Henriksson CM, Liedberg GM, Gerdle B. Women with fibromyalgia: Work and rehabilitation. *Disability and Rehabilitation* 2005;27(12):685-95. doi: http://dx.doi.org/10.1080/09638280400009089

11. Wainwright E, Wainwright D, Keogh E, et al. Fit for purpose? Using the fit note with patients with chronic pain: a qualitative study. *Br J Gen Pract* 2011;61(593):e794-800. doi: http://dx.doi.org/10.3399/bjgp11X613133

12. Ahlstrom L, Dellve L, Hagberg M, et al. Women with Neck Pain on Long-Term Sick Leave. Approaches Used in the Return to Work Process: A Qualitative Study. *J Occup Rehabil Journal of Occupational Rehabilitation* 2017;27(1):92-105.

13. Patel S, Greasley K, Watson PJ. Barriers to rehabilitation and return to work for unemployed chronic pain patients: a qualitative study. *Eur J Pain* 2007;11(8):831-40.

14. Toye F, Seers K, Allcock N, et al. Meta-ethnography 25 years on: challenges and insights for synthesising a large number of qualitative studies. *BMC Medical Research Methodology* 2014b;14:80.

15. Noblit GW, Hare RD. Meta-Ethnography : synthesizing qualitative studies. Newbury Park, Calif. : Sage 1988.

16. Shaw RL, Booth A, Sutton AJ, et al. Finding qualitative research: an evaluation of search strategies. 2004;4(1):5. doi: 10.1186/1471-2288-4-5

17. CASP. Critical appraisal skills programme qualitative assessment tool 2018 [Available from: http://media.wix.com/ugd/dded87_29c5b002d99342f788c6ac670e49f274.pdf accessed 06 January 2018 2018.

18. Lewin S, Booth A, Glenton C, et al. Applying GRADE-CERQual to qualitative evidence synthesis findings: introduction to the series. *Implement Sci* 2018;13(Suppl 1):2. doi: 10.1186/s13012-017-0688-3 [published Online First: 2018/02/01]

19. Lewin S, Bohren M, Rashidian A, et al. Applying GRADE-CERQual to qualitative evidence synthesis findings-paper 2: how to make an overall CERQual assessment of confidence and create a

Summary of Qualitative Findings table. *Implement Sci* 2018;13(Suppl 1):10. doi: 10.1186/s13012-017-0689-2 [published Online First: 2018/02/01]

20. Glenton C, Carlsen B, Lewin S, et al. Applying GRADE-CERQual to qualitative evidence synthesis findings-paper 5: how to assess adequacy of data. *Implement Sci* 2018;13(Suppl 1):14. doi: 10.1186/s13012-017-0692-7 [published Online First: 2018/02/01]
21. Colvin CJ, Garside R, Wainwright M, et al. Applying GRADE-CERQual to qualitative evidence synthesis findings-paper 4: how to assess coherence. *Implement Sci* 2018;13(Suppl 1):13. doi: 10.1186/s13012-017-0691-8 [published Online First: 2018/02/01]
22. Munthe-Kaas H, Bohren MA, Glenton C, et al. Applying GRADE-CERQual to qualitative evidence synthesis findings-paper 3: how to assess methodological limitations. *Implement Sci* 2018;13(Suppl 1):9. doi: 10.1186/s13012-017-0690-9 [published Online First: 2018/02/01]
23. Noyes J, Booth A, Lewin S, et al. Applying GRADE-CERQual to qualitative evidence synthesis findings-paper 6: how to assess relevance of the data. *Implement Sci* 2018;13(Suppl 1):4. doi: 10.1186/s13012-017-0693-6 [published Online First: 2018/02/01]
24. Schutz A. Concept and theory formation in the social sciences. *Journal of Philosophy* 1954;51(9):270-73.
25. NVivo 11 [program]: QSR International Pty Ltd. , 2012.
26. Toye F, Seers K, Allcock N, et al. 'Trying to pin down jelly' - exploring intuitive processes in quality assessment for meta-ethnography. *BMC Medical Research Methodology* 2013b;13:46.
27. Andersen LN, Kohberg M, Herborg LG, et al. Here we're all in the same boat - a qualitative study of group based rehabilitation for sick-listed citizens with chronic pain. *SJOP Scandinavian Journal of Psychology* 2014;55(4):333-42.
28. Angel S, Jensen LD, Gonge BK, et al. Patients' interpretations of a counselling intervention for low back pain: a narrative analysis. *Int J Nurs Stud* 2012;49(7):784-92. doi: http://dx.doi.org/10.1016/j.ijnurstu.2012.01.010
29. Ashby S, Richards K, James C. The effect of fear of movement on the lives of people with chronic low back pain...including commentary by Carleton RN, Poulain C, Meyer K, and Glombiewski JA. *International Journal of Therapy & Rehabilitation* 2010;17(5):232-43.
30. Brooks J, McCluskey S, King N, et al. Illness perceptions in the context of differing work participation outcomes: exploring the influence of significant others in persistent back pain. *BMC Musculoskelet Disord* 2013;14 doi: 10.1186/1471-2474-14-48
31. Buus N, Jensen LD, Maribo T, et al. Low back pain patients' beliefs about effective/ineffective constituents of a counseling intervention: a follow-up interview study. *Disabil Rehabil* 2015;37(11):936-41. doi: 10.3109/09638288.2014.948135
32. Coole C, Watson PJ, Drummond A. Low back pain patients' experiences of work modifications; a qualitative study. *BMC Musculoskelet Disord* 2010;11:277. doi: https://dx.doi.org/10.1186/1471-2474-11-277
33. Coutu MF, Baril R, Durand MJ, et al. Transforming the meaning of pain: an important step for the return to work. *Work (Reading, Mass)* 2010;35(2):209-19.
34. Coutu MF, Baril R, Durand MJ, et al. Health and illness representations of workers with a musculoskeletal disorder-related work disability during work rehabilitation: a qualitative study. *J Occup Rehabil* 2011;21(4):591-600. doi: http://dx.doi.org/10.1007/s10926-011-9311-7
35. Coutu MF, Baril R, Durand MJ, et al. Clinician-patient agreement about the work disability problem of patients having persistent pain: why it matters. *J Occup Rehabil* 2013;23(1):82-92. doi: http://dx.doi.org/10.1007/s10926-012-9387-8
36. Crooks VA. Women's experiences of developing musculoskeletal diseases: Employment challenges and policy recommendations. *Disability and Rehabilitation* 2007;29(14):1107-16. doi: 10.1080/09638280600948193

For peer review only - http://bmjopen.bmj.com/site/about/guidelines.xhtml

37. Dionne C, Bourbonnais R, Frémont P, et al. Obstacles to and Facilitators of Return to Work After Work-Disabling Back Pain: The Workers' Perspective. *J Occup Rehabil* 2013;23(2):280-89. doi: 10.1007/s10926-012-9399-4
38. Edén L, Andersson HI, Ejlertsson G, et al. Characteristics of disability pensioners returning to work: an interview study among individuals with musculoskeletal disorders. *Disabil Rehabil* 2007;29(22):1720-26.
39. Fassier J-B, Durand M-J, Caillard J-F, et al. Results of a feasibility study: barriers and facilitators in implementing the Sherbrooke model in France. *Scand J Work Environ Health* 2015;41(3):223-33. doi: 10.5271/sjweh.3489
40. Gard G, Sandberg AC. Motivating factors for return to work. *Physiother Res Int* 1998;3(2):100-8.
41. Glavare M, Löfgren M, Schult M-L. Between unemployment and employment: Experience of unemployed long-term pain sufferers. *Work* 2012;43(4):475-85. doi: 10.3233/WOR-2012-1394
42. Grataloup M, Massardier-Pilonchéry A, Bergeret A, et al. Job Restrictions for Healthcare Workers with Musculoskeletal Disorders: Consequences from the Superior's Viewpoint. *J Occup Rehabil* 2016;26(3):245-52. doi: 10.1007/s10926-015-9609-y
43. Hansson M, Boström C, Harms-Ringdahl K. Living with spine-related pain in a changing society—a qualitative study. *Disability and Rehabilitation: An International, Multidisciplinary Journal* 2001;23(7):286-95. doi: http://dx.doi.org/10.1080/096382801750143625
44. Hansson M, Bostrom C, Harms-Ringdahl K. Sickness absence and sickness attendance--what people with neck or back pain think. *Soc Sci Med* 2006;62(9):2183-95.
45. Johansson EE, Hamberg K, Lindgren G, et al. 'How could I even think of a job?' - Ambiguities in working life in a group of female patients with undefined musculoskeletal pain. *Scand J Prim Health Care* 1997;15(4):169-74.
46. Juuso P, Skär L, Sundin K, et al. The Workplace Experiences of Women with Fibromyalgia. *MSC Musculoskeletal Care* 2016;14(2):69-76.
47. Kalsi P, Turkistani W, Sykes C, et al. "Work is a beautiful thing." Exploring attitudes towards employment in chronic pain (CP) patients attending a pain management programme (PMP). *Journal of Vocational Rehabilitation* 2016;44(1):97-107. doi: http://dx.doi.org/10.3233/JVR-150783
48. Kvam L, Eide AH, Vik K. Understanding experiences of participation among men and women with chronic musculoskeletal pain in vocational rehabilitation. *Work* 2013;45(2):161-74. doi: http://dx.doi.org/10.3233/WOR-121534
49. Kvam L, Vik K. Discourses of participation in work among men and women in vocational rehabilitation. *Journal of Occupational Science* 2015;22(3):345-57. doi: http://dx.doi.org/10.1080/14427591.2014.897204
50. Liedberg GM, Henriksson CM. Factors of importance for work disability in women with fibromyalgia: An interview study. *Arthritis Care and Research* 2002;47(3):266-74.
51. Magnussen L, Nilsen S, Råheim M. Barriers against returning to work--as perceived by disability pensioners with back pain: a focus group based qualitative study. *Disabil Rehabil* 2007;29(3):191-97.
52. McCluskey S, Brooks J, King N, et al. The influence of 'significant others' on persistent back pain and work participation: a qualitative exploration of illness perceptions. *BMC Musculoskelet Disord* 2011;12:236. doi: http://dx.doi.org/10.1186/1471-2474-12-236
53. McCluskey S, Brooks J, King N, et al. Are the treatment expectations of 'significant others' psychosocial obstacles to work participation for those with persistent low back pain? *Work* 2014;48(3):391-8. doi: http://dx.doi.org/10.3233/WOR-131789
54. Nilsen G, Anderssen N. Struggling for a normal life: Work as an individual self-care management strategy among persons living with non-malignant chronic pain. *Work* 2014;49(1):123-32. doi: 10.3233/WOR-131642

55. Nordqvist C, Holmqvist C, Alexanderson K. Views of Laypersons on the Role Employers Play in Return to Work When Sick-Listed. *J Occup Rehabil* 2003;13(1):11-20. doi: http://dx.doi.org/10.1023/A:1021841722613
56. Rydstad M, Schult M, Löfgren M. Whiplash patients' experience of a multimodal rehabilitation programme and its usefulness one year later. *Disabil Rehabil* 2010;32(22):1810-18. doi: 10.3109/09638281003734425
57. Saunders SL, MacEachen E, Nedelec B. Understanding and building upon effort to return to work for people with long-term disability and job loss. *Work* 2015;52(1):103-14. doi: http://dx.doi.org/10.3233/WOR-141977
58. Scheermesser M, Bachmann S, Schamann A, et al. A qualitative study on the role of cultural background in patients' perspectives on rehabilitation. *BMC Musculoskelet Disord* 2012;13:5. doi: https://dx.doi.org/10.1186/1471-2474-13-5
59. Shaw WS, Huang YH. Concerns and expectations about returning to work with low back pain: identifying themes from focus groups and semi-structured interviews. *Disabil Rehabil* 2005;27(21):1269-81.
60. Sjöström R, Melin-Johansson C, Asplund R, et al. Barriers to and possibilities of returning to work after a multidisciplinary rehabilitation programme. A qualitative interview study. *Work* 2011;39(3):243-50. doi: 10.3233/WOR-2011-1172
61. Soeker MS, Wegner L, Pretorius B. I'm going back to work: Back injured clients' perceptions and experiences of their worker roles. *Work* 2008;30(2):161-70.
62. Soklaridis S, Ammendolia C, Cassidy D. Looking upstream to understand low back pain and return to work: Psychosocial factors as the product of system issues. *Soc Sci Med* 2010;71(9):1557-66. doi: 10.1016/j.socscimed.2010.08.017
63. Svensson T, Mussener U, Alexanderson K. Sickness absence, social relations, and self-esteem: a qualitative study of the importance of relationships with family, workmates, and friends among persons initially long-term sickness absent due to back diagnoses. *Work* 2010;37(2):187-97. doi: https://dx.doi.org/10.3233/WOR-2010-1069
64. Williams-Whitt K, Kristman V, Shaw WS, et al. A Model of Supervisor Decision-Making in the Accommodation of Workers with Low Back Pain. *J Occup Rehabil* 2016;26(3):366-81. doi: 10.1007/s10926-015-9623-0
65. Wynne-Jones G, Buck R, Porteous C, et al. What happens to work if you're unwell? Beliefs and attitudes of managers and employees with musculoskeletal pain in a public sector setting. *J Occup Rehabil* 2011;21(1):31-42.
66. Toye F, Seers K, Barker K. A meta-ethnography of healthcare professionals' experience of treating adults with chronic musculoskeletal pain to improve the experience and quality of healthcare. *Health Serv Deliv Res* 2018;6(17)
67. Department of Work and Pensions DoH. Improving lives. The future of Work, Health and Disability: HMSO, 2017.
68. France EF, Cunningham M, Ring N, et al. Improving reporting of meta-ethnography: the eMERGe reporting guidance. *BMC Med Res Methodol* 2019;19(1):25. doi: 10.1186/s12874-018-0600-0 [published Online First: 2019/02/03]

**Figure 1 legend:**

**Title: Figure 1 - Flow chart illustrating search outcome**

Figure 1 legend: This flow chart illustrates the search outcome

For peer review only

**Figure 2 legend:**

**Title: Figure 2 – Conceptual model – The work of return to work**

**Figure 2 legend:** This conceptual model of return to work is explained in the text, going anti-clockwise from 1) to 7).

**Data Sharing Statement**

We have tried to include all relevant data for the qualitative systematic review in supplementary files. Any other reasonable requests will be considered on a case by case basis by Mary Grant (lead author) email: M.Grant.2@warwick.ac.uk=

For peer review only

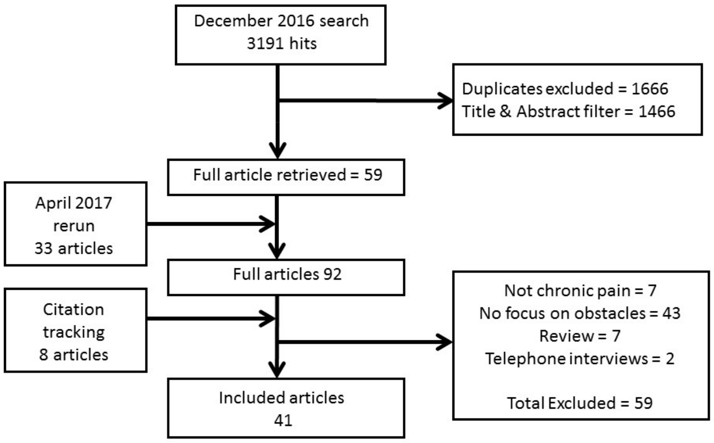


Figure 1 - Flow chart illustrating search outcome

108x60mm (300 x 300 DPI)

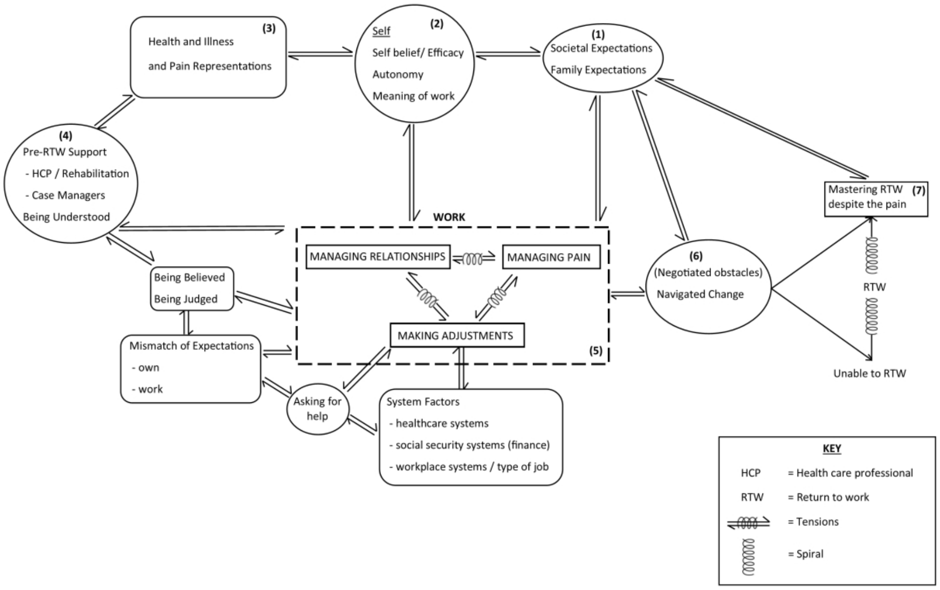


Figure 2 – Conceptual model – The work of return to work
This conceptual model of return to work is explained in the text, going anti-clockwise from 1) to 7).

297x209mm (300 x 300 DPI)

1 **Supplementary File 1 - Search strategy**

| | |
|---|---|
| **Search terms** | ("Chronic pain" (MeSH) OR (Chronic adj5 pain) OR "Pain")and ("Return to work (MeSH) OR Employment (MeSH) or Employer OR Supported Employment" OR "Return-to-work" OR "Back to work" OR "Back-to-work" OR "Reemployment" OR "Re-employment" OR "Job" or "Work" OR "Reentry" or "Re-entry" or "Back" or "Return" OR "Employment, Supported" (MeSH) OR "rtw") and "Qualitative" |
| **Databases searched** | AMED; ASSIA; CINAHL; EMBASE; IBSS; MEDLINE; PsycINFO; Social Services Abstracts; Sociological Abstracts; Web of Science; Westlaw<br><br>Forwards and backwards citation tracking using SCOPUS<br><br>Plus Social Care Online, PEDRO and OT Seeker |
| **Parts of journal searched** | Key words in abstract and title |
| **Years of search** | Inception to 25$^{th}$ April 2017 |
| **Language** | English |
| **Types of study to be included** | Qualitative peer reviewed studies using face-to-face interviews and focus groups |
| **Inclusion criteria** | Studies exploring perception of obstacles to return to work in off work, sick-listed and employer populations of people with chronic pain.<br>Studies of people on disability benefits. |
| **Exclusion criteria** | Non-English language texts. |

2

3

For peer review only

**Supplementary File 2 – Critical Appraisal Skills (CASP) scores – yes =3, can't tell = 2, no=1**

| | **CASP questions (question 10 is not scorable)** | | | | | | | | | |
|---|---|---|---|---|---|---|---|---|---|---|
| **Article authors** | **1** | **2** | **3** | **4** | **5** | **6** | **7** | **8** | **9** | **Total score** |
| Ahlstrom *et al.* 2017 | 3 | 3 | 3 | 2 | 3 | 1 | 3 | 3 | 2 | 24 |
| Andersen *et al.* 2014 | 3 | 3 | 3 | 3 | 3 | 1 | 3 | 3 | 3 | 27 |
| Angel *et al.* 2012 | 3 | 3 | 3 | 2 | 3 | 1 | 3 | 2 | 3 | 23 |
| Ashby *et al.* 2010 | 3 | 3 | 3 | 2 | 3 | 1 | 3 | 2 | 3 | 23 |
| Brooks *et al.* 2013 | 3 | 3 | 3 | 3 | 3 | 1 | 3 | 3 | 3 | 25 |
| Buus *et al.* 2015 | 3 | 3 | 3 | 3 | 3 | 3 | 3 | 3 | 2 | 26 |
| Coole *et al.* 2010 | 3 | 3 | 2 | 3 | 3 | 2 | 3 | 2 | 2 | 23 |
| Coutu *et al.* 2010 | 3 | 3 | 3 | 3 | 3 | 1 | 3 | 3 | 3 | 25 |
| Coutu *et al.* 2011 | 3 | 3 | 3 | 2 | 3 | 1 | 3 | 3 | 3 | 24 |
| Coutu *et al.* 2013 | 3 | 3 | 3 | 3 | 3 | 1 | 3 | 3 | 3 | 26 |
| Crooks 2007 | 3 | 3 | 3 | 2 | 3 | 1 | 3 | 3 | 3 | 24 |
| Dionne *et al.* 2013 | 3 | 3 | 3 | 2 | 3 | 1 | 3 | 3 | 3 | 24 |
| Edén *et al.* 2007 | 3 | 3 | 3 | 3 | 3 | 1 | 3 | 3 | 2 | 24 |
| Fassier *et al.* 2015 | 3 | 3 | 3 | 2 | 3 | 2 | 3 | 2 | 3 | 24 |
| Gard & Sandberg 1998 | 3 | 3 | 3 | 2 | 3 | 1 | 1 | 2 | 3 | 21 |
| Glavare *et al.* 2012 | 3 | 3 | 3 | 2 | 3 | 3 | 3 | 3 | 3 | 26 |
| Grataloup *et al.* 2016 | 3 | 3 | 3 | 3 | 3 | 1 | 3 | 3 | 3 | 25 |
| Hansson *et al.* 2001 | 3 | 3 | 3 | 2 | 3 | 1 | 3 | 3 | 3 | 24 |
| Hansson *et al.* 2006 | 3 | 3 | 3 | 3 | 3 | 1 | 3 | 3 | 3 | 25 |
| Johansson *et al.* 1997 | 3 | 3 | 3 | 3 | 3 | 3 | 1 | 3 | 3 | 25 |
| Juuso *et al.* 2016 | 3 | 3 | 3 | 3 | 3 | 2 | 3 | 3 | 3 | 26 |
| Kalsi *et al.* 2016 | 3 | 3 | 3 | 2 | 3 | 1 | 3 | 3 | 3 | 24 |
| Kvam *et al.* 2013 | 3 | 3 | 3 | 3 | 3 | 3 | 3 | 3 | 2 | 26 |

For peer review only

| | | | | | | | | | | |
|---|---|---|---|---|---|---|---|---|---|---|
| Kvam & Vik 2015 | 3 | 3 | 3 | 3 | 3 | 1 | 3 | 3 | 3 | 25 |
| Liedberg & Henriksson 2002 | 3 | 3 | 3 | 2 | 3 | 2 | 3 | 3 | 3 | 25 |
| Magnussen *et al.* 2007 | 3 | 3 | 3 | 3 | 3 | 2 | 2 | 2 | 3 | 24 |
| McCluskey *et al.* 2011 | 3 | 3 | 3 | 3 | 3 | 1 | 3 | 3 | 3 | 25 |
| McCluskey *et al.* 2014 | 3 | 3 | 3 | 3 | 3 | 2 | 3 | 3 | 3 | 26 |
| Nilsen & Anderssen 2014 | 3 | 3 | 3 | 3 | 3 | 1 | 3 | 3 | 3 | 25 |
| Nordqvist *et al.* 2003 | 3 | 3 | 3 | 3 | 3 | 1 | 1 | 3 | 3 | 23 |
| Patel *et al.* 2007 | 3 | 3 | 3 | 3 | 3 | 1 | 2 | 3 | 3 | 24 |
| Rydstad *et al.* 2010 | 3 | 3 | 3 | 3 | 3 | 3 | 3 | 3 | 3 | 27 |
| Saunders *et al.* 2015 | 3 | 3 | 3 | 3 | 3 | 2 | 3 | 3 | 3 | 26 |
| Scheermesser *et al.* 2012 | 3 | 3 | 3 | 3 | 3 | 3 | 3 | 3 | 3 | 27 |
| Shaw & Huang 2005 | 3 | 3 | 3 | 2 | 3 | 2 | 3 | 2 | 2 | 23 |
| Sjöström *et al.* 2011 | 3 | 3 | 3 | 3 | 3 | 2 | 3 | 2 | 3 | 25 |
| Soeker *et al.* 2008. | 3 | 3 | 3 | 3 | 3 | 2 | 3 | 3 | 3 | 26 |
| Soklaridis *et al.* 2010 | 3 | 3 | 3 | 2 | 3 | 2 | 3 | 3 | 2 | 24 |
| Svensson *et al.* 2010 | 3 | 3 | 3 | 3 | 3 | 1 | 3 | 2 | 3 | 24 |
| Williams-Whitt *et al.* 2016 | 3 | 3 | 3 | 2 | 3 | 1 | 3 | 3 | 3 | 24 |
| Wynne-Jones *et al.* 2011 | 3 | 3 | 3 | 3 | 3 | 1 | 3 | 3 | 3 | 25 |

For peer review only

## Supplementary file 3

The remaining 13 conceptual categories that underpin the three key categories described in the main paper and summarised in Table 2 are now explained.

### Societal expectations

Many people living with chronic pain felt that society, institutions, family, friends and the media expected them to work and that those who did not do so were portrayed negatively and perceived as a burden.[47] This contributed to them feeling they were outsiders who were judged and discriminated against and for some this motivated them to return to work so they could contribute and belong.[36 59]

### Meaning of work

The meaning of work for each individual plays a part in whether they return or not. For some work provides financial security and independence[45] but for others there is a strong moral work ethic influenced by their upbringing or it is a way of strengthening self-esteem[36] and achieving fulfilment in life.[48] A lack of meaning in work when combined with a chronic pain condition can lead to demotivation in relation to work return[59] whereas for some work was central to their identity and purpose and loss of this aspect of their lives was perceived as devastating.[39 42 44 52] Social contact and relationships with others and feeling needed and valued are an important aspect of work for some people.[48 61]

A number of conflicts were highlighted in this conceptual category. One was the differing perceptions of employees with low back pain and those of their employers. Some employers felt that employees perceived sick leave as a right and this formed part of a culture of entitlement they perceived was encouraged by the unions which made employees demotivated about returning to work.[60] However this was a view strongly contested by the employees in the same study who reported they would often accept modified jobs in order to return to work, even if not appropriate to them, due to the tough economic climate and fear of losing their livelihood.[60]

For peer review only

Another conflict was that of balancing the demands of work with those of family life. Competing priorities sometimes meant that some chose to focus on family rather than paid work as they were unable to balance the two.[46]

**Autonomy**

Concepts in this category focus on the individual's ability to have control or agency in relation to their pain and their work situation. There was a sense that if they had control over their pain then this was the key to having autonomy in other areas including return to work.[45] Being allowed some control and flexibility at work, for example in the hours they worked, was seen as a pre-requisite by some for returning.[36] Psychological distress, including anxiety and depression, was linked with a perceived lack of control over pain and as a result return to work became a secondary issue[12] whereas opportunity for job control was a motivating factor in relation to returning to work.[38]

**Self-belief/ self-efficacy**

Some studies indicated that people with chronic pain had low self-esteem and a pessimistic outlook about their ability to handle work[49] and this related to concerns about their ability to meet the job demands, obtain help from others and manage their pain.[57]

**Health and illness and pain representations impact on return to work**

The way people think about their pain and the mental representations they form in relation to beliefs about it's cause and their perception of its impact on their lives [29] are seen as very important in relation to their pain experience and return to work. A clear distinction was made between those who perceived themselves as disabled by the pain and therefore unable to work [28 31 32 44 50] and those who accepted the pain as part of their lives but something they could exert some control over and therefore felt able to work.[31 39 54] There was a perception amongst some employers that attitudes were influenced by family and the community in which people grew up.[60] However, among people with pain, there was a resistance to and even rejection of the idea of psychosocial influences on pain.[31 56] Some studies asserted

For peer review only - http://bmjopen.bmj.com/site/about/guidelines.xhtml

that the way people with pain, often incorrectly visualised their injuries and formulated explanatory models led to a fear of movement and this had negative implications for their work life.[27]

**Influence of pre-return to work support and rehabilitation**

A number of studies in the review were evaluating the impact of return to work interventions and rehabilitation programmes.[26 29 33 39 54] Participants were largely positive about the intervention received and the strategies for managing pain and life they had developed but those who did not return to work experienced anxiety, disappointment, loneliness [39] and some felt useless[56] or a sense of powerlessness and guilt.[26]

**Not being understood**

The concept of not being understood mainly arose when participants were describing difficulties in relationships with health professionals, for example physicians not understanding their work situation[59] or not listening or taking them seriously in relation to their pain.[52] However, the same phenomenon occurred even with people's closest relatives, leaving them with a sense of abandonment.[54]

**Being believed**

People with chronic pain often struggled with not being believed or trusted and this was evident when employers talked about people taking sick leave that was not perceived as legitimate.[63] This feeling of being judged and doubted and having to justify absence or limitations became an obstacle to returning to work.[37 59] The pursuit of authenticity also became apparent from the perspective of people claiming benefits.[50] These individuals felt the need to stress their desire to work but also to emphasise how the severity of their pain condition was preventing them from doing so.[50]

**Impact of and on family**

For peer review only

83 People with chronic pain often rely on family members for practical support[56 59] which leads to a renegotiation of roles and responsibilities[54] and financial difficulties are prevalent.[27]

85 Significant others are also seen to be highly influential in terms of their beliefs and thinking in relation to pain and return to work. Some studies have highlighted sceptical views of significant others in relation to treatment received and pessimism about return to work and support that would be offered[50 51] and sometimes their well-intentioned support reinforces a position of disability and legitimacy and this reduces the possibility of gaining employment.[28]

90 The conflicting demands of family and work were also reported as a challenge for people with chronic pain with women sometimes choosing to prioritise family.[48 47]

92 **Mismatch between employee and employer expectations of return to work**

93 Participants expressed a fear of letting employers down and not being able to fulfil work expectations.[12 58] Some were also fearful of re-injury.[57] Finding modified work was difficult and some felt the employer would rather dismiss them than find them a suitable job.[58] High demands for effectiveness and productivity made it difficult to return to work[49] as people with pain were concerned they would not be able to achieve the required quality, quantity or speed of work.[57] Information given to employers about health problems or limitations was perceived as insufficient or incorrect which led to misunderstandings and distorted employer expectations.[53] Difficulties arose in relationships with colleagues especially where expectations of employees with health problems were lower than for other employees doing the same job.[53] Participants felt they would want to do as much as their colleagues and not be a burden on them.[30 38 61]

104 **Social isolation as a consequence of pain**

105 One of the consequences of chronic pain was a withdrawal from social networks and this was partly linked with financial and physical restrictions.[27 48] This led to loneliness and a sense of being abandoned by those around and therefore lacking the support needed to enable return to work.[52 54]

For peer review only

**System factors (healthcare, social security and workplace systems)**

System factors influencing return to work were within healthcare, social security and workplace systems. Delays in accessing appropriate healthcare, for example waiting lists for specialists, diagnostic testing and rehabilitation programmes, interfered with the return to work process.[12 55 60] Another issue was that employers did not always feel they could accommodate injured workers hospital appointments in work time and so preferred that they remained off sick until they were able to fulfil their work hours.[60]

Social security authorities were sometimes seen as unhelpful and inflexible benefits arrangements caused economic uncertainty for people wanting to make a gradual transition into work.[49] Interactions with social insurance personnel were perceived as difficult with conflict arising when staff put pressure on people with pain to complete training or enter employment that was deemed unsuitable or not in line with their interests.[49 55]

Finally workplace systems delayed work return through inadequate policies[37] and a perceived lack of education on disability management procedures.[59] There also appeared to be a lack of trust in occupational health who were seen to be on the side of the employer and more concerned with absence management than supporting people to return to work.[30]

**Finance and benefits**

Many people with chronic pain had serious concerns about their finances.[41 43 56] For some this was linked with social security and disability benefits and the economic insecurity of moving back into work.[12 49] Some receiving state support struggled with shame whereas others felt they were entitled to it[43] or saw no alternative.[41] Some argued there should be greater flexibility for people with fluctuating musculoskeletal diseases to allow them to make a gradual return to work without incurring financial hardship.[34]

For peer review only

**Supplementary File 4 - Concepts within each conceptual category**

| Conceptual category | Concepts |
|---|---|
| Managing pain | 1. Adaptions via new knowledge of how to manage daily occupations<br>2. Being out in nature – pain management facilitator<br>3. Controlling pain<br>4. Coping with pain<br>5. Coping with symptoms<br>6. Developing individual strategies to deal with pain<br>7. Fatigue<br>8. Fluctuating work status<br>9. Keeping pain at bay<br>10. Knowledge of limits and listening to body<br>11. Learning to manage whiplash associated disorder - a rehabilitation process<br>12. Management of back pain<br>13. Previous management of back pain<br>14. Mastering life despite pain<br>15. New knowledge about how to manage daily life<br>16. Pain management linked with RTW<br>17. Pain management strategies<br>18. Pain representations underpin strategies to manage condition<br>19. Passive coping strategies<br>20. Patients' coping strategies<br>21. Physical activity<br>22. Strategies for managing long-term pain<br>23. Pain representations underpin strategies to manage condition<br>24. Passive coping strategies<br>25. Patients' coping strategies<br>26. Physical activity<br>27. Strategies for managing long-term pain<br>28. Strategies to prevent pain<br>29. Treatment for pain<br>30. Use of sick leave<br>31. When no treatment helps self-care management strategies develop<br>32. Working to control the pain<br>33. Being under control of pain and fear of pain<br>34. Commuting (as an obstacle to work)<br>35. Difficulties caused by back pain<br>36. Fear of reinjury<br>37. Impact of back pain on ability to work (for those at work and those not at work)<br>38. Impact of health on work<br>39. Impact of pain medication on ability to work<br>40. Impact of pain on doing work in a satisfactory way<br>41. Living with uncertainty<br>42. Negative impact of chronic pain on ability to work<br>43. Negative impact of pain on wellbeing and daily activities<br>44. Pain and somatic symptoms<br>45. Painful condition is a barrier to working<br>46. Persistent pain is an important obstacle to return to work |

For peer review only

| | |
|---|---|
| | 47. Return to employment and fear of movement<br>48. The body as an obstacle to working (pain and fatigue)<br>49. Unpredictable pain difficult with respect to work |
| Managing relationships | 1. Active engagement of supervisor<br>2. Asking for help is problematic for people with chronic pain<br>3. Attitude of employer in the workplace<br>4. Attitudes towards presenteeism (managers and employees perspectives)<br>5. Being needed at work<br>6. Colleague support important<br>7. Communication and contact (between managers and employees)<br>8. Co-workers and employers attitudes, disbelief and lack of understanding<br>9. Earlier negative workplace experiences<br>10. Employers limited understanding and support<br>11. Fear of disclosing pain to employer<br>12. Gap in work history and disclosure<br>13. Harassment from colleagues due to modified work<br>14. Impact of employees with job restrictions on supervisors and managers<br>15. Impact of sickness absence on others<br>16. Individuals (workers, colleagues, managers) barriers<br>17. Individuals facilitators (collaboration with colleagues support empathy problem solving mutual trust)<br>18. Interpersonal conflict with colleagues; being judged, justify the pain<br>19. Lack of collaboration and understanding from employer is an obstacle to return to work<br>20. Lack of communication between manager and the team about RTW<br>21. Lack of support and communication with line manager<br>22. Lack of support from line manager for injured workers<br>23. Lack of understanding from employer<br>24. Line manager role important<br>25. Maintaining contact with absent employee<br>26. Managerial attitude and effort<br>27. Managerial autonomy<br>28. Mutual distrust between employees and their managers and colleagues<br>29. Negative response from employer<br>30. Peer conflict<br>31. Physical barriers not significant obstacle to maintaining employment<br>32. Psychosocial environment (at work)<br>33. Psychosocial factors at work influence RTW management<br>34. Reassignment of workers to other areas due to physical demands of job causes tension with supervisors due to perceived injustice<br>35. Relationship between managers and employees<br>36. Relationship with employer<br>37. Relationships with supervisors and colleagues important to work satisfaction<br>38. Reluctance of employer to take on injured worker with gaps in employment history |

For peer review only

| | |
|---|---|
| | 39. Responsibility for workmates<br>40. Social tensions in the workplace<br>41. Stigmatisation and blame<br>42. Struggling interactions with stakeholders<br>43. Support from employer and workmates<br>44. Supportive work environment<br>45. Supportive work environment and manager key to RTW success<br>46. Sympathy from manager if fellow back pain sufferer<br>47. Treatment from line manager inequitable<br>48. Understanding from an employer<br>49. Work relationships influence RTW<br>50. Working relations<br>51. Workmates attitudes |
| Making workplace adjustments | 1. Being marked out as different in the workplace<br>Austerity and economic climate<br>2. Competitive economic climate with restructuring and workforce reduction is a barrier for RTW<br>3. Economic climate impacts on ability to take sick leave and make work adjustments<br>4. Fast management turnover, lack of latitude in decision making and fear of increasing costs and claims for better working conditions are barriers to RTW<br>5. Job availability and competitive job markets<br>6. Work restructuring (labour market)<br>Flexible working<br>7. Flexibility from employers re hours facilitates RTW<br>8. Flexible working a pre-requisite for RTW<br>9. Impossibility of a gradual return to work is an obstacle to return<br>10. Modified hours of employment<br>11. Policy and programme recommendations<br>12. Flexible work hours<br>13. Job sharing and work-from-home<br>14. Work place adjustments<br>Involve managers and colleagues<br>15. Communication quality<br>16. Maintaining routines for sharing information about work accommodations with colleagues<br>Manager knowing options<br>17. Absenteeism destabilises work organisation and makes work accommodations challenging<br>18. Accommodation demands<br>19. Job aptitude restriction certificates<br>20. Lack of pre-planning for RTW makes job accommodations and communication with colleagues challenging<br>21. Poor matching of the worker and the work<br>22. Process of accommodation of back injured workers<br>Resources for decision making about accommodations<br>23. Information - Accommodation options<br>24. Information - Employee abilities<br>25. Information - Job demands |

| | |
|---|---|
| | 26. Information - Medical restrictions<br>27. Organisational support<br>28. Supervisors return to work experience<br>29. Size of workplace and difficulties of modified duties<br>30. Support from line managers over-cautious<br>31. Work modifications - assistance from Occupational Health<br>32. Work organisation and the challenges of work accommodations<br>Not consulted or involved in decision making.<br>33. Modified duties<br>34. Perceived lack of choice and control in relation to modified duties<br>35. Possibility of work adaptations and confidence and ability to negotiate adaptations with employer<br>36. Stakeholder perspective<br>37. Psychosocial stressors<br>38. Work modifications - patient control<br>Personal factors<br>39. Age and educational status - perceived as obstacle to finding work by people with back injury<br>40. Personal obstacles - qualifications and experience<br>41. Personal obstacles to RTW - older age<br>42. Resistance to change<br>Reducing demands of job or physical adjustments<br>43. Adjustment of work demands upon return to work<br>44. Challenges to work participation (to different type of work and work adjustments)<br>45. Change to less physically demanding job<br>46. Impossibility of being assigned lighter duties and working at one's own pace is an obstacle to return to work<br>47. Improvement of work environment and working conditions<br>48. Physical accommodations<br>49. Provision of appropriate modified work can be challenging and complex<br>50. Work modifications influence RTW possibilities<br>51. Working conditions - physical work<br>Type of job influences RTW<br>52. Benefits of self-employment<br>53. Physical stressors (working at lower surface areas and different weights) cause constant pain<br>54. Sharing staff over different departments makes work accommodations and assessment of work demands difficult<br>55. Type of job- profession influences RTW |
| Autonomy | 1. Autonomy<br>2. In the hands of the professionals - reduced control over life situation<br>3. Increased job control and contact with supervisors<br>4. Influencing factors for RTW - internal and external<br>5. Lack of agency<br>6. Locus of control influences return to work<br>7. Making sense of intervention - regaining control of situation<br>8. Own agency is important |

For peer review only

| | |
|---|---|
| | 9. Own power and resources<br>10. Perceived lack of control influences ability to work<br>11. Psychological barriers to return to work (lack of control, anxiety, depression, loss of confidence, frustration)<br>12. Taking control of and responsibility for work and life situation |
| Self-belief/ self-efficacy | 1. Being needed at work<br>2. Changed self-image<br>3. Controlling RTW interactions with stakeholders (employer, health care, social insurance system, union, public employment service)<br>4. Low self- evaluation of work ability and low self esteem<br>5. Obtaining help at work<br>6. Patient identity (when working and not working)<br>7. Positive coping linked with RTW self-efficacy<br>8. Positive self-identity a beneficial consequence of employment<br>9. Psychological effects of chronic pain affect RTW confidence<br>10. Relationship with family influences self-confidence and esteem<br>11. Satisfaction with self-image; confidence<br>12. Self-confidence through working<br>13. Self-efficacy<br>14. Self esteem<br>15. Self-image in relation to work<br>16. Self-identity<br>17. Unintended consequences - physical bodily changes post-injury like weight gain affect emotional readiness to return to work<br>18. Work morale |
| Being believed | 1. Being judged by colleagues<br>2. Disbelief from physicians<br>3. Distrustful attitude of the medical profession<br>4. Feeling doubted<br>5. Having to justify pain condition in the workplace<br>6. Legitimacy of absence and perceptions of others<br>7. Legitimising back pain<br>8. Legitimacy<br>9. Not being believed<br>10. Personally and socially legitimate explanations of LBP important<br>11. Pursuit of authenticity<br>12. Rights and responsibilities<br>13. Stigmatisation |
| Impact on and of family | 1. Being a 'good' significant other<br>2. Cultural differences in family support between women and men<br>3. Family support<br>4. Impact of family<br>5. Loss of social roles<br>6. Participation in work - a family matter<br>7. Relationship changes<br>8. Re-negotiation or loss of work role<br>9. Return to work is dependent on a cure (significant other viewpoint) |

For peer review only

| | |
|---|---|
| | 10. Role of significant others is important in return to work<br>11. Unpaid work (home, family, carer responsibilities)<br>12. Waiting for an answer (diagnosis, treatment, cure) (significant other viewpoint)<br>13. We have come to the end of the road (treatment options exhausted) (significant other viewpoint) |
| Not being understood | 1. Communication in rehab - cultural differences cause problems<br>2. Language barriers<br>3. Talking at cross purposes with health professionals<br>4. Congruence between clinicians understanding of workers representations and actual worker's representations during work rehab<br>5. Cultural factors influence RTW - family attitudes, language barriers, cultural beliefs<br>6. Differences between clinical judgement and workers representations during work rehab<br>7. Difficult to explain the pain<br>8. Lack of client centredness<br>9. Medical discourse of work participation - focus on pain rather than RTW |
| Finance and Benefits | 1. Finances<br>2. Financial concerns<br>3. Financial - job security<br>4. Interaction with benefits organisation<br>5. Permitted work<br>6. Limited staff skills in benefits organisation<br>7. Looking for a different way of living - transition to disability pension<br>8. Need for financial security<br>9. Part-time work by people receiving disability income assistance<br>10. State as supporter |
| Health and illness and pain representations | 1. Acceptance challenges - Difficulties in acceptance of pain and limitations impacts on participation in work<br>2. Acceptance of chronic pain as a long term disability is a barrier to return to work<br>3. Acceptance of limitations<br>4. Acceptance of pain as part of life facilitates RTW<br>5. Accepting the inability to work<br>6. Barriers to rehabilitation<br>7. Cultural influence on psychological factors<br>8. Beliefs about causality of back pain<br>9. Beliefs about course of illness and the sick role<br>10. Beliefs about treatment and effective management of LBP<br>11. Cause and meaning of back pain<br>12. Crystallising the abnormal pain representation<br>13. Cultural factors influence RTW - family attitudes, language barriers, cultural beliefs<br>14. Cultural factors influencing return to work (language and passive coping strategies) |

For peer review only

| | |
|---|---|
| | 15. Explanatory models of illness<br>16. Illness beliefs coherent<br>17. Impact of pain representations on return to work<br>18. Integrating explanations into daily life<br>19. Loss of ability<br>20. Loss of hope<br>21. Need to construct their own models of pain<br>22. Resignation to permanent effect of back problem on employment status in those not working and their significant others |
| Meaning of work | 1. Competing priorities mean work not necessarily prioritised<br>2. Effort to remain in or return to pre-injury jobs<br>3. Effort to return to employment following job loss<br>4. Employee motivation<br>5. Family orientated considerations take priority over working<br>6. Fulfilment in a work role<br>7. Goal orientated participation (work related achievements and values)<br>8. Importance of work<br>9. Lack of meaning and satisfaction in work<br>10. Meaning of work<br>11. Meaningful job- highly needed by others<br>12. Mentality (outlook) in relation to determination to RTW<br>13. Moral aspects of absence and presenteeism<br>14. Moral stance - importance of work<br>15. Motivation and entitlement<br>16. Motive for RTW<br>17. Organised time structure difficult to maintain without work<br>18. Participation constantly changing (feminine perspective)<br>19. Positive perceptions of work<br>20. Prioritising of work and home is an issue<br>21. Regaining identity (as a worker)<br>22. Sense of coherence<br>23. Sense of coherence and involvement in work, friends and family<br>24. Social aspects of work<br>25. Work as a source of security and independence<br>26. Work on hold |
| Mismatch of expectations | 1. Ability to do as much work as others<br>2. Fear of letting employers down<br>3. Fear of re-injury<br>4. Insufficient or incorrect information about health problems of the returning employee<br>5. Meeting job demands<br>6. Not able to fulfil work requirements<br>7. Own expectations in relation to RTW (optimistic or pessimistic)<br>8. Participating at before (masculine perspective)<br>9. Support expectations<br>10. Workplace productivity demands<br>11. Workplace support |

For peer review only

| | |
|---|---|
| Social isolation as a consequence of chronic pain | 1. Abandoned by those around (family, friends and colleagues)<br>2. Feeling on their own<br>3. Impact on social relations - many women with fibromyalgia fail to maintain social network due to demands of work and family<br>4. Loneliness in pain<br>5. Paid work - the struggle for social capital<br>6. Social isolation |
| Influence of return to work support and rehabilitation | 1. "A light at the end of the tunnel"; support, hope, new knowledge<br>2. A light in the tunnel - experience of work rehab programme<br>3. Hope of returning to work through rehab<br>4. Support<br>5. Believing in the intervention - effectiveness of exercise to manage LBP<br>6. Close social network – family, rehab team<br>7. Difficulty accessing worker representations and problem targeting in work rehab<br>8. Feelings about outcome of rehab<br>9. Function-centred treatment<br>10. Patients' expectations of treatment<br>11. Goals of rehabilitation - patient's perspective<br>12. Goals of rehabilitation - return to work<br>13. Joining a physical exercise programme<br>14. Lack of access to information or support groups is an obstacle to return to work<br>15. Mismatch of goals - patient and programme<br>16. Rehabilitation by activity and exercise<br>17. Specialised vocational rehab support needed<br>18. Support is important<br>19. Unsuccessful responses to intervention (rehabilitation) |
| System factors | *1. Health care system*<br>2. Healthcare barriers<br>3. Lack of communication, lack of coordination and fear of communication within compensation and health care systems is a barrier to RTW<br>4. Lack of knowledge in primary care and no support from social insurance office<br>5. Slowness of health care system is an obstacle to return to work<br>*6. Social security, insurance, unemployment office system*<br>7. From Social Insurance Office<br>8. From unemployment office<br>9. Inefficiency of the insurance companies<br>10. Lack of support from social security authorities<br>*11. Workplace system*<br>12. Inadequate workplace policy<br>13. Lack of education on disability management procedures by employers and rehabilitation professionals<br>14. Occupational health is for employers not employees<br>15. OH employer orientated - unequal relationship<br>16. Organisational policies - Return to work policies |

For peer review only

| | |
|---|---|
| | 17. Poor communication between stakeholders (doctors and employers about back condition and lighter duties duration)<br>18. Systems factors - workplace and union policies compensation system and healthcare system are barriers to RTW process<br>19. Wage support programmes awarded to employee<br>20. Workplace system barriers<br>21. External context barriers<br>22. Workplace barriers<br>23. Workplace system facilitators<br>24. External context facilitators<br>25. Workplace facilitators |
| Societal expectations | 1. Experiences of societal expectations of participation in work<br>2. Feeling of being outsider in society; work part of natural life<br>3. Unsupportive society |

For peer review only

**Enhancing transparency in reporting the synthesis of qualitative research: the ENTREQ statement**

| No | Item | Guide and description | Reported |
|---|---|---|---|
| **1** | Aim | State the research question the synthesis addresses. | p2 and p4 |
| **2** | Synthesis methodology | Identify the synthesis methodology or theoretical framework which underpins the synthesis, and describe the rationale for choice of methodology *(e.g. meta-ethnography, thematic synthesis, critical interpretive synthesis, grounded theory synthesis, realist synthesis, meta-aggregation, meta-study, framework synthesis).* | p5 |
| **3** | Approach to searching | Indicate whether the search was pre-planned (*comprehensive search strategies to seek all available studies)* or iterative (*to seek all available concepts until they theoretical saturation is achieved)*. | p5 |
| **4** | Inclusion criteria | Specify the inclusion/exclusion criteria *(e.g. in terms of population, language, year limits, type of publication, study type).* | p5 |
| **5** | Data sources | Describe the information sources used (e.g. *electronic databases (MEDLINE, EMBASE, CINAHL, psycINFO, Econlit), grey literature databases (digital thesis, policy reports), relevant organisational websites, experts, information specialists, generic web searches (Google Scholar) hand searching, reference lists)* and when the searches conducted; provide the rationale for using the data sources. | p5 |
| **6** | Electronic Search strategy | Describe the literature search *(e.g. provide electronic search strategies with population terms, clinical or health topic terms, experiential or social phenomena related terms, filters for qualitative research, and search limits)*. | p5 |
| **7** | Study screening methods | Describe the process of study screening and sifting *(e.g. title, abstract and full text review, number of independent reviewers who screened studies).* | p7 |
| **8** | Study characteristics | Present the characteristics of the included studies *(e.g. year of publication, country, population, number of participants, data collection, methodology, analysis, research questions).* | p9-p17 |

For peer review only

| No | Item | Guide and description | Reported |
|---|---|---|---|
| **9** | Study selection results | Identify the number of studies screened and provide reasons for study exclusion *(e,g, for comprehensive searching, provide numbers of studies screened and reasons for exclusion indicated in a figure/flowchart; for iterative searching describe reasons for study exclusion and inclusion based on modifications t the research question and/or contribution to theory development).* | p7 |
| **10** | Rationale for appraisal | Describe the rationale and approach used to appraise the included studies or selected findings *(e.g. assessment of conduct (validity and robustness), assessment of reporting (transparency), assessment of content and utility of the findings).* | p6 |
| **11** | Appraisal items | State the tools, frameworks and criteria used to appraise the studies or selected findings *(e.g. Existing tools: CASP, QARI, COREQ, Mays and Pope*[25]*; reviewer developed tools; describe the domains assessed: research team, study design, data analysis and interpretations, reporting).* | p6 |
| **12** | Appraisal process | Indicate whether the appraisal was conducted independently by more than one reviewer and if consensus was required. | p6 |
| **13** | Appraisal results | Present results of the quality assessment and indicate which articles, if any, were weighted/excluded based on the assessment and give the rationale. | Supp file 1 |
| **14** | Data extraction | Indicate which sections of the primary studies were analysed and how were the data extracted from the primary studies? *(e.g. all text under the headings "results /conclusions" were extracted electronically and entered into a computer software).* | p6 |
| **15** | Software | State the computer software used, if any. | p6 |
| **16** | Number of reviewers | Identify who was involved in coding and analysis. | p6 |
| **17** | Coding | Describe the process for coding of data *(e.g. line by line coding to search for concepts).* | p6 |
| **18** | Study comparison | Describe how were comparisons made within and across studies *(e.g. subsequent studies were coded into pre-existing concepts, and new concepts were created when deemed necessary).* | p6-7 |

For peer review only

| No | Item | Guide and description | Reported |
|---|---|---|---|
| **19** | Derivation of themes | Explain whether the process of deriving the themes or constructs was inductive or deductive. | p7 |
| **20** | Quotations | Provide quotations from the primary studies to illustrate themes/constructs, and identify whether the quotations were participant quotations of the author's interpretation. | p21-30 |
| **21** | Synthesis output | Present rich, compelling and useful results that go beyond a summary of the primary studies (e.g. *new interpretation, models of evidence, conceptual models, analytical framework, development of a new theory or construct).* | p31-35 |

From Equator Network. Tong A, Flemming K McInnes E Oliver S & Craig J (2012) Enhancing transparency in reporting the synthesis of qualitative research: ENTREQ. BMC Medical Research methodology **12**:181

For peer review only
